# Zinc binding proteome of a phytopathogen *Xanthomonas translucens* pv. *undulosa*

**DOI:** 10.1098/rsos.190369

**Published:** 2019-09-25

**Authors:** Ankita Sharma, Dixit Sharma, Shailender Kumar Verma

**Affiliations:** Centre for Computational Biology and Bioinformatics, School of Life Sciences, Central University of Himachal Pradesh, Kangra, Himachal Pradesh 176206, India

**Keywords:** *Xanthomonas translucens*, Zn-binding proteins, *in silico*, Zn homeostasis, plant–pathogen interactions

## Abstract

*Xanthomonas translucens* pv. *undulosa* (*Xtu*) is a proteobacteria which causes bacterial leaf streak (BLS) or bacterial chaff disease in wheat and barley. The constant competition for zinc (Zn) metal nutrients contributes significantly in plant–pathogen interactions. In this study, we have employed a systematic *in silico* approach to study the Zn-binding proteins of *Xtu.* From the whole proteome of *Xtu*, we have identified approximately 7.9% of proteins having Zn-binding sequence and structural motifs*.* Further, 115 proteins were found homologous to plant–pathogen interaction database. Among these 115 proteins, 11 were predicted as putative secretory proteins. The functional diversity in Zn-binding proteins was revealed by functional domain, gene ontology and subcellular localization analysis. The roles of Zn-binding proteins were found to be varied in the range from metabolism, proteolysis, protein biosynthesis, transport, cell signalling, protein folding, transcription regulation, DNA repair, response to oxidative stress, RNA processing, antimicrobial resistance, DNA replication and DNA integration. This study provides preliminary information on putative Zn-binding proteins of *Xtu* which may further help in designing new metal-based antimicrobial agents for controlling BLS and bacterial chaff infections on staple crops.

## 1. Introduction

The bacterial diseases of the crop plants place a major restraint on crop production and result in significant global food production losses and food security [1,2]. *Xanthomonas translucens* pv. *undulosa* (*Xtu*) is a gram-negative bacterial pathogen of crop plants, wheat and barley [3]. The infection of *Xtu* on wheat and barley causes bacterial leaf streak (BLS) and bacterial chaff diseases. BLS disease is distributed worldwide and brings about 30–40% yield losses [4]. Wheat crop is one of the staple food crops which feed approximately 30% of the total population and act as a rich source of macro- and micro-nutrients. The nutrients acquisition by the pathogen and in response the nutrients immunity provided by the plant host play critical roles in plant–pathogen interactions [5]. Also, the bacterial pathogens have a complex association between the metabolic processes, regulation of expression and functioning of virulence factors [6–8]. The efficient utilization of the available nutrients is required by the pathogenic bacteria to survive inside the host milieu. The constant competition for the trace transition metal ions is one of the key factors at the traffic circle of nutrient metabolism and virulence [9,10]. The transition metal ions are necessary for the survival of all the living organisms. Approximately one-third of all proteins contain one or more metal ions as cofactor for their structural stability and functional activity [11].

The transition metal, zinc (Zn) represents the second most abundant metal cofactor after iron [10]. Zinc is stable as divalent cation (Zn^2+^) with complete filled outer shell *d*-orbital (3*d*^10^) and has no redox activity [12]. Zinc metal performs a variety of catalytic, structural and regulatory activities in a number of proteins [13]. Further, these activities assist in various biological and cellular processes like gene expression, biosynthesis of extracellular peptidoglycans and amino acids, reactive oxygen species (ROS) detoxifications, DNA repair, production of virulence-related proteins and antibiotic resistance [13–15]. Zinc ion is the highly competitive divalent metal ion of Irving–Williams series after copper (Cu) and therefore can easily replace the other metals from their cognate metalloenzyme [16]. The intracellular pool of ‘free’ Zn in the cells must be kept low because of the high chelating ability of Zn [17]. In bacteria, the Zn concentration may vary from the range of 0.1–1.0 mM [18]. Although zinc is an essential micronutrient, its higher concentrations result in significant toxicity to the bacterial cell [18]. Therefore, a great challenge for the bacterial pathogens is to procure an adequate concentration of Zn for maintaining their growth and survival during the infection. Previously, it is a known fact that intracellular Zn-binding, Zn-sensing and import or export of Zn ions helps in maintaining Zn homeostasis in bacteria [19–21]. Studies on Zn-homeostatic mechanisms regulation of most of the bacteria like *E*. *coli*, *B*. *subtilus*, *B*. *anthracis*, *Staphylococcus*, *Streptococcus* has been made earlier [22–27], which stated that Zn uptake and efflux system play roles in bacterial virulence. Earlier, a systematic study on zinc proteome of *E. coli* was made using the assay of radioactive Zn^2+^ binding on the total proteins fractionated by two-dimensional gel electrophoresis [28]. The report stated that most of the newly identified Zn-binding proteins do not have known Zn-binding motifs that were earlier identified in higher eukaryotes. Further, the usage of traditional experimental techniques is restricted for the prediction of metalloproteins at complete proteome level. This is due to time resolution, high cost, sensitivity and need of more expertise to prepare sample and to handle specialized equipments precisely [29,30]. Therefore, the call of the hour is to use new high-throughput technologies of post-genomic era for genome-wide identification of metalloproteins [15,31–33].

A systematic computational approach has been used in the current study to identify and characterize the Zn-binding proteins from the whole proteome of the *Xtu* strain 4699 [34]. Furthermore, the identified Zn-binding proteins were also checked for their probable involvement in plant–pathogen interactions and virulence. This primarily study provides us putative Zn-binding proteins which probably act as targets for controlling BLS.

## 2. Material and methods

### 2.1. Proteome extraction and identification of Zn-binding proteins

The whole proteome of *Xtu* was downloaded from Refseq National Centre for biotechnology Information (NCBI) server. The complete proteome has 3536 proteins and all these were examined for the presence of Zn-binding motifs using MetalPDB [35]. MetalPDB is a database of metalloproteins which provide features of metalloproteins and their minimal functional sites. The information of metalloproteins stored in MetalPDB is drawn from PDB, Pfam, CATH and SCOP databases. We have collected the information of Zn-binding proteins from MetalPDB and prepared a local dataset of Zn-binding proteins to perform stand-alone blastp search on the whole proteome of *Xtu* at expect value (e-value) 0.00001. The proteins which were found homologous to the Zn dataset of MetalPDB at e-value ≤ 0.00001 were further selected.

The short-listed proteins were modelled by protein homology/analogy recognition engine v. 2.0 (Phyre2) program [36]. This is done because three-dimensional structure of the proteins aid to determine its interactions with metal ions. Phyre2 server is built on hidden Markov model for creation of three-dimensional structure of the protein. The high-throughput modelled proteins, having confidence and query coverage more than or equal to 90% and 50%, respectively, were manually chosen. The modelled proteins were scanned for the putative Zn-binding structural motifs using metal ion binding site prediction and docking server (MIB) [37], which is built on a fragment transformation method. In this method, the query protein was aligned to the metal binding templates that were extracted from metal bound proteins present in PDB. The templates represent the local structure of metal binding residues within 3.5 Å. According to MIB server, a metal binding site had to contain a metal ion and at least two residues to quantify as a metal ion binding residues template. Each cluster, after sequence and structural similarity, acquires a particular score, which is used for prediction of metal binding sites. For the evaluation of sequence similarity, MIB server uses BLOSUM62 matrix, and for calculation of structural similarity root mean square deviation of C*_α_* atoms of the alignments was used. At more than 95% specificity threshold, MIB server predicts the Zn-binding sites with 94.8% accuracy and 71.1% sensitivity [37]. Ligplot^+^ visualization tool was further used to check the interactions of MIB docked Zn^2+^ metal ion with the protein [38]. We found that interacting residues and interaction radii provided by MIB vary in a wide range. Therefore, the interaction distance was raised from primary sphere (up to 3.5 Å) (provided by MIB server) to secondary sphere (5 Å). Also, it was stated earlier that second shell of interactions helps in stabilizing metal binding site, raises metal affinity and plays a role in determining physical properties of transition metal complexes [39–41]. Further, we have ignored the proteins which only bind to backbone (*C*_*α*_) chain atoms. The proteins which were found to interact with Zn^2+^ ion up to 5 Å were finally selected as putative Zn-binding proteins.

### 2.2. Functional annotation, gene ontology analysis and localization prediction of Zn-binding proteins

The selected Zn-binding proteins were explored for functional domains, family and super-families using different bioinformatics databases: InterProScan [42], Pfam [43] and NCBI-CDD [44]. The broad classification of these proteins was done by literature reviews of identified domains and families. Further, the clustergram was generated using MEGA6 [45], BioEdit [46] and EvolView [47] servers. To construct and visualize the gene ontology (GO) [48] based molecular function and biological process networks, a Cytoscape [49] plug-in ClueGO v. 2.3.3 [50] was used. In these networks, each node indicates particular GO terms (biological or molecular) and edge indicates connections between GO terms based on their gene association. The significance of the particular GO term was indicated by the size of that particular node. The statistical kappa score method [51] was used in order to determine the functional grouping of these identified GO terms. Further, the selected Zn-binding proteins were analysed for their subcellular localization using bioinformatics utilities viz. PSORTb, CELLO and SOSUI-GramN [52–54]. The consensus of these was taken in order to predict precise localization.

### 2.3. Prediction of putative Zn-binding proteins probably involved in plant–pathogen interactions

The predicted Zn-binding proteins were examined for their participation in plant–pathogen interactions using blastp search against the experimentally validated virulent and effector proteins of bacterial plant pathogens present in Pathogen–Host Interaction database (PHI-base) [55]. The proteins that were found homologous at e-value ≤ 0.0001 were short-listed and probably considered to play roles in plant–pathogen interactions. Further, these short-listed proteins were scanned for their secretory nature using neural network-based computational servers SignalP [56] TatP [57] and SecretomeP [58], respectively. The proteins showing presence of signal peptide, Tat motif or proteins having Sec score more than or equal to 0.5 were selected as probable secretory proteins. To avoid the false positive prediction of secretory Zn-binding proteins, we have checked the presence of transmembrane α-helix in these selected proteins using transmembrane hidden Markov model (TMHMM) [59] and hidden Markov model for topology prediction (HMMTOP) [60] servers. The proteins having single or no transmembrane helix were short-listed further and referred as putative secretory Zn-binding proteins.

## 3. Results

### 3.1. Zinc-binding proteins and their binding patterns presented by *Xtu* proteome

**Figure 1.**
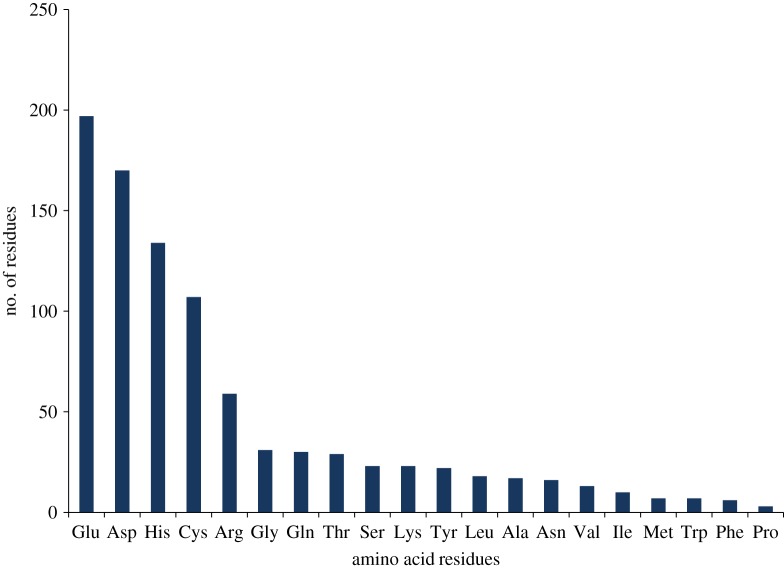
Interactions of Zn^2+^ metal ion with amino acid residues. The graph here represents the amino acid residues interacting with Zn^2+^ ion. The *X*-axis represents here the name of amino acid residues and *Y*-axis is showing number of amino acid residues. The frequently interacting residues with Zn metal ions were Glu, Asp, His and Cys.

Out of the complete proteome of *Xtu*, 346 proteins showed the occurrence of putative Zn-binding sequence motifs. Based on the modelling criteria and manual verification of structures' intactness, 335 proteins were short-listed further (electronic supplementary material, table S1). After Zn-protein interactions analysis by Liglpot^+^, 279 proteins showed interactions up to 5 Å which were selected further as putative Zn-binding proteins (electronic supplementary material, table S2). The interacting amino acid residues in Zn-binding proteins were Glu > Asp > His > Cys > Arg > Gly > Gln > Thr > Ser = Lys > Tyr > Leu > Ala > Asn > Val > Ile > Met = Trp > Phe > Pro (figure 1).

### 3.2. Functional annotation and cellular compartmentalization of predicted Zn-binding proteins

**Figure 2.**
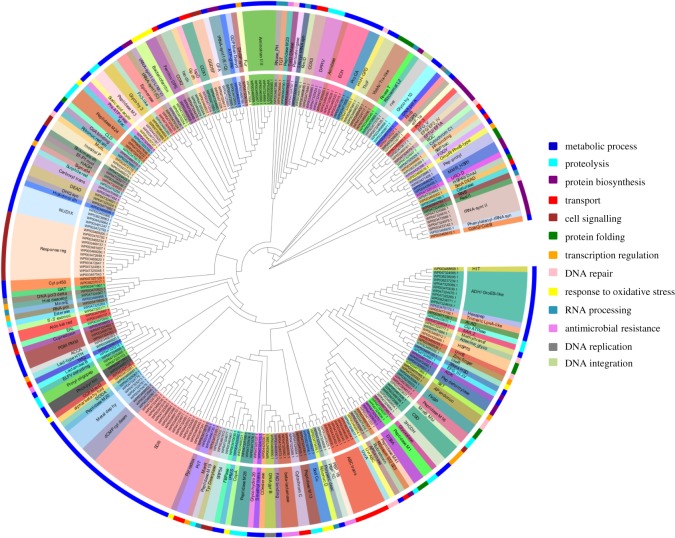
The functional classification of Zn-binding proteins. The functional classification of identified Zn-binding proteins was done on the basis of their domains. The clustergram was constructed by MEGA6 [45]. The inner circle of the clustergram represents sequence ID of the proteins. The middle and outer circle represent the functional domain and broad categories of the Zn-binding proteins. The most common domains in Zn-binding proteins of *Xtu* were SDR, response regulator, ADH/GroES, tRNA synthetase and ABC transporter. These proteins have diverse roles in metabolic processes, proteolysis, protein synthesis and transport.

**Figure 3.**
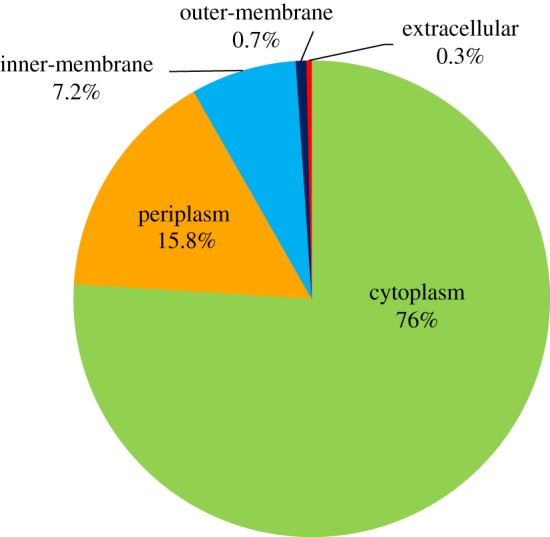
Subcellular localization of predicted Zn-binding proteins of *Xtu*. The pie chart shown here represents subcellular localization of predicted Zn-binding proteins of *Xtu*. Most of the proteins found to be localized in cytoplasm. Small number of proteins found in periplasm and inner-membrane. A little fraction of proteins found in outer-membrane and extracellular space.

The functional domain investigation of the scanned Zn-binding proteins showed the foremost existence of short chain dehydrogenase/reductase (SDR), response regulator receiver, alcohol dehydrogenase/GroES (ADH/GroES), tRNA synthetase and ABC transporter domains. Based on the literature studies of the identified domains, the Zn-binding proteins were widely classified into 13 classes. Most of the proteins associated with the classes of metabolic process (122), proteolysis (24), protein biosynthesis (22), transport (21), cell signalling (20), protein folding (13), DNA repair (12), transcription regulation (12), response to oxidative stress (11), RNA processing (9), antimicrobial resistance (7), DNA replication (4) and DNA integration (2). The detailed description is displayed in figure 2 and electronic supplementary material, table S3. The examination of cellular compartmentalization of short-listed Zn-binding proteins indicates that 76% of the proteins reside in cytoplasm, 15.8% in periplasm, 7.2% in inner-membrane, followed by outer-membrane (0.7%) and extracellular space (0.3%) (figure 3; electronic supplementary material, table S3).

### 3.3. Gene ontology network analysis of Zn-binding proteins

**Figure 4.**
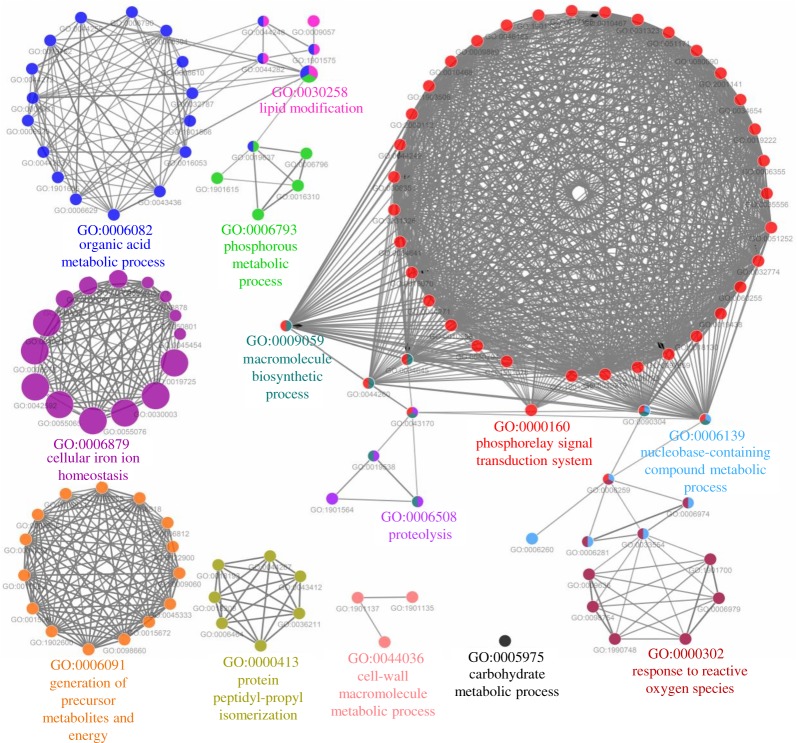
GO biological process network of Zn-binding proteins of *Xtu*. The ClueGO biological process network of Zn-binding proteins was constructed at kappa score ≥0.4. The circle here represents the node which indicates particular GO biological term. The colour of the node represents the particular GO group and mixed colour of the node indicates that node belongs to multiple groups. A total of 13 kappa score groups were found in this network, out of which cellular iron ion homeostasis and lipid modification GO biological processes were most significant.

**Figure 5.**
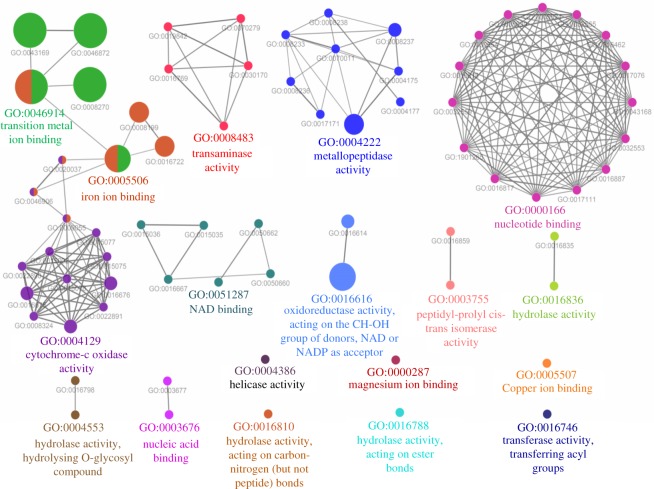
GO Molecular functional network of Zn-binding proteins of *Xtu.* The ClueGO molecular function network of Zn-binding proteins was built on kappa score ≥0.4. The circle here indicates the node with particular GO molecular term. The node colour represents the group to which they belong. The mixed coloured node indicates their presence in multiple molecular functions. A total of 18 kappa score groups were found in this network. The most significant groups were transition metal ion binding, iron ion binding, metallopeptidase activity and oxidoreductase activity.

The GO biological network of Zn-binding proteins was configured on 13 kappa score groups which contain 122 GO biological process terms and 623 GO biological terms connections (figure 4; electronic supplementary material, table S4). The GO terms which showed their presence in more than two functional groups indicate their roles in multiple biological processes. The GO biological terms cellular iron ion homeostasis and lipid modification represented the most significant groups in the GO biological process network. In order to check the involvement of Zn-binding proteins in biological processes, the analysis of the network was made and the number of interactions was estimated. The cellular macromolecule metabolic process (GO:0044260) and organic substance biosynthetic process (GO:1901576) were found as the most connected nodes with 69 links of each (electronic supplementary material, table S4). Further, to determine the molecular activities of scrutinized Zn-binding proteins the GO molecular function network was built on 18 kappa score groups. The network has 71 GO molecular function terms (nodes) and 136 connections (figure 5; electronic supplementary material, table S5). The most significant groups in the network were transition metal ion binding, iron ion binding, metallopeptidase activity and oxidoreductase acting on the CH–OH group of the donors and NAD or NADP as acceptor. The GO:0046872 and GO:0043169 were the most coupled GO molecular terms with 48 and 46 links of each, respectively (electronic supplementary material, table S5).

### 3.4. Zinc-binding proteins probably involved in plant–pathogen interactions

**Figure 6.**
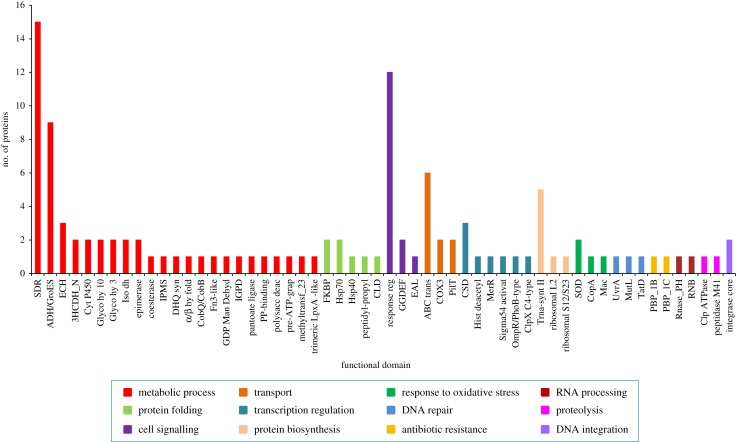
Functional domains of Zn-binding proteins probably involved in pathogen–host interactions. The graph here represents the functional domains of the Zn-binding proteins which are probably involved in pathogen–host interaction. The *X*-axis indicates functional domains and *Y*-axis shows number of proteins. SDR, response regulator, ADH/GroES, tRNA synthetase and ABC transporter were most common domains in these proteins. Colour of each bar indicate its broad functional class.

The Zn-binding proteins contribute in plant–pathogen interactions. Therefore, the selected 279 Zn-binding proteins were examined for their probable role in plant–pathogen interactions. Out of 279 proteins, 115 proteins were found homologous to PHI-base (electronic supplementary material, table S6). Among 115 proteins, 11 proteins were found to be secretory with single or no transmembrane helix (electronic supplementary material, table S7). These identified homologous Zn-binding proteins are probably considered to play important roles in bacterial virulence, survival and plant–pathogen interactions. The details of functional domains and categories of these proteins are given in table 1 and figure 6.

**Table 1.** Zn-binding proteins probably involved in pathogen–host interactions.

s. no.	sequence ID of putative Zn-binding protein	subcellular localization	functional domain/family	broad functional class	reference
1	WP_004426362.1	cytoplasmic	2-isopropylmalate synthase, bacterial-type	metabolic process	[61]
2	WP_003471682.1	cytoplasmic	3-dehydroquinate synthase	metabolic process	[62]
3	WP_003468654.1	cytoplasmic	3-hydroxyacyl-CoA dehydrogenase, NAD-binding domain	metabolic process	[63]
4	WP_047324684.1	cytoplasmic	3-hydroxyacyl-CoA dehydrogenase, NAD-binding domain	metabolic process	[63]
5	WP_003472081.1	periplasmic	alpha/beta hydrolase fold	metabolic process	[64]
6	WP_003465615.1	cytoplasmic	CobQ/CobB/MinD/ParA nucleotide-binding domain	metabolic process	[65]
7	WP_038237017.1	cytoplasmic	cytochrome P450	metabolic process	[66]
8	WP_047325129.1	cytoplasmic	cytochrome P450	metabolic process	[66]
9	WP_003465522.1	cytoplasmic	enoyl-CoA hydratase/isomerase	metabolic process	[67]
10	WP_003468480.1	cytoplasmic	enoyl-CoA hydratase/isomerase	metabolic process	[67]
11	WP_047325628.1	cytoplasmic	enoyl-CoA hydratase/isomerase	metabolic process	[67]
12	WP_003465369.1	cytoplasmic	fibronectin type III-like domain	metabolic process	[68]
13	WP_003466789.1	cytoplasmic	GDP-mannose 4,6 dehydratase	metabolic process	[69]
14	WP_003469871.1	periplasmic	glycoside hydrolase family 10	metabolic process	[70–72]
15	WP_003469868.1	periplasmic	glycoside hydrolase family 10	metabolic process	[70–72]
16	WP_047325488.1	periplasmic	glycoside hydrolase family 3	metabolic process	[70–72]
17	WP_080964854.1	periplasmic	glycoside hydrolase family 3	metabolic process	[70–72]
18	WP_047324805.1	cytoplasmic	imidazoleglycerol-phosphate dehydratase	metabolic process	[73]
19	WP_003468529.1	cytoplasmic	isocitrate/isopropylmalate dehydrogenase	metabolic process	[74]
20	WP_047325244.1	cytoplasmic	isocitrate/isopropylmalate dehydrogenase	metabolic process	[74]
21	WP_047324950.1	cytoplasmic	NAD-dependent epimerase/dehydratase family	metabolic process	[75]
22	WP_047325529.1	cytoplasmic	NAD-dependent epimerase/dehydratase family	metabolic process	[75]
23	WP_047324797.1	cytoplasmic	pantoate ligase	metabolic process	[76]
24	WP_003468806.1	cytoplasmic	phosphopantetheine attachment site	metabolic process	[77]
25	WP_047325261.1	inner-membrane	polysaccharide deacetylase	metabolic process	[78]
26	WP_047324952.1	cytoplasmic	pre-ATP-grasp domain	metabolic process	[79]
27	WP_047324777.1	cytoplasmic	S-adenosyl-l-methionine-dependent methyltransferase	metabolic process	[80]
28	WP_003465242.1	cytoplasmic	short-chain dehydrogenase/reductase SDR	metabolic process	[81,82]
29	WP_003465445.1	cytoplasmic	short-chain dehydrogenase/reductase SDR	metabolic process	[81,82]
30	WP_003466959.1	cytoplasmic	short-chain dehydrogenase/reductase SDR	metabolic process	[81,82]
31	WP_003467341.1	cytoplasmic	short-chain dehydrogenase/reductase SDR	metabolic process	[81,82]
32	WP_003468804.1	cytoplasmic	short-chain dehydrogenase/reductase SDR	metabolic process	[81,82]
33	WP_003469170.1	cytoplasmic	short-chain dehydrogenase/reductase SDR	metabolic process	[81,82]
34	WP_003472018.1	cytoplasmic	short-chain dehydrogenase/reductase SDR	metabolic process	[81,82]
35	WP_003472098.1	cytoplasmic	short-chain dehydrogenase/reductase SDR	metabolic process	[81,82]
36	WP_004425521.1	periplasmic	short-chain dehydrogenase/reductase SDR	metabolic process	[81,82]
37	WP_047324452.1	cytoplasmic	short-chain dehydrogenase/reductase SDR	metabolic process	[81,82]
38	WP_047324671.1	cytoplasmic	short-chain dehydrogenase/reductase SDR	metabolic process	[81,82]
39	WP_047324755.1	cytoplasmic	short-chain dehydrogenase/reductase SDR	metabolic process	[81,82]
40	WP_047324951.1	cytoplasmic	short-chain dehydrogenase/reductase SDR	metabolic process	[81,82]
41	WP_047325420.1	cytoplasmic	short-chain dehydrogenase/reductase SDR	metabolic process	[81,82]
42	WP_047325583.1	cytoplasmic	short-chain dehydrogenase/reductase SDR	metabolic process	[81,82]
43	WP_003473108.1	cytoplasmic	trimeric LpxA-like	metabolic process	[83]
44	WP_003470036.1	cytoplasmic	alcohol dehydrogenase GroES-like domain	metabolic process	[84–86]
45	WP_038237239.1	cytoplasmic	alcohol dehydrogenase GroES-like domain	metabolic process	[84–86]
46	WP_038238095.1	cytoplasmic	alcohol dehydrogenase GroES-like domain	metabolic process	[84–86]
47	WP_038238771.1	cytoplasmic	alcohol dehydrogenase GroES-like domain	metabolic process	[84–86]
48	WP_047324470.1	cytoplasmic	alcohol dehydrogenase GroES-like domain	metabolic process	[84–86]
49	WP_047324585.1	cytoplasmic	alcohol dehydrogenase GroES-like domain	metabolic process	[84–86]
50	WP_047324619.1	cytoplasmic	alcohol dehydrogenase GroES-like domain	metabolic process	[84–86]
51	WP_047325089.1	cytoplasmic	alcohol dehydrogenase GroES-like domain	metabolic process	[84–86]
52	WP_047325265.1	cytoplasmic	alcohol dehydrogenase GroES-like domain	metabolic process	[84–86]
53	WP_004425530.1	periplasmic	carboxylesterase family	metabolic process	[87,88]
54	WP_003470615.1	cytoplasmic	cyclophilin-type peptidyl-prolyl cis-trans isomerase/CLD	protein folding	[89,90]
55	WP_004426584.1	periplasmic	FKBP-type peptidyl-prolyl cis-trans isomerase domain	protein folding	[91–93]
56	WP_038239202.1	periplasmic	FKBP-type peptidyl-prolyl cis-trans isomerase domain	protein folding	[91–93]
57	WP_047324842.1	cytoplasmic	HSP40/DnaJ peptide-binding	protein folding	[93,94]
58	WP_003468012.1	cytoplasmic	Hsp70 protein/DnaK	protein folding	[93,94]
59	WP_004426468.1	cytoplasmic	Hsp70 protein/DnaK	protein folding	[93,94]
60	WP_003471408.1	cytoplasmic	Peptidyl-prolyl cis-trans isomerase domain	protein folding	[91–93]
61	WP_003470942.1	cytoplasmic	EAL domain	cell signalling	[95]
62	WP_003471228.1	cytoplasmic	GGDEF domain	cell signalling	[96]
63	WP_047324417.1	cytoplasmic	GGDEF domain	cell signalling	[96]
64	WP_003465234.1	cytoplasmic	response regulator receiver domain	cell signalling	[97,98]
65	WP_003465620.1	cytoplasmic	response regulator receiver domain	cell signalling	[97,98]
66	WP_003466157.1	cytoplasmic	response regulator receiver domain	cell signalling	[97,98]
67	WP_003467543.1	cytoplasmic	response regulator receiver domain	cell signalling	[97,98]
68	WP_003469200.1	cytoplasmic	response regulator receiver domain	cell signalling	[97,98]
69	WP_003469280.1	cytoplasmic	response regulator receiver domain	cell signalling	[97,98]
70	WP_003470782.1	cytoplasmic	response regulator receiver domain	cell signalling	[97,98]
71	WP_003472647.1	cytoplasmic	response regulator receiver domain	cell signalling	[97,98]
72	WP_003472648.1	cytoplasmic	response regulator receiver domain	cell signalling	[97,98]
73	WP_003481607.1	cytoplasmic	response regulator receiver domain	cell signalling	[97,98]
74	WP_047324961.1	cytoplasmic	response regulator receiver domain	cell signalling	[97,98]
75	WP_047325048.1	cytoplasmic	response regulator receiver domain	cell signalling	[97,98]
76	WP_003466285.1	inner-membrane	ABC transporter	transport	[18,99]
77	WP_003466345.1	inner-membrane	ABC transporter	transport	[18,99]
78	WP_003468030.1	inner-membrane	ABC transporter	transport	[18,99]
79	WP_004425452.1	inner-membrane	ABC transporter	transport	[18,99]
80	WP_038238334.1	inner-membrane	ABC transporter	transport	[18,99]
81	WP_047325000.1	inner-membrane	ABC transporter	transport	[18,99]
82	WP_003469645.1	inner-membrane	cytochrome c oxidase subunit III	transport	[100,101]
83	WP_003471844.1	inner-membrane	cytochrome c oxidase subunit III	transport	[100,101]
84	WP_003466735.1	cytoplasmic	pilus retraction protein PilT	transport	[102]
85	WP_003471666.1	cytoplasmic	pilus retraction protein PilT	transport	[102]
86	WP_003467936.1	cytoplasmic	'cold-shock’ DNA-binding domain	transcription regulation	[103]
87	WP_003472086.1	cytoplasmic	'cold-shock’ DNA-binding domain	transcription regulation	[103]
88	WP_003488188.1	cytoplasmic	'cold-shock’ DNA-binding domain	transcription regulation	[103]
89	WP_047324567.1	cytoplasmic	histone deacetylase domain	transcription regulation	[104]
90	WP_003473181.1	cytoplasmic	MerR HTH family regulatory protein	transcription regulation	[105]
91	WP_047324520.1	cytoplasmic	Sigma-54 interaction domain	transcription regulation	[106]
92	WP_003468516.1	cytoplasmic	OmpR/PhoB-type DNA-binding domain	transcription regulation	[107]
93	WP_003468538.1	cytoplasmic	zinc finger, ClpX C4-type	transcription regulation	[108]
94	WP_003470669.1	cytoplasmic	ribosomal protein L2, bacterial/organellar-type	protein biosynthesis	[109]
95	WP_003469157.1	cytoplasmic	ribosomal protein S12/S23	protein biosynthesis	[110]
96	WP_047324810.1	cytoplasmic	tRNA synthetase class II core domain (G H P S and T)	protein biosynthesis	[111,112]
97	WP_047324986.1	cytoplasmic	tRNA synthetases class II (A)	protein biosynthesis	[111,112]
98	WP_003472142.1	cytoplasmic	tRNA synthetases class II (D K and N)	protein biosynthesis	[111,112]
99	WP_003472589.1	cytoplasmic	tRNA synthetases class II (D K and N)	protein biosynthesis	[111,112]
100	WP_047324979.1	cytoplasmic	tRNA synthetases class II (D K and N)	protein biosynthesis	[111,112]
101	WP_003466231.1	periplasmic	CopA/multicopper oxidase	response to oxidative stress	[113]
102	WP_003470527.1	periplasmic	copper/zinc superoxide dismutase (SOD)	response to oxidative stress	[114]
103	WP_038238706.1	periplasmic	copper/zinc superoxide dismutase (SOD)	response to oxidative stress	[114]
104	WP_047325709.1	cytoplasmic	maltose/galactoside acetyltransferase	response to oxidative stress	[115]
105	WP_003472119.1	cytoplasmic	MutL C-terminal dimerization domain	DNA repair	[116]
106	WP_003467943.1	cytoplasmic	TatD-related DNase	DNA repair	[117]
107	WP_047324617.1	cytoplasmic	UvrA	DNA repair	[118]
108	WP_003465625.1	outer-membrane	penicillin-binding protein 1B	antimicrobial resistance	[119,120]
109	WP_047324608.1	inner-membrane	penicillin-binding protein 1C	antimicrobial resistance	[119,120]
110	WP_003467386.1	cytoplasmic	RNase_PH	RNA processing	[121,122]
111	WP_080964784.1	cytoplasmic	RNB domain	RNA processing	[121,122]
112	WP_004425670.1	cytoplasmic	Clp ATPase	proteolysis	[123]
113	WP_003471337.1	inner-membrane	peptidase family M41	proteolysis	[124]
114	WP_003477578.1	cytoplasmic	integrase core domain	DNA integration	[125]
115	WP_047324635.1	cytoplasmic	integrase core domain	DNA integration	[125]

## 4. Discussion

The present study focuses on the bioinformatic identification of potential Zn-binding proteins within the whole proteome of *Xtu* and their putative roles in its physiology and virulence. We have found 279 putative Zn-binding proteins which represent 7.9% of the whole proteome of *Xtu*. Previously, it was reported that Zn proteome of an organism varies in range from 4% to 10% of the whole proteome [126]. The predicted Zn proteome of *Xtu* not only contain Zn-binding proteins which exist in proper coordination (biological relevant assembly), but also Zn-substituted proteins and proteins participate as Zn buffering system. Earlier, it was stated that cytosolic Zn-binding proteins, transporters localized in cytoplasmic membranes and sensors of cytoplasmic free Zn ions are the molecules involved in Zn-homeostatic mechanisms of a cell [17]. Glutamate, aspartate, histidine and cysteine were found as frequently interacting residues in the binding pocket of Zn-binding proteins. Earlier, it was known that oxygen atoms of glutamate and aspartate, nitrogen atom of histidine and sulfur atom of cysteine were commonly interacting with Zn metal ions [127]. Some predicted proteins have one or two coordinates, i.e. do not have stable coordination. This may be due to the reason that MetalPDB and MIB rely on asymmetric units rather than biologically relevant assemblies [35,37]. Secondly, it may be because some ligand atom (water, inorganic or organic molecule) may bind to metal ion for their stable coordination in the biological active form [12].

It was documented earlier that Zn-binding proteins act as a cofactor for all the six types of enzymes, and therefore contribute significantly in various metabolic and other cellular processes, i.e. DNA repair, cell signalling, ROS detoxification and antimicrobial resistance [13,28,128–132]. Zinc is also known to bind proteins which are involved in gene expression and regulation, like sigma factor interacting proteins, RNA polymerases, tRNA synthetases, some ribosomal proteins and transcriptional factors [133–137]. In our study, SDR, response regulator receiver, ADH/GroES, tRNA synthetase and ABC transporter domains were commonly found in selected Zn-binding proteins. Further, the broad classification of these proteins based on domain description indicates their primary roles in metabolism (carbohydrates, proteins, lipid, nucleotides, etc.), proteolysis, protein biosynthesis, transport, cell signalling, transcription regulation protein folding and response to oxidative stress. The GO biological process network was in accordance with the domain-based broad classification. This network was also found to be enriched with the process of metabolism, signal transduction, proteolysis, lipid modification, response to ROS, protein peptide propyl isomerization and cellular ion homeostasis. Further, the GO molecular function network supports the findings of functional domain and GO biological process network, which signifies that most of the proteins involved in the metabolism have molecular activities of transition metal binding, nucleotide binding and NAD binding [9]. Also predicted Zn-binding proteins having hydrolase and transaminase activities may contribute in metabolic processes [13]. The proteins having cytochrome oxidase, peptidyl-propyl cis-trans isomerase and metallopeptidase activity probably specify their roles in transport, protein folding and proteolysis, respectively [91,100,138]. The subcellular localization of the proteins also determines their biological function [139]. The cellular compartmentalization of the Zn-binding proteins satisfies the fact that most of the proteins localized in cytoplasm are involved in metabolism, proteolysis, protein biosynthesis and cell signalling. Majority of the transporter proteins reside in inner-membrane.

Further, it was well documented earlier that Zn-binding proteins have considerable roles in bacterial toxin synthesis, virulence, antimicrobial resistance and host–pathogen interactions [14,140,141]. Here, we have found 115 PHI-base homologous Zn-binding proteins that probably engage in virulence and survival of *Xtu*. The Zn ions co-regulate the functioning of secretory proteins and contribute in plant–pathogen interactions [10], so secretory Zn-binding proteins have also been identified and checked for their putative roles in bacterial pathogenicity. Eleven proteins were found to be putative secretory Zn-binding proteins which were homologous to PHI-base.

These 115 proteins were categorized into 12 broad classes on the bases of domain description and their putative role in virulence (figure 6 and table 1). A total of 53 proteins were found in the category of metabolic process, out of which 15 proteins have SDR and nine proteins ADH/GroES domains. Earlier it was documented that SDR acts as a scaffold for redox sensor system and controls metabolic routes, transcription, cell signalling and stress, which further contributes in bacterial adaptation and pathogenesis [81,82]. ADH requires Zn metal ion for its catalytic activity and plays significant roles in alcohol fermentation, stress tolerance and virulence of bacteria [84–86]. Five secretory Zn-binding proteins were found in the class of metabolic process having glycoside hydrolase (4) and carboxylesterase (1) domain. It was stated previously that some glycoside hydrolases play role in host–microbe interactions and the enzymatic activity of some glycoside hydrolases was inhibited by Zn^2+^ metal ion [70–72]. The carboxylesterase protein also has affinity for Zn ion [87] and its role to hydrolyse ester bonds and virulence of bacteria was also known earlier [88,142].

We have found seven proteins that belong to the category of protein folding; among these, five proteins were confined to cytoplasm having Hsp70/DnaK (2), Hsp40/DnaJ peptide (1), PPIase (1) and cyclophilin-type PPIase (1) domains. Two proteins were found putative secretory, out of seven. These two proteins are localized in periplasm and have FKBP-type PPIase domain. Earlier, Linke *et al.* [94] reported that Zn-centre II of DnaJ mediates the interactions between DnaJ and DnaK, which is crucial for closing the DnaK substrate binding site and for locking-in the substrate [94]. It was stated previously that PPIase activity of cyclophilin is inhibited by Zn^2+^ ion in mouse macrophages cell line [89]. Further, a crystal study on Mip protein which is a propylisomerase of *Legionella pneumonia* indicated that Zn^2+^ is required to mediate crystal contacts between the C-terminal FKBP domains of adjacent Mip dimmers [92]. Previous studies showed that all these domains are involved in catalysing the step of protein folding which helps the pathogens with stress adaptation, survival in harsh conditions and further aid in virulence [90,91,93].

A total of 15 cytoplasmic proteins were found in the category of cell signalling. Among these, 12 proteins have response regulator domain, two proteins have GGDEF and one protein has EAL domain. It was reported earlier that response regulator domain of two-component system is involved in cell-to-cell communication and adaptation to the different environment inside and outside host which is prerequisite for pathogenicity [97,98]. The role of GGDEF and EAL domains to mediate virulence of *Xanthomonas* has also been noted earlier [95,96].

Six inner-membrane ABC transporter proteins were identified in this study. Previously, it was known that ABC transporters are involved in import and export of Zn ions and help to maintain Zn homeostasis which contributes in bacterial virulence [18,99]. Two inner-membrane cytochrome C oxidase (COX3) proteins and two cytoplasmic pilus retraction proteins (PilT) have also been found in the category of transport. It was documented earlier that COX3 are present only in bacteria mainly in pathogenic bacteria and are critical for many anaerobic biological processes, colonizing low oxygen tissues, and biogenesis of oligomeric membrane proteins [101]. Also, the role of PilT proteins in twitching motility, cell adherence, biofilm formation and host colonization was known previously [102].

In the class of transcription regulation, we have found eight cytoplasmic Zn-binding proteins. Out of these, three proteins have Cold-shock DNA-binding domain (CSD). CSD containing proteins are evolutionarily conserved and extensively distributed nucleic acid binding proteins that aid in transcription regulation and are involved in numerous cellular processes like low-temperature adaptation, nutrients stress and cell growth. CSD proteins of plants have additional glycine-rich regions with CCHC-type zinc fingers. Kim *et al.* [143] reported that CSD proteins and glycine-rich RNA-binding proteins from *A. thaliana* help *E. coli* to grow and survive better in cold-shock condition, i.e. promote cold adaptation process [143]. The other five proteins in the category of transcription regulation have histone deacetylase, Mer_HTH, Sigma-54 interaction, OmpR/PhoB-type DNA-binding and ClpX C4-type domains. All these domains require Zn for their catalytic activities, and also the roles of these in the regulation of transcription, virulence and pathogenicity of bacteria were previously documented in various studies [104–108].

Seven cytoplasmic proteins were found in the category of protein biosynthesis. Five of having tRNA synthetase II, one has ribosomal L2 and one has ribosomal S12/S23 domain. It was previously found that Zn ion helps in structural stability of these identified domains, which are involved in protein biosynthesis and also act as targets for many biocontrol agents [109–111,144]. A total of four proteins were found in the class of response to oxidative stress. Out of these, three were periplasmic secretory Zn-binding proteins having domains copper/zinc superoxide dismutase domain (SOD) (2) and CopA (1). The roles of these domains in radical oxygen species (ROS) detoxification, Zn homeostasis and bacterial virulence and survival were listed in earlier studies [113,114]. A cytoplasmic galactoside acetyltransferase domain containing protein was also found in this class, which is known to aid in cellular detoxification by acetylating non-metabolizable pyranosides [115].

We have categorized three cytoplasmic proteins having domains UvrA, MutL and TatD in the category of DNA repair. Earlier, it was reported that Zn ion is involved in structure architecture of Zn-finger domain of UvrA protein, and the role of C-terminal Zn-finger domain of UvrA protein has been noticed in regulation of damage-specific DNA binding [118]. MutL domain at its C-terminal contains a Zn-binding loop, a binding site for clamp DnaN and an endonuclease active site, which are critical for mismatch repair [116]. The TatD DNase domain has 3′-5′ exonuclease activity which digests single-stranded DNA and contributes in H_2_O_2_-induced DNA repair [117].

A secretory outer-membrane penicillin-binding protein (PBP) 1B and an inner-membrane PBP_1C were categorized in the class of antimicrobial resistance. Earlier, it was documented that PBP proteins not only localized in inner-membrane but also in outer-membrane, require Zn ion for their structural stability and are involved in antimicrobial resistance [119,120]. Two cytoplasmic proteins having RNase_PH/S1/KH and RNB domain were grouped in the class of RNA processing. Previous studies showed that RNase/RNB ribonucleases are critically required for RNA degradation, RNA and protein quality control and stress response, which results in raising virulence of the pathogens [121,122].

A cytoplasmic Clp ATPase domain containing protein and an inner-membrane peptidase M41 protein were categorized in class of proteolysis. Prior studies provide evidence that Clp ATPase causes cell proteolysis and plays central roles in virulence, gene expression, stress response and antimicrobial resistance [123]. Also, the role of Zn metallopeptidase M41 was previously listed in proteolysis and virulence [124].

Two cytoplasmic integrase core domain containing proteins were listed in the category of DNA integration. Formerly, it was known that bacterial integrase mediates site-specific recombination between bacterial and host cell [125] and further aids in pathogenesis.

## 5. Conclusion

To conclude, this study represents the first inclusive *in silico* report on Zn-binding proteins of *Xtu*. The functional diversity of Zn-binding proteins of *Xtu* unveil the facts that these proteins are metabolically versatile and contribute in various cellular and biological processes. The overall study provides the putative Zn-binding proteins repository and symbolizes their probable roles in growth, development, survival, pathogenicity and defence activities of *Xtu*. The presented repository may serve as starting material for experimental analysis which further paves the way to get insight into their mechanistic role in plant–pathogen interactions. Furthermore, in future these Zn-binding proteins may act as targets for designing metal-based antimicrobial agents in order to improve overall crop yield.

## Supplementary Material

Supplementary tables S1-S7

Reviewer comments

## Supplementary Material

Phyre 2 structures

## Data accessibility

The whole proteome *Xanthomonas translucens* pv. *undulosa* was downloaded from: (ftp://ftp.ncbi.nlm.nih.gov/genomes/all/GCF/001/021/935/GCF_001021935.1_ASM102193v1).

## References

[1] Savary S, Ficke A, Aubertot JN, Hollier C 2012 Crop losses due to diseases and their implications for global food production losses and food security. Food Security 4, 519–537. (10.1007/s12571-012-0200-5)

[2] Sundin GW, Castiblanco LF, Yuan X, Zeng Q, Yang CH 2016 Bacterial disease management: challenges, experience, innovation and future prospects. Mol. Plant Pathol. 17, 1506–1518. (10.1111/mpp.12436) 27238249 PMC6638406

[3] Bragard C, Verdier V, Maraite H 1995 Genetic diversity among *Xanthomonas campestris* strains pathogenic for small grains. Appl. Environ. Microbiol. 61, 1020–1026. 16534952 10.1128/aem.61.3.1020-1026.1995 PMC1388384

[4] Adhikari TB, Gurung S, Hansen JM, Bonman JM 2012 Pathogenic and genetic diversity of *Xanthomonas translucens* pv. *undulosa* in North Dakota. Phytopathology 102, 390–402. (10.1094/PHYTO-07-11-0201) 22204654

[5] Fatima U, Senthil-Kumar M 2015 Plant and pathogen nutrient acquisition strategies. Front. Plant Sci. 6, 750 (10.3389/fpls.2015.00750) 26442063 PMC4585253

[6] Sharma D, Sharma A, Verma SK, Singh B 2018 Targeting metabolic pathways proteins of *Orientia tsutsugamushi* using combined hierarchical approach to combat scrub typhus. J. Mol. Recognit. 32, e2766 (10.1002/jmr.2766) 30343521

[7] Peyraud R, Cottret L, Marmiesse L, Gouzy J, Genin S 2016 A resource allocation trade-off between virulence and proliferation drives metabolic versatility in the plant pathogen *Ralstonia solanacearum*. PLoS Pathog. 12, e1005939 (10.1371/journal.ppat.1005939) 27732672 PMC5061431

[8] Dandekar T, Eisenreich W 2015 Host-adapted metabolism and its regulation in bacterial pathogens. Front. Cell. Infect. Microbiol. 5, 28 (10.3389/fcimb.2015.00028) 25870851 PMC4376003

[9] Fones H, Preston GM 2013 The impact of transition metals on bacterial plant disease. FEMS Microbiol. Rev. 37, 495–519. (10.1111/1574-6976.12004) 23020129

[10] Palmer LD, Skaar EP 2016 Transition metals and virulence in bacteria. Annu. Rev. Genet. 50, 67–91. (10.1146/annurev-genet-120215-035146) 27617971 PMC5125913

[11] Lu CH, Lin YF, Lin JJ, Yu CS 2012 Prediction of metal ion-binding sites in proteins using the fragment transformation method. PLoS ONE 7, e39252 (10.1371/journal.pone.0039252) 22723976 PMC3377655

[12] Krężel A, Maret W 2016 The biological inorganic chemistry of zinc ions. Arch. Biochem. Biophys. 611, 3–19. (10.1016/j.abb.2016.04.010) 27117234 PMC5120989

[13] McCall KA, Huang C-C, Fierke CA 2000 Function and mechanism of zinc metalloenzymes. J. Nutr. 130, 1437–1446. (10.1093/jn/130.5.1437S) 10801957

[14] Porcheron G, Garénaux A, Proulx J, Sabri M, Dozois CM 2013 Iron, copper, zinc, and manganese transport and regulation in pathogenic Enterobacteria: correlations between strains, site of infection and the relative importance of the different metal transport systems for virulence. Front. Cellular Infect. Microbiol. 3, 90 (10.3389/fcimb.2013.00090) 24367764 PMC3852070

[15] Sharma A, Sharma D, Verma SK 2018 *In silico* study of iron, zinc and copper binding proteins of *Pseudomonas syringae* pv. *lapsa*: emphasis on secreted metalloproteins. Front. Microbiol. 9, 1838 (10.3389/fmicb.2018.01838) 30186242 PMC6110883

[16] Foster AW, Osman D, Robinson NJ 2014 Metal preferences and metallation. J. Biol. Chem. 289, 28 095–28 103. (10.1074/jbc.R114.588145) 25160626 PMC4192464

[17] Colvin RA, Holmes WR, Fontaine CP, Maret W 2010 Cytosolic zinc buffering and muffling: their role in intracellular zinc homeostasis. Metallomics 2, 306–317. (10.1039/b926662c) 21069178

[18] Capdevila DA, Wang J, Giedroc DP 2016 Bacterial strategies to maintain zinc metallostasis at the host-pathogen interface. J. Biol. Chem. 291, 20 858–20 868. (10.1074/jbc.R116.742023) PMC5076499 27462080

[19] Wątły J, Potocki S, Rowińska-Żyrek M 2016 Zinc homeostasis at the bacteria/host interface—from coordination chemistry to nutritional immunity. Chem. Eur. J. 22, 15 992–16 010. (10.1002/chem.201602376) 27555527

[20] Choi S, Bird AJ 2014 Zinc'ing sensibly: controlling zinc homeostasis at the transcriptional level. Metallomics 6, 1198–1215. (10.1039/C4MT00064A) 24722954

[21] Blindauer CA 2015 Advances in the molecular understanding of biological zinc transport. Chem. Commun. 51, 4544–4563. (10.1039/C4CC10174J) 25627157

[22] Takahashi H et al. 2015 The dynamic balance of import and export of zinc in *Escherichia coli* suggests a heterogeneous population response to stress. J. R. Soc. Interface 12, 20150069 (10.1098/rsif.2015.0069) 25808337 PMC4424684

[23] Kandari D, Gopalani M, Gupta M, Joshi H, Bhatnagar S, Bhatnagar R 2018 Identification, functional characterization, and regulon prediction of the zinc uptake regulator (zur) of *Bacillus anthracis*: an insight into the zinc homeostasis of the pathogen. Front. Microbiol. 9, 3314 (10.3389/fmicb.2018.03314) 30687290 PMC6336718

[24] Ma Z, Gabriel SE, Helmann JD 2011 Sequential binding and sensing of Zn (II) by *Bacillus subtilis* Zur. Nucleic Acids Res. 39, 9130–9138. (10.1093/nar/gkr625) 21821657 PMC3241647

[25] Makthal N, Kumaraswami M 2017 Zinc'ing it out: zinc homeostasis mechanisms and their impact on the pathogenesis of human pathogen group A streptococcus. Metallomics 9, 1693–1702. (10.1039/C7MT00240H) 29043347 PMC5730477

[26] Grim KP, San Francisco B, Radin JN, Brazel EB, Kelliher JL, Solórzano PKP, Kim PC, McDevitt CA, Kehl-Fie TE 2017 The metallophore staphylopine enables *Staphylococcus aureus* to compete with the host for zinc and overcome nutritional immunity. MBio 8, e01281-17 (10.1128/mbio.01281-17) 29089427 PMC5666155

[27] Nies DH. 2019 The ancient alarmone ZTP and zinc homeostasis in *Bacillus subtilis*. Mol. Microbiol. 112, 741–746. (10.1111/mmi.14332) 31220391

[28] Katayama A, Tsujii A, Wada A, Nishino T, Ishihama A 2002 Systematic search for zinc-binding proteins in *Escherichia coli*. Eur. J. Biochem. 269, 2403–2413. (10.1046/j.1432-1033.2002.02900.x) 11985624

[29] Shi W, Chance MR 2011 Metalloproteomics: forward and reverse approaches in metalloprotein structural and functional characterization. Curr. Opin. Chem. Biol. 15, 144–148. (10.1016/j.cbpa.2010.11.004) 21130021 PMC3040278

[30] Lothian A, Hare DJ, Grimm R, Ryan TM, Masters CL, Roberts BR 2013 Metalloproteomics: principles, challenges, and applications to neurodegeneration. Front. Aging Neurosci. 5, 35 (10.3389/fnagi.2013.00035) 23882215 PMC3714543

[31] Sharma A, Sharma D, Verma SK 2017 Proteome wide identification of iron binding proteins of *Xanthomonas translucens* pv. *undulosa*: focus on secretory virulent proteins. Biometals 30, 127–141. (10.1007/s10534-017-9991-3) 28105572

[32] Sharma A, Sharma D, Verma SK. 2019 In silico identification of copper-binding proteins of *Xanthomonas translucens* pv. *undulosa* for their probable role in plant-pathogen interactions. Physiol. Mol. Plant Pathol. 106, 187–195. (10.1016/j.pmpp.2019.02.005)

[33] Verma SK, Sharma A, Sandhu P, Choudhary N, Sharma S, Acharya V, Akhter Y 2017 Proteome scale identification, classification and structural analysis of iron-binding proteins in bread wheat. J. Inorg. Biochem. 170, 63–74. (10.1016/j.jinorgbio.2017.02.012) 28231452

[34] Peng Z, Hu Y, Xie J, Potnis N, Akhunova A, Jones J, Liu Z, White FF, Liu S 2016 Long read and single molecule DNA sequencing simplifies genome assembly and TAL effector gene analysis of *Xanthomonas translucens*. BMC Genomics 17, 21 (10.1186/s12864-015-2348-9) 26729225 PMC4700564

[35] Andreini C, Cavallaro G, Lorenzini S, Rosato A 2013 MetalPDB: a database of metal sites in biological macromolecular structures. Nucleic Acids Res. 41, D312–D319. (10.1093/nar/gks1063) 23155064 PMC3531106

[36] Kelley LA, Mezulis S, Yates CM, Wass MN, Sternberg MJE 2015 The Phyre2 web portal for protein modeling, prediction and analysis. Nat. Protoc. 10, 845–858. (10.1038/nprot.2015.053) 25950237 PMC5298202

[37] Lin Y-F, Cheng C-W, Shih C-S, Hwang J-K, Yu C-S, Lu C-H 2016 MIB: metal ion-binding site prediction and docking server. J. Chem. Inf. Model. 56, 2287–2291. (10.1021/acs.jcim.6b00407) 27976886

[38] Laskowski RA, Swindells MB 2011 LigPlot+: multiple ligand-protein interaction diagrams for drug discovery. J. Chem. Inf. Model. 51, 2778–2786. (10.1021/ci200227u) 21919503

[39] Shook RL, Borovik AS 2010 Role of the secondary coordination sphere in metal-mediated dioxygen activation. Inorg. Chem. 49, 3646–3660. (10.1021/ic901550k) 20380466 PMC3417154

[40] Ngo V, da Silva MC, Kubillus M, Li H, Roux B, Elstner M, Cui Q, Salahub DR, Noskov SY. 2015 Quantum effects in cation interactions with first and second coordination shell ligands in metalloproteins. J. Chem. Theory Comput. 11, 4992–5001. (10.1021/acs.jctc.5b00524) 26574284 PMC4827603

[41] Dudev T, Lim C 2013 Competition among metal ions for protein binding sites: determinants of metal ion selectivity in proteins. Chem. Rev. 114, 538–556. (10.1021/cr4004665) 24040963

[42] Jones P et al 2014 InterProScan 5: Genome-scale protein function classification. Bioinformatics 30, 1236–1240. (10.1093/bioinformatics/btu031) 24451626 PMC3998142

[43] Finn RD et al 2016 The Pfam protein families database: towards a more sustainable future. Nucleic Acids Res. 44, D279–D285. (10.1093/nar/gkv1344) 26673716 PMC4702930

[44] Marchler-Bauer A et al 2015 CDD: NCBI's conserved domain database. Nucleic Acids Res. 43, D222–D226. (10.1093/nar/gku1221) 25414356 PMC4383992

[45] Tamura K, Stecher G, Peterson D, Filipski A, Kumar S 2013 MEGA6: molecular evolutionary genetics analysis version 6.0. Mol. Biol. Evol. 30, 2725–2729. (10.1093/molbev/mst197) 24132122 PMC3840312

[46] Hall TA 1999 BioEdit: a user-friendly biological sequence alignment editor and analysis program for Windows 95/98/NT. Nucleic Acids Symp. Ser. 41, 95–98.

[47] Zhang H, Gao S, Lercher MJ, Hu S, Chen WH 2012 EvolView, an online tool for visualizing, annotating and managing phylogenetic trees. Nucleic Acids Res. 40 W569–W572. (10.1093/nar/gks576) 22695796 PMC3394307

[48] Ashburner M et al 2000 Gene ontology: tool for the unification of biology. Nat. Genet. 25, 25–29. (10.1038/75556) 10802651 PMC3037419

[49] Shannon P, Markiel A, Ozier O, Baliga NS, Wang JT, Ramage D, Amin N, Schwikowski B, Ideker T 2003 Cytoscape: a software environment for integrated models of biomolecular interaction networks. Genome Res. 13, 2498–2504. (10.1101/gr.1239303) 14597658 PMC403769

[50] Bindea G et al 2009 ClueGO: a Cytoscape plug-in to decipher functionally grouped gene ontology and pathway annotation networks. Bioinformatics 25, 1091–1093. (10.1093/bioinformatics/btp101) 19237447 PMC2666812

[51] Huang DW et al 2007 The DAVID gene functional classification tool: a novel biological module-centric algorithm to functionally analyze large gene lists. Genome Biol. 8, R183 (10.1186/gb-2007-8-9-r183) 17784955 PMC2375021

[52] Yu C-S, Lin C-J, Hwang J-K 2004 Predicting subcellular localization of proteins for Gram-negative bacteria by support vector machines based on n-peptide compositions. Protein Sci. 13, 1402–1406. (10.1110/ps.03479604) 15096640 PMC2286765

[53] Imai K, Asakawa N, Tsuji T, Akazawa F, Ino A, Sonoyama M, Mitaku S 2008 SOSUI-GramN: high performance prediction for sub-cellular localization of proteins in Gram-negative bacteria. Bioinformation 2, 417–421. (10.6026/97320630002417) 18795116 PMC2533062

[54] Yu NY et al 2010 PSORTb 3.0: improved protein subcellular localization prediction with refined localization subcategories and predictive capabilities for all prokaryotes. Bioinformatics 26, 1608–1615. (10.1093/bioinformatics/btq249) 20472543 PMC2887053

[55] Urban M, Pant R, Raghunath A, Irvine AG, Pedro H, Hammond-Kosack KE 2014 The Pathogen-Host Interactions database (PHI-base): additions and future developments. Nucleic Acids Res. 43, D645–D655. (10.1093/nar/gku1165) 25414340 PMC4383963

[56] Petersen TN, Brunak S, Von Heijne G, Nielsen H. 2011 SignalP 4.0: discriminating signal peptides from transmembrane regions. Nat. Methods. 8, 785–786. (10.1038/nmeth.1701) 21959131

[57] Bendtsen JD, Nielsen H, Widdick D, Palmer T, Brunak S 2005 Prediction of twin-arginine signal peptides. BMC Bioinf. 6, 167 (10.1186/1471-2105-6-167) PMC1182353 15992409

[58] Bendtsen JD, Jensen LJ, Blom N, Von Heijne G, Brunak S. 2004 Feature-based prediction of non-classical and leaderless protein secretion. Protein Eng. Des. Select. 17, 349–356. (10.1093/protein/gzh037) 15115854

[59] Krogh A, Larsson B, von Heijne G, Sonnhammer EL 2001 Predicting transmembrane protein topology with a hidden Markov model: application to complete genomes. J. Mol. Biol. 305, 567–580. (10.1006/jmbi.2000.4315) 11152613

[60] Tusnady GE, Simon I 2001 The HMMTOP transmembrane topology prediction server. Bioinformatics 17, 849–850. (10.1093/bioinformatics/17.9.849) 11590105

[61] Koon N, Squire CJ, Baker EN 2004 Crystal structure of LeuA from *Mycobacterium tuberculosis*, a key enzyme in leucine biosynthesis. Proc. Natl Acad. Sci. USA 101, 8295–8300. (10.1073/pnas.0400820101) 15159544 PMC420388

[62] Chandran SS, Frost JW 2001 Aromatic inhibitors of dehydroquinate synthase: synthesis, evaluation and implications for gallic acid biosynthesis. Bioorg. Med. Chem. Lett. 11, 1493–1496. (10.1016/S0960-894X(01)00065-8) 11412967

[63] Kang Y, Zarzycki-Siek J, Walton CB, Norris MH, Hoang TT 2010 Multiple FadD acyl-CoA synthetases contribute to differential fatty acid degradation and virulence in *Pseudomonas aeruginosa*. PLoS ONE 5, e13557 (10.1371/journal.pone.0013557) 21042406 PMC2958839

[64] Sultana R, Tanneeru K, Guruprasad L 2011 The PE-PPE domain in mycobacterium reveals a serine *α*/*β* hydrolase fold and function: an *in-silico* analysis. PLoS ONE 6, e16745 (10.1371/journal.pone.0016745) 21347309 PMC3037379

[65] Huang L, Qin Y, Yan Q, Lin G, Huang L, Huang B, Huang W 2015 MinD plays an important role in *Aeromonas hydrophila* adherence to *Anguilla japonica* mucus. Gene 565, 275–281. (10.1016/j.gene.2015.04.031) 25881868

[66] McLean KJ, Belcher J, Driscoll MD, Fernandez CC, Le Van D, Bui S, Golovanova M, Munro AW. 2010 The *Mycobacterium tuberculosis* cytochromes P450: physiology, biochemistry & molecular intervention. Future Med. Chem. 2, 1339–1353. (10.4155/fmc.10.216) 21426022

[67] Tsuge T, Fukui T, Matsusaki H, Taguchi S, Kobayashi G, Ishizaki A, Doi Y 2000 Molecular cloning of two (R)-specific enoyl-CoA hydratase genes from *Pseudomonas aeruginosa* and their use for polyhydroxyalkanoate synthesis. FEMS Microbiol. Lett. 184, 193–198. (10.1111/j.1574-6968.2000.tb09013.x) 10713420

[68] Kataeva IA, Seidel RD, Shah A, West LT, Li X-L, Ljungdahl LG 2002 The fibronectin type 3-like repeat from the *Clostridium thermocellum* cellobiohydrolase CbhA promotes hydrolysis of cellulose by modifying its surface. Appl. Environ. Microbiol. 68, 4292–4300. (10.1128/AEM.68.9.4292-4300.2002) 12200278 PMC124122

[69] Kneidinger B, Graninger M, Adam G, Puchberger M, Kosma P, Zayni S, Messner P 2001 Identification of two GDP-6-deoxy-D-lyxo-4-hexulose reductases synthesizing GDP-D-rhamnose in *Aneurinibacillus thermoaerophilus* L420-91T. J. Biol. Chem. 276, 5577–5583. (10.1074/jbc.M010027200) 11096116

[70] Faure D 2002 The family-3 glycoside hydrolases: from housekeeping functions to host-microbe interactions. Appl. Environ. Microbiol. 68, 1485–1490. (10.1128/AEM.68.4.1485-1490.2002) 11916659 PMC123870

[71] Zhou J et al 2017 Distinctive molecular and biochemical characteristics of a glycoside hydrolase family 20 β-N-acetylglucosaminidase and salt tolerance. BMC Biotechnol. 17, 37 (10.1186/s12896-017-0358-1) 28399848 PMC5387316

[72] Schröder C, Blank S, Antranikian G. 2015 First glycoside hydrolase family 2 enzymes from *Thermus antranikianii* and *Thermus brockianus* with β-glucosidase activity. Front. Bioeng. Biotechnol. 3, 76 (10.3389/fbioe.2015.00076) 26090361 PMC4453472

[73] Dietl A-M, Amich J, Leal S, Beckmann N, Binder U, Beilhack A, Pearlman E, Haas H 2016 Histidine biosynthesis plays a crucial role in metal homeostasis and virulence of *Aspergillus fumigatus*. Virulence 7, 465–476. (10.1080/21505594.2016.1146848) 26854126 PMC4871644

[74] Imada K, Sato M, Tanaka N, Katsube Y, Matsuura Y, Oshima T 1991 Three-dimensional structure of a highly thermostable enzyme, 3-isopropylmalate dehydrogenase of *Thermus thermophilus* at 2.2 Å resolution. J. Mol. Biol. 222, 725–738. (10.1016/0022-2836(91)90508-4) 1748999

[75] Allard STM, Giraud M-F, Naismith JH 2001 Epimerases: structure, function and mechanism. Cellular Mol. Life Sci. 58, 1650–1665. (10.1007/PL00000803) 11706991 PMC11337284

[76] Brown GM 1970 Biosynthesis of pantothenic acid and coenzyme A. In Comprehensive biochemistry (eds Florkin M, Stotz EH), pp. 73–80. Amsterdam, The Netherlands: Elsevier.

[77] Roujeinikova A, Baldock C, Simon WJ, Gilroy J, Baker PJ, Stuitje AR, Rice DW, Slabas AR, Rafferty JB 2002 X-ray crystallographic studies on butyryl-ACP reveal flexibility of the structure around a putative acyl chain binding site. Structure 10, 825–835. (10.1016/S0969-2126(02)00775-X) 12057197

[78] Blair DE, Schüttelkopf AW, MacRae JI, van Aalten DMF 2005 Structure and metal-dependent mechanism of peptidoglycan deacetylase, a streptococcal virulence factor. Proc. Natl Acad. Sci. USA 102, 15 429–15 434. (10.1073/pnas.0504339102) 16221761 PMC1252587

[79] Iyer LM, Abhiman S, Burroughs AM, Aravind L 2009 Amidoligases with ATP-grasp, glutamine synthetase-like and acetyltransferase-like domains: synthesis of novel metabolites and peptide modifications of proteins. Mol. Biosyst. 5, 1636–1660. (10.1039/b917682a) 20023723 PMC3268129

[80] Zhang Y, Mühlen S, Oates CV, Pearson JS, Hartland EL 2016 Identification of a distinct substrate-binding domain in the bacterial cysteine methyltransferase effectors NleE and OspZ. J. Biol. Chem. 291, 20 149–20 162. (10.1074/jbc.M116.734079) PMC5025698 27445336

[81] Kavanagh KL, Jörnvall H, Persson B, Oppermann U 2008 Medium- and short-chain dehydrogenase/reductase gene and protein families: the SDR superfamily: functional and structural diversity within a family of metabolic and regulatory enzymes. Cell. Mol. Life Sci. 65, 3895–3906. (10.1007/s00018-008-8588-y) 19011750 PMC2792337

[82] Pumirat P, Vanaporn M, Pinweha P, Tandhavanant S, Korbsrisate S, Chantratita N 2014 The role of short-chain dehydrogenase/oxidoreductase, induced by salt stress, on host interaction of *B. pseudomallei*. BMC Microbiol. 14, 1 (10.1186/1471-2180-14-1) PMC3882111 24382268

[83] Wyckoff TJO, Raetz CRH 1999 The active site of *Escherichia coli* UDP-*N*-acetylglucosamine acyltransferase chemical modification and site-directed mutagenesis. J. Biol. Chem. 274, 27 047–27 055. (10.1074/jbc.274.38.27047) 10480918

[84] Karlsson C, Jörnvall H, Höög J-O 1995 Zinc binding of alcohol and sorbitol dehydrogenases. In Enzymology and molecular biology of carbonyl metabolism 5 (eds Weiner H, Holmes RS, Wermuth B), pp. 397–406. Berlin, Germany: Springer. 10.1007/978-1-4615-1965-2_47 7484403

[85] Beckham KSH et al 2014 The metabolic enzyme AdhE controls the virulence of *Escherichia coli* O157: H7. Mol. Microbiol. 93, 199–211. (10.1111/mmi.12651) 24846743 PMC4249723

[86] Luong TT, Kim EH, Bak JP, Nguyen CT, Choi S, Briles DE, Pyo S, Rhee DK 2015 Ethanol-induced alcohol dehydrogenase E (AdhE) potentiates pneumolysin in *Streptococcus pneumoniae*. Infect. Immun. 83, 108–119. (10.1128/IAI.02434-14) 25312953 PMC4288861

[87] Biundo A, Steinkellner G, Gruber K, Spreitzhofer T, Ribitsch D, Guebitz GM 2017 Engineering of the zinc-binding domain of an esterase from *Clostridium botulinum* towards increased activity on polyesters. Catal. Sci. Technol. 7, 1440–1447. (10.1039/c7cy00168a)

[88] Lun S, Bishai WR 2007 Characterization of a novel cell wall-anchored protein with carboxylesterase activity required for virulence in *Mycobacterium tuberculosis*. J. Biol. Chem. 281, 18 348–18 356. (10.1074/jbc.M700035200) 17428787

[89] Krummrei U, Bang R, Schmidtchen R, Brune K, Bang H 1995 Cyclophilin-A is a zinc-dependent DNA binding protein in macrophages. FEBS Lett. 371, 47–51. (10.1016/0014-5793(95)00815-Q) 7664883

[90] Roset MS, Fernández LG, DelVecchio VG, Briones G 2013 Intracellularly induced cyclophilins play an important role in stress adaptation and virulence of *Brucella abortus*. Infect. Immun. 81, 521–530. (10.1128/IAI.01125-12) 23230297 PMC3553818

[91] Ünal CM, Steinert M 2014 Microbial peptidyl-prolyl cis/trans isomerases (PPIases): virulence factors and potential alternative drug targets. Microbiol. Mol. Biol. Rev. 78, 544–571. (10.1128/MMBR.00015-14) 25184565 PMC4187684

[92] Riboldi-Tunnicliffe A, König B, Jessen S, Weiss MS, Rahfeld J, Hacker J, Fischer G, Hilgenfeld R 2001 Crystal structure of Mip, a prolylisomerase from *Legionella pneumophila*. Nat. Struct. Mol. Biol. 8, 779 (10.1038/nsb0901-779) 11524681

[93] Neckers L, Tatu U 2008 Molecular chaperones in pathogen virulence: emerging new targets for therapy. Cell Host Microbe 4, 519–527. (10.1016/j.chom.2008.10.011) 19064253 PMC2752846

[94] Linke K, Wolfram T, Bussemer J, Jakob U 2003 The roles of the two zinc binding sites in DnaJ. J. Biol. Chem. 278, 44 457–44 466. (10.1074/jbc.M307491200) 12941935

[95] Wei C, Jiang W, Zhao M, Ling J, Zeng X, Deng J, Jin D, Dow JM, Sun W 2016 A systematic analysis of the role of GGDEF-EAL domain proteins in virulence and motility in *Xanthomonas oryzae* pv. *oryzicola*. Sci. Rep. 6, 23769 (10.1038/srep23769) 27053282 PMC4823724

[96] Ryan RP, McCarthy Y, Andrade M, Farah CS, Armitage JP, Dow JM 2010 Cell-cell signal-dependent dynamic interactions between HD-GYP and GGDEF domain proteins mediate virulence in *Xanthomonas campestris*. Proc. Natl Acad. Sci. USA 107, 5989–5994. (10.1073/pnas.0912839107) 20231439 PMC2851925

[97] Gao R, Mack TR, Stock AM 2007 Bacterial response regulators: versatile regulatory strategies from common domains. Trends Biochem. Sci. 32, 225–234. (10.1016/j.tibs.2007.03.002) 17433693 PMC3655528

[98] Wang S 2012 Bacterial two-component systems: structures and signaling mechanisms. In Protein phosphorylation in human health (ed. Huang Cai), pp. 439–466. London, UK: IntechOpen.

[99] Tanaka KJ, Song S, Mason K, Pinkett HW 2017 Selective substrate uptake: the role of ATP-binding cassette (ABC) importers in pathogenesis. Biochim. Biophys. Acta Biomembr. 1860, 868–877. (10.1016/j.bbamem.2017.08.011) 28847505 PMC5807212

[100] Lee D-W, El Khoury Y, Francia F, Zambelli B, Ciurli S, Venturoli G, Hellwig P, Daldal F 2011 Zinc inhibition of bacterial cytochrome *bc*_1_ reveals the role of cytochrome *b* E295 in proton release at the Qo site. Biochemistry 50, 4263–4272. (10.1021/bi200230e) 21500804 PMC3187937

[101] Ekici S, Pawlik G, Lohmeyer E, Koch HG, Daldal F 2012 Biogenesis of cbb3-type cytochrome c oxidase in *Rhodobacter capsulatus*. Biochim. Biophys. Acta Bioenerget. 1817, 898–910. (10.1016/j.bbabio.2011.10.011) PMC3304015 22079199

[102] Chiang P, Sampaleanu LM, Ayers M, Pahuta M, Howell PL, Burrows LL 2008 Functional role of conserved residues in the characteristic secretion NTPase motifs of the *Pseudomonas aeruginosa* type IV pilus motor proteins PilB, PilT and PilU. Microbiology 154, 114–126. (10.1099/mic.0.2007/011320-0) 18174131

[103] Giaquinto L, Curmi PMG, Siddiqui KS, Poljak A, DeLong E, DasSarma S, Cavicchioli R 2007 Structure and function of cold shock proteins in archaea. J. Bacteriol. 189, 5738–5748. (10.1128/JB.00395-07) 17545280 PMC1951829

[104] Lombardi PM, Cole KE, Dowling DP, Christianson DW 2011 Structure, mechanism, and inhibition of histone deacetylases and related metalloenzymes. Curr. Opin. Struct. Biol. 21, 735–743. (10.1016/j.sbi.2011.08.004) 21872466 PMC3232309

[105] Brown NL, Stoyanov JV, Kidd SP, Hobman JL 2003 The MerR family of transcriptional regulators. FEMS Microbiol. Rev. 27, 145–163. (10.1016/S0168-6445(03)00051-2) 12829265

[106] Kazmierczak MJ, Wiedmann M, Boor KJ 2005 Alternative sigma factors and their roles in bacterial virulence. Microbiol. Mol. Biol. Rev. 69, 527–543. (10.1128/MMBR.69.4.527) 16339734 PMC1306804

[107] Kenney LJ 2002 Structure/function relationships in OmpR and other winged-helix transcription factors. Curr. Opin. Microbiol. 5, 135–141. (10.1016/S1369-5274(02)00310-7) 11934608

[108] Banecki B, Wawrzynow A, Puzewicz J, Georgopoulos C, Zylicz M 2001 Structure-function analysis of the zinc-binding region of the ClpX molecular chaperone. J. Biol. Chem. 276, 18 843–18 848. (10.1074/jbc.M007507200) 11278349

[109] Chandler JR et al 2012 Bactobolin resistance is conferred by mutations in the L2 ribosomal protein. mBio 3, e00499-12 (10.1128/mBio.00499-12) 23249812 PMC3529544

[110] Kornder JD 2002 Streptomycin revisited: molecular action in the microbial cell. Med. Hypotheses 58, 34–46. (10.1054/mehy.2001.1450) 11863397

[111] Reader JS, Ordoukhanian PT, Kim JC, De Crécy-Lagard V, Hwang I, Farrand S, Schimmel P. 2005 Virology: major biocontrol of plant tumors targets tRNA synthetase. Science 309, 1533 (10.1126/science.1116841) 16141066

[112] Hurdle JG, O'Neill AJ, Chopra I 2005 Prospects for aminoacyl-tRNA synthetase inhibitors as new antimicrobial agents. Antimicrob. Agents Chemother. 49, 4821–4833. (10.1128/AAC.49.12.4821-4833.2005) 16304142 PMC1315952

[113] Lu M, Li Z, Liang J, Wei Y, Rensing C, Wei G 2016 Zinc resistance mechanisms of P 1B-type ATPases in *Sinorhizobium meliloti* CCNWSX0020. Sci. Rep. 6, 29355 (10.1038/srep29355) 27378600 PMC4932525

[114] Fones H, Preston GM 2012 Reactive oxygen and oxidative stress tolerance in plant pathogenic *Pseudomonas*. FEMS Microbiol. Lett. 327, 1–8. (10.1111/j.1574-6968.2011.02449.x) 22092667

[115] Wang X-G, Olsen LR, Roderick SL 2002 Structure of the lac operon galactoside acetyltransferase. Structure 10, 581–588. (10.1016/S0969-2126(02)00741-4) 11937062

[116] Bolz NJ, Lenhart JS, Weindorf SC, Simmons LA 2012 Residues in the N-terminal domain of MutL required for mismatch repair in *Bacillus subtilis*. J. Bacteriol. 194, 5361–5367. (10.1128/JB.01142-12) 22843852 PMC3457209

[117] Chen YC, Li CL, Hsiao YY, Duh Y, Yuan HS 2014 Structure and function of TatD exonuclease in DNA repair. Nucleic Acids Res. 42, 10 776–10 785. (10.1093/nar/gku732) PMC4176340 25114049

[118] Croteau DL, DellaVecchia MJ, Wang H, Bienstock RJ, Melton MA, Van Houten B. 2006 The C-terminal zinc finger of UvrA does not bind DNA directly but regulates damage-specific DNA binding. J. Biol. Chem. 281, 26 370–26 381. (10.1074/jbc.M603093200) PMC2396232 16829526

[119] Massova I, Mobashery S 1998 Kinship and diversification of bacterial penicillin-binding proteins and β-lactamases. Antimicrob. Agents Chemother. 42, 1–17. (10.1128/AAC.42.1.1) 9449253 PMC105448

[120] Barbas JA, Díaz J, Rodríguez-Tébar A, Vázquez D 1986 Specific location of penicillin-binding proteins within the cell envelope of *Escherichia coli*. J. Bacteriol. 165, 269–275. (10.1128/jb.165.1.269-275.1986) 3510188 PMC214399

[121] Büttner K, Wenig K, Hopfner KP 2005 Structural framework for the mechanism of archaeal exosomes in RNA processing. Mol. Cell 20, 461–471. (10.1016/j.molcel.2005.10.018) 16285927

[122] Matos RG, Bárria C, Moreira RN, Barahona S, Domingues S, Arraiano CM 2014 The importance of proteins of the RNase II/RNB-family in pathogenic bacteria. Front. Cellular Infect. Microbiol. 4, 68 (10.3389/fcimb.2014.00068) 24918089 PMC4042491

[123] Frees D, Gerth U, Ingmer H 2014 Clp chaperones and proteases are central in stress survival, virulence and antibiotic resistance of *Staphylococcus aureus*. Int. J. Med. Microbiol. 304, 142–149. (10.1016/j.ijmm.2013.11.009) 24457183

[124] Jang HB et al 2012 RNA-Seq-based metatranscriptomic and microscopic investigation reveals novel metalloproteases of *Neobodo* sp. as potential virulence factors for soft tunic syndrome in *Halocynthia roretzi*. PLoS ONE 7, e52379 (10.1371/journal.pone.0052379) 23300657 PMC3531462

[125] Agúndez L, MacHón C, Elvira César C, Rosa-Garrido M, Delgado MD, Llosa M 2011 Nuclear targeting of a bacterial integrase that mediates site-specific recombination between bacterial and human target sequences. Appl. Environ. Microbiol. 77, 201–210. (10.1128/AEM.01371-10) 21037296 PMC3019720

[126] Andreini C, Banci L, Bertini I, Rosato A 2006 Zinc through the three domains of life. J. Proteome Res. 5, 3173–3178. (10.1021/pr0603699) 17081069

[127] Kochańczyk T, Drozd A, Krężel A 2015 Relationship between the architecture of zinc coordination and zinc binding affinity in proteins–insights into zinc regulation. Metallomics 7, 244–257. (10.1039/C4MT00094C) 25255078

[128] Maret W 2013 Zinc biochemistry: from a single zinc enzyme to a key element of life. Adv. Nutrit. Int. Rev. J. 4, 82–91. (10.3945/an.112.003038) PMC3648744 23319127

[129] Coleman JE 1992 Zinc proteins: enzymes, storage proteins, transcription factors, and replication proteins. Annu. Rev. Biochem. 61, 897–946. (10.1146/annurev.bi.61.070192.004341) 1497326

[130] Kropachev KY, Zharkov DO, Grollman AP 2006 Catalytic mechanism of *Escherichia coli* endonuclease VIII: roles of the intercalation loop and the zinc finger. Biochemistry 45, 12 039–12 049. (10.1021/bi060663e) PMC2542946 17002303

[131] Ortiz de Orué Lucana D, Wedderhoff I, Groves MR 2012 ROS-mediated signalling in bacteria: zinc-containing Cys-XX-Cys redox centres and iron-based oxidative stress. J. Signal Transd. 2012, 1–9. (10.1155/2012/605905) PMC3184428 21977318

[132] Meini M-R, González LJ, Vila AJ 2013 Antibiotic resistance in Zn (II)-deficient environments: metallo-β-lactamase activation in the periplasm. Future Microbiol. 8, 947–979. (10.2217/fmb.13.34) 23902139 PMC3943169

[133] Hensley MP, Tierney DL, Crowder MW 2011 Zn (II) binding to *Escherichia coli* 70S ribosomes. Biochemistry 50, 9937–9939. (10.1021/bi200619w) 22026583 PMC3220600

[134] Scrutton MC, Wu CW, Goldthwait DA 1971 The presence and possible role of zinc in RNA polymerase obtained from *Escherichia coli*. Proc. Natl Acad. Sci. USA 68, 2497–2501. (10.1073/pnas.68.10.2497) 4944629 PMC389452

[135] Miller WT, Hill KAW, Schimmel P 1991 Evidence for a ‘cysteine-histidine box’ metal-binding site in an *Escherichia coli* aminoacyl-tRNA synthetase. Biochemistry 30, 6970–6976. (10.1021/bi00242a023) 1712632

[136] Campbell EA, Greenwell R, Anthony JR, Wang S, Lim L, Das K, Sofia HJ, Donohue TJ, Darst SA 2007 A conserved structural module regulates transcriptional responses to diverse stress signals in bacteria. Mol. Cell 27, 793–805. (10.1016/j.molcel.2007.07.009) 17803943 PMC2390684

[137] Chivers PT 2007 A galvanizing story—protein stability and zinc homeostasis. J. Bacteriol. 189, 2953–2954. (10.1128/JB.00173-07) 17307845 PMC1855864

[138] Rawlings ND, Barrett AJ 1995 Evolutionary families of metallopeptidases. Methods Enzymol. 248, 183–228. (10.1016/0076-6879(95)48015-3) 7674922

[139] Dönnes P, Höglund A 2004 Predicting protein subcellular localization: past, present, and future. Genomics Proteomics Bioinform. Beijing Genomics Inst. 2, 209–215. (10.1016/S1672-0229(04)02027-3) PMC5187447 15901249

[140] Ma L, Terwilliger A, Maresso AW 2015 Iron and zinc exploitation during bacterial pathogenesis. Metallomics 7, 1541–1554. (10.1039/C5MT00170F) 26497057 PMC4836889

[141] Cerasi M, Ammendola S, Battistoni A 2013 Competition for zinc binding in the host-pathogen interaction. Front. Cellular Infect. Microbiol. 3, 108 (10.3389/fcimb.2013.00108) 24400228 PMC3872050

[142] Zhu H, Liu M, Sumby P, Lei B 2009 The secreted esterase of group A *Streptococcus* is important for invasive skin infection and dissemination in mice. Infect. Immun. 77, 5225–5232. (10.1128/IAI.00636-09) 19805529 PMC2786455

[143] Kim JS, Park SJ, Kwak KJ, Kim YO, Kim JY, Song J, Jang B, Jung C-H, Kang H 2006 Cold shock domain proteins and glycine-rich RNA-binding proteins from *Arabidopsis thaliana* can promote the cold adaptation process in *Escherichia coli**.* Nucleic Acids Res. 35, 506–516. (10.1093/nar/gkl1076) 17169986 PMC1802614

[144] Chongdar N, Dasgupta S, Datta AB, Basu G 2015 Dispensability of zinc and the putative zinc-binding domain in bacterial glutamyl-tRNA synthetase. Biosci. Rep. 35, 1–13. (10.1042/BSR20150005) PMC4381286 25686371

