# Supplementary material for: Zinc binding proteome of a phytopathogen Xanthomonas translucens pv. undulosa
Source: R Soc Open Sci. 2019 Sep 25;6(9):190369. doi: 10.1098/rsos.190369 (PMC6774946; doi:10.1098/rsos.190369)
Supplement: Supplementary tables S1-S7 [file rsos190369supp1.docx]

**Supplementary material, table S1-S7**

**Supplementary material, table S1: Putative Zn-binding proteins of *Xtu* modeled by Phyre2**

| **S. No.** | **Sequence Id of putative Zn-binding protein** | **Template on which putative Zn-binding protein was modeled** | **Confidence of the modeled protein** | **Query Coverage of the modeled protein** | **Identity of the modeled protein to the template** | | **Description of the template on which putative Zn-binding protein was modeled** |
| --- | --- | --- | --- | --- | --- | --- | --- |
| 1 | WP_003465195.1 | c1ww8A | 100 | 54 | 44 | PDB header:oxidoreductase Chain: A: PDB Molecule:malate oxidoreductase; PDBTitle: crystal structure of malic enzyme from pyrococcus2 horikoshii ot3 | |
| 2 | WP_003465234.1 | c4s05B | 100 | 92 | 39 | PDB header:transcription/dna Chain: B: PDB Molecule:dna-binding transcriptional regulator basr; PDBTitle: crystal structure of klebsiella pneumoniae pmra in complex with pmra2 box dna | |
| 3 | WP_003465242.1 | c3ezlA | 100 | 98 | 64 | PDB header:oxidoreductase Chain: A: PDB Molecule:acetoacetyl-coa reductase; PDBTitle: crystal structure of acetyacetyl-coa reductase from2 burkholderia pseudomallei 1710b | |
| 4 | WP_003465267.1 | c4cvqB | 100 | 94 | 42 | PDB header:transferase Chain: B: PDB Molecule:glutamate-pyruvate aminotransferase alaa; PDBTitle: crystal structure of an aminotransferase from escherichia coli at 2.2 11 angstroem resolution | |
| 5 | WP_003465369.1 | c3u48A | 100 | 99 | 41 | PDB header:hydrolase Chain: A: PDB Molecule:jmb19063; PDBTitle: from soil to structure: a novel dimeric family 3-beta-glucosidase2 isolated from compost using metagenomic analysis | |
| 6 | WP_003465432.1 | c3dxbE | 100 | 95 | 60 | PDB header:splicing, transcription Chain: E: PDB Molecule:thioredoxin n-terminally fused to puf60(uhm); PDBTitle: structure of the uhm domain of puf60 fused to thioredoxin | |
| 7 | WP_003465445.1 | c4fc6B | 100 | 99 | 31 | PDB header:oxidoreductase Chain: B: PDB Molecule:peroxisomal 2,4-dienoyl-coa reductase; PDBTitle: studies on dcr shed new light on peroxisomal beta-oxidation: crystal2 structure of the ternary complex of pdcr | |
| 8 | WP_003465455.1 | c5briA | 100 | 95 | 51 | PDB header:signaling protein Chain: A: PDB Molecule:candidate response regulator, chey; PDBTitle: bacteriophytochrome response regulator rtbrr | |
| 9 | WP_003465502.1 | c2vdaA | 100 | 90 | 63 | PDB header:protein transport Chain: A: PDB Molecule:translocase subunit seca; PDBTitle: solution structure of the seca-signal peptide complex | |
| 10 | WP_003465522.1 | c3m6nA | 100 | 92 | 70 | PDB header:lyase Chain: A: PDB Molecule:rpff protein; PDBTitle: crystal structure of rpff | |
| 11 | WP_003465615.1 | c3q9lB | 100 | 94 | 69 | PDB header:cell cycle, hydrolase Chain: B: PDB Molecule:septum site-determining protein mind; PDBTitle: the structure of the dimeric e.coli mind-atp complex | |
| 12 | WP_003465620.1 | c5hevC | 100 | 99 | 43 | PDB header:transcription Chain: C: PDB Molecule:response regulator protein vrar; PDBTitle: crystal structure of the beryllofluoride-activated liar from2 enterococcus faecium | |
| 13 | WP_003465625.1 | c3fwlA | 100 | 90 | 36 | PDB header:transferase, hydrolase Chain: A: PDB Molecule:penicillin-binding protein 1b; PDBTitle: crystal structure of the full-length transglycosylase pbp1b2 from escherichia coli | |
| 14 | WP_003465697.1 | d2pw6a1 | 100 | 93 | 36 | Fold:Phosphorylase/hydrolase-like Superfamily:LigB-like Family:LigB-like | |
| 15 | WP_003465707.1 | c3v0tA | 100 | 96 | 52 | PDB header:oxidoreductase Chain: A: PDB Molecule:perakine reductase; PDBTitle: crystal structure of perakine reductase, founder member of a novel akr2 subfamily with unique conformational changes during nadph binding | |
| 16 | WP_003465766.1 | c2zunB | 100 | 73 | 49 | PDB header:hydrolase Chain: B: PDB Molecule:458aa long hypothetical endo-1,4-beta-glucanase; PDBTitle: functional analysis of hyperthermophilic endocellulase from2 the archaeon pyrococcus horikoshii | |
| 17 | WP_003465837.1 | c4czeA | 100 | 93 | 64 | PDB header:structural protein Chain: A: PDB Molecule:rod shape-determining protein mreb; PDBTitle: c. crescentus mreb, double filament, empty | |
| 18 | WP_003465861.1 | c2nx8A | 100 | 93 | 29 | PDB header:hydrolase Chain: A: PDB Molecule:trna-specific adenosine deaminase; PDBTitle: the crystal structure of the trna-specific adenosine deaminase from2 streptococcus pyogenes | |
| 19 | WP_003465899.1 | c3h14A | 100 | 94 | 43 | PDB header:transferase Chain: A: PDB Molecule:aminotransferase, classes i and ii; PDBTitle: crystal structure of a putative aminotransferase from silicibacter2 pomeroyi | |
| 20 | WP_003465966.1 | c1rrqA | 100 | 98 | 35 | PDB header:hydrolase/dna Chain: A: PDB Molecule:muty; PDBTitle: muty adenine glycosylase in complex with dna containing an2 a:oxog pair | |
| 21 | WP_003465967.1 | c2qy9A | 100 | 68 | 55 | PDB header:protein transport Chain: A: PDB Molecule:cell division protein ftsy; PDBTitle: structure of the ng+1 construct of the e. coli srp receptor2 ftsy | |
| 22 | WP_003465985.1 | c4q1vA | 100 | 94 | 21 | PDB header:hydrolase Chain: A: PDB Molecule:putative dipeptidyl aminopeptidase iv; PDBTitle: crystal structure of a putative dipeptidyl aminopeptidase iv2 (bacova_01349) from bacteroides ovatus atcc 8483 at 2.48 a resolution | |
| 23 | WP_003466074.1 | c2qflA | 100 | 95 | 44 | PDB header:hydrolase Chain: A: PDB Molecule:inositol-1-monophosphatase; PDBTitle: structure of suhb: inositol monophosphatase and extragenic2 suppressor from e. coli | |
| 24 | WP_003466157.1 | c1ys7B | 100 | 92 | 36 | PDB header:transcription regulator Chain: B: PDB Molecule:transcriptional regulatory protein prra; PDBTitle: crystal structure of the response regulator protein prra complexed2 with mg2+ | |
| 25 | WP_003466231.1 | c1zpuE | 100 | 91 | 31 | PDB header:oxidoreductase Chain: E: PDB Molecule:iron transport multicopper oxidase fet3; PDBTitle: crystal structure of fet3p, a multicopper oxidase that functions in2 iron impor | |
| 26 | WP_003466285.1 | c1q1bD | 100 | 96 | 30 | PDB header:transport protein Chain: D: PDB Molecule:maltose/maltodextrin transport atp-binding protein malk; PDBTitle: crystal structure of e. coli malk in the nucleotide-free form | |
| 27 | WP_003466345.1 | c4tqvO | 100 | 95 | 39 | PDB header:transport protein Chain: O: PDB Molecule:algs; PDBTitle: crystal structure of a bacterial abc transporter involved in the2 import of the acidic polysaccharide alginate | |
| 28 | WP_003466372.1 | c2nu8D | 100 | 98 | 71 | PDB header:ligase Chain: D: PDB Molecule:succinyl-coa ligase [adp-forming] subunit alpha; PDBTitle: c123at mutant of e. coli succinyl-coa synthetase | |
| 29 | WP_003466374.1 | c2nu9E | 100 | 99 | 60 | PDB header:ligase Chain: E: PDB Molecule:succinyl-coa synthetase beta chain; PDBTitle: c123at mutant of e. coli succinyl-coa synthetase2 orthorhombic crystal form | |
| 30 | WP_003466509.1 | c2q2qG | 100 | 99 | 41 | PDB header:oxidoreductase Chain: G: PDB Molecule:beta-d-hydroxybutyrate dehydrogenase; PDBTitle: structure of d-3-hydroxybutyrate dehydrogenase from pseudomonas putida | |
| 31 | WP_003466549.1 | c2gbzA | 100 | 92 | 84 | PDB header:hydrolase Chain: A: PDB Molecule:oligoribonuclease; PDBTitle: the crystal structure of xc847 from xanthomonas campestris: a 3-52 oligoribonuclease of dnaq fold family with a novel opposingly-shifted3 helix | |
| 32 | WP_003466552.1 | c2nx8A | 100 | 93 | 35 | PDB header:hydrolase Chain: A: PDB Molecule:trna-specific adenosine deaminase; PDBTitle: the crystal structure of the trna-specific adenosine deaminase from2 streptococcus pyogenes | |
| 33 | WP_003466555.1 | c2h09A | 100 | 81 | 69 | PDB header:transcription Chain: A: PDB Molecule:transcriptional regulator mntr; PDBTitle: crystal structure of diphtheria toxin repressor like protein2 from e. coli | |
| 34 | WP_003466655.1 | c3pcoC | 100 | 97 | 64 | PDB header:ligase Chain: C: PDB Molecule:phenylalanyl-trna synthetase, alpha subunit; PDBTitle: crystal structure of e. coli phenylalanine-trna synthetase complexed2 with phenylalanine and amp | |
| 35 | WP_003466735.1 | c5fl3A | 100 | 91 | 45 | PDB header:transport protein Chain: A: PDB Molecule:pili retraction protein pilt; PDBTitle: pilt2 from thermus thermophilus | |
| 36 | WP_003466789.1 | c4egbC | 100 | 95 | 52 | PDB header:lyase Chain: C: PDB Molecule:dtdp-glucose 4,6-dehydratase; PDBTitle: 3.0 angstrom resolution crystal structure of dtdp-glucose 4,6-2 dehydratase (rfbb) from bacillus anthracis str. ames in complex with3 nad | |
| 37 | WP_003466792.1 | c3sc6F | 100 | 97 | 35 | PDB header:oxidoreductase Chain: F: PDB Molecule:dtdp-4-dehydrorhamnose reductase; PDBTitle: 2.65 angstrom resolution crystal structure of dtdp-4-dehydrorhamnose2 reductase (rfbd) from bacillus anthracis str. ames in complex with3 nadp | |
| 38 | WP_003466794.1 | c5bmpA | 100 | 99 | 89 | PDB header:isomerase Chain: A: PDB Molecule:phosphoglucomutase; PDBTitle: crystal structure of phosphoglucomutase from xanthomonas citri2 complexed with glucose-1-phosphate | |
| 39 | WP_003466826.1 | c3jruB | 100 | 97 | 84 | PDB header:hydrolase Chain: B: PDB Molecule:probable cytosol aminopeptidase; PDBTitle: crystal structure of leucyl aminopeptidase (pepa) from xoo0834,2 xanthomonas oryzae pv. oryzae kacc10331 | |
| 40 | WP_003466920.1 | c1zvvA | 100 | 94 | 25 | PDB header:transcription/dna Chain: A: PDB Molecule:glucose-resistance amylase regulator; PDBTitle: crystal structure of a ccpa-crh-dna complex | |
| 41 | WP_003466936.1 | c2qmiH | 100 | 75 | 26 | PDB header:hydrolase Chain: H: PDB Molecule:pbp related beta-lactamase; PDBTitle: structure of the octameric penicillin-binding protein2 homologue from pyrococcus abyssi | |
| 42 | WP_003466950.1 | c3a4hA | 100 | 99 | 41 | PDB header:oxidoreductase Chain: A: PDB Molecule:vitamin d hydroxylase; PDBTitle: structure of cytochrome p450 vdh from pseudonocardia autotrophica2 (orthorhombic crystal form) | |
| 43 | WP_003466959.1 | c3tzqD | 100 | 90 | 41 | PDB header:oxidoreductase Chain: D: PDB Molecule:short-chain type dehydrogenase/reductase; PDBTitle: crystal structure of a short-chain type dehydrogenase/reductase from2 mycobacterium marinum | |
| 44 | WP_003466981.1 | c3vr1B | 100 | 98 | 61 | PDB header:translation Chain: B: PDB Molecule:peptide chain release factor 3; PDBTitle: crystal structure analysis of the translation factor rf3 | |
| 45 | WP_003467001.1 | c4zrmB | 100 | 97 | 30 | PDB header:isomerase Chain: B: PDB Molecule:udp-glucose 4-epimerase; PDBTitle: crystal structure of udp-glucose 4-epimerase (tm0509) from2 hyperthermophilic eubacterium thermotoga maritima | |
| 46 | WP_003467030.1 | c1cmwA | 100 | 99 | 43 | PDB header:transferase Chain: A: PDB Molecule:protein (dna polymerase i); PDBTitle: crystal structure of taq dna-polymerase shows a new orientation for2 the structure-specific nuclease domain | |
| 47 | WP_003467094.1 | c1rqgA | 100 | 91 | 31 | PDB header:ligase Chain: A: PDB Molecule:methionyl-trna synthetase; PDBTitle: methionyl-trna synthetase from pyrococcus abyssi | |
| 48 | WP_003467251.1 | c4ymuJ | 100 | 88 | 38 | PDB header:protein binding/transport protein Chain: J: PDB Molecule:abc-type polar amino acid transport system, atpase PDBTitle: crystal structure of an amino acid abc transporter complex with2 arginines and atps | |
| 49 | WP_003467261.1 | c5swcE | 100 | 92 | 41 | PDB header:lyase Chain: E: PDB Molecule:carbonic anhydrase; PDBTitle: the structure of the beta-carbonic anhydrase ccaa | |
| 50 | WP_003467331.1 | c3cm0A | 99.9 | 98 | 54 | PDB header:transferase Chain: A: PDB Molecule:adenylate kinase; PDBTitle: crystal structure of adenylate kinase from thermus2 thermophilus hb8 | |
| 51 | WP_003467341.1 | c3e03C | 100 | 99 | 84 | PDB header:oxidoreductase Chain: C: PDB Molecule:short chain dehydrogenase; PDBTitle: crystal structure of a putative dehydrogenase from2 xanthomonas campestris | |
| 52 | WP_003467345.1 | c4mtsA | 99.9 | 86 | 35 | PDB header:lyase Chain: A: PDB Molecule:lactoylglutathione lyase; PDBTitle: ni- and zn-bound gloa2 at high resolution | |
| 53 | WP_003467386.1 | c4nbqB | 100 | 97 | 61 | PDB header:transferase Chain: B: PDB Molecule:polyribonucleotide nucleotidyltransferase; PDBTitle: structure of the polynucleotide phosphorylase (cbu_0852) from coxiella2 burnetii | |
| 54 | WP_003467454.1 | c2f9yB | 100 | 88 | 67 | PDB header:ligase Chain: B: PDB Molecule:acetyl-coenzyme a carboxylase carboxyl transferase subunit PDBTitle: the crystal structure of the carboxyltransferase subunit of acc from2 escherichia coli | |
| 55 | WP_003467543.1 | c4h60A | 100 | 92 | 30 | PDB header:signaling protein Chain: A: PDB Molecule:chemotaxis protein chey; PDBTitle: high resolution structure of vibrio cholerae chemotaxis protein chey42 crystallized in low ph (4.0) condition | |
| 56 | WP_003467872.1 | c2okvC | 100 | 98 | 42 | PDB header:hydrolase Chain: C: PDB Molecule:probable d-tyrosyl-trna(tyr) deacylase 1; PDBTitle: c-myc dna unwinding element binding protein | |
| 57 | WP_003467936.1 | c3a0jB | 99.9 | 90 | 54 | PDB header:transcription Chain: B: PDB Molecule:cold shock protein; PDBTitle: crystal structure of cold shock protein 1 from thermus2 thermophilus hb8 | |
| 58 | WP_003467941.1 | c4d7aA | 100 | 92 | 47 | PDB header:ligase Chain: A: PDB Molecule:trna threonylcarbamoyladenosine dehydratase; PDBTitle: crystal structure of e. coli trna n6-threonylcarbamoyladenosine2 dehydratase, tcda, in complex with amp at 1.801 angstroem3 resolution | |
| 59 | WP_003467943.1 | c2gzxB | 100 | 95 | 33 | PDB header:structural genomics, unknown function Chain: B: PDB Molecule:putative tatd related dnase; PDBTitle: crystal structure of the tatd deoxyribonuclease mw0446 from2 staphylococcus aureus. northeast structural genomics consortium3 target zr237. | |
| 60 | WP_003467994.1 | c2w57A | 100 | 92 | 53 | PDB header:metal transport Chain: A: PDB Molecule:ferric uptake regulation protein; PDBTitle: crystal structure of the vibrio cholerae ferric uptake2 regulator (fur) reveals structural rearrangement of the3 dna-binding domains | |
| 61 | WP_003468012.1 | c2khoA | 100 | 94 | 74 | PDB header:chaperone Chain: A: PDB Molecule:heat shock protein 70; PDBTitle: nmr-rdc / xray structure of e. coli hsp70 (dnak) chaperone (1-605)2 complexed with adp and substrate | |
| 62 | WP_003468017.1 | c5b6aA | 100 | 93 | 32 | PDB header:transferase Chain: A: PDB Molecule:pyridoxal kinase pdxy; PDBTitle: structure of pyridoxal kinasefrom pseudomonas aeruginosa | |
| 63 | WP_003468030.1 | c5mkkB | 100 | 98 | 33 | PDB header:transport protein Chain: B: PDB Molecule:multidrug resistance abc transporter atp-binding and PDBTitle: crystal structure of the heterodimeric abc transporter tmrab, a2 homolog of the antigen translocation complex tap | |
| 64 | WP_003468120.1 | c3wj7B | 100 | 97 | 22 | PDB header:oxidoreductase Chain: B: PDB Molecule:putative oxidoreductase; PDBTitle: crystal structure of gox2253 | |
| 65 | WP_003468235.1 | c3i1jB | 100 | 97 | 41 | PDB header:oxidoreductase Chain: B: PDB Molecule:oxidoreductase, short chain dehydrogenase/reductase family; PDBTitle: structure of a putative short chain dehydrogenase from pseudomonas2 syringae | |
| 66 | WP_003468255.1 | c2d7dA | 100 | 98 | 58 | PDB header:hydrolase/dna Chain: A: PDB Molecule:uvrabc system protein b; PDBTitle: structural insights into the cryptic dna dependent atp-ase2 activity of uvrb | |
| 67 | WP_003468289.1 | c2dq3A | 100 | 99 | 51 | PDB header:ligase Chain: A: PDB Molecule:seryl-trna synthetase; PDBTitle: crystal structure of aq_298 | |
| 68 | WP_003468393.1 | c2f9iC | 100 | 97 | 54 | PDB header:transferase Chain: C: PDB Molecule:acetyl-coenzyme a carboxylase carboxyl PDBTitle: crystal structure of the carboxyltransferase subunit of acc2 from staphylococcus aureus | |
| 69 | WP_003468480.1 | c5jbxB | 100 | 99 | 35 | PDB header:lyase Chain: B: PDB Molecule:3-hydroxybutyryl-coa dehydratase; PDBTitle: crystal structure of liuc in complex with coenzyme a and malonic acid | |
| 70 | WP_003468516.1 | c1ys7B | 100 | 98 | 37 | PDB header:transcription regulator Chain: B: PDB Molecule:transcriptional regulatory protein prra; PDBTitle: crystal structure of the response regulator protein prra complexed2 with mg2+ | |
| 71 | WP_003468529.1 | c2d1cB | 100 | 99 | 49 | PDB header:oxidoreductase Chain: B: PDB Molecule:isocitrate dehydrogenase; PDBTitle: crystal structure of tt0538 protein from thermus thermophilus hb8 | |
| 72 | WP_003468538.1 | c3hteC | 100 | 81 | 80 | PDB header:motor protein Chain: C: PDB Molecule:atp-dependent clp protease atp-binding subunit clpx; PDBTitle: crystal structure of nucleotide-free hexameric clpx | |
| 73 | WP_003468546.1 | c4ysbB | 100 | 96 | 34 | PDB header:hydrolase Chain: B: PDB Molecule:metallo-beta-lactamase family protein; PDBTitle: crystal structure of ethe1 from myxococcus xanthus | |
| 74 | WP_003468608.1 | c3oj7A | 100 | 97 | 44 | PDB header:metal binding protein Chain: A: PDB Molecule:putative histidine triad family protein; PDBTitle: crystal structure of a histidine triad family protein from entamoeba2 histolytica, bound to sulfate | |
| 75 | WP_003468623.1 | c1zfnA | 100 | 65 | 41 | PDB header:transferase Chain: A: PDB Molecule:adenylyltransferase thif; PDBTitle: structural analysis of escherichia coli thif | |
| 76 | WP_003468634.1 | c3j8gX | 100 | 93 | 50 | PDB header:ribosome Chain: X: PDB Molecule:gtpase der; PDBTitle: electron cryo-microscopy structure of enga bound with the 50s2 ribosomal subunit | |
| 77 | WP_003468654.1 | c3mogA | 100 | 53 | 23 | PDB header:oxidoreductase Chain: A: PDB Molecule:probable 3-hydroxybutyryl-coa dehydrogenase; PDBTitle: crystal structure of 3-hydroxybutyryl-coa dehydrogenase from2 escherichia coli k12 substr. mg1655 | |
| 78 | WP_003468695.1 | c5lo9B | 99.8 | 56 | 33 | PDB header:oxidoreductase Chain: B: PDB Molecule:cytochrome c; PDBTitle: thiosulfate dehydrogenase (tsdba) from marichromatium purpuratum - "as2 isolated" form | |
| 79 | WP_003468804.1 | c4m8sB | 100 | 99 | 71 | PDB header:oxidoreductase Chain: B: PDB Molecule:putative 3-oxoacyl-[acyl-carrier protein] reductase; PDBTitle: crystal structure of 3-ketoacyl -(acyl carrier protein) reductase2 (fabg) from neisseria meningitidis | |
| 80 | WP_003468806.1 | c2m5rA | 99.8 | 98 | 41 | PDB header:lipid binding protein Chain: A: PDB Molecule:acyl carrier protein; PDBTitle: solution structure of holo-acyl carrier protein of leishmania major | |
| 81 | WP_003468858.1 | c5hjhB | 100 | 86 | 27 | PDB header:metal binding protein Chain: B: PDB Molecule:ferritin, dps family protein; PDBTitle: dps4 from nostoc punctiforme in complex with fe ions | |
| 82 | WP_003468914.1 | c3ux8A | 100 | 94 | 71 | PDB header:dna binding protein Chain: A: PDB Molecule:excinuclease abc, a subunit; PDBTitle: crystal structure of uvra | |
| 83 | WP_003469000.1 | c4k2xB | 100 | 99 | 29 | PDB header:oxidoreductase, flavoprotein Chain: B: PDB Molecule:polyketide oxygenase/hydroxylase; PDBTitle: oxys anhydrotetracycline hydroxylase from streptomyces rimosus | |
| 84 | WP_003469028.1 | c5jydA | 100 | 98 | 48 | PDB header:oxidoreductase Chain: A: PDB Molecule:short chain dehydrogenase; PDBTitle: crystal structure of a putative short chain dehydrogenase from2 burkholderia cenocepacia | |
| 85 | WP_003469157.1 | c3j6vL | 100 | 87 | 48 | PDB header:ribosome Chain: L: PDB Molecule:28s ribosomal protein s12, mitochondrial; PDBTitle: cryo-em structure of the small subunit of the mammalian mitochondrial2 ribosome | |
| 86 | WP_003469161.1 | c2rdo7 | 100 | 99 | 70 | PDB header:ribosome Chain: 7: PDB Molecule:elongation factor g; PDBTitle: 50s subunit with ef-g(gdpnp) and rrf bound | |
| 87 | WP_003469170.1 | c4afnB | 100 | 97 | 40 | PDB header:oxidoreductase Chain: B: PDB Molecule:3-oxoacyl-[acyl-carrier-protein] reductase fabg; PDBTitle: crystal structure of 3-ketoacyl-(acyl-carrier-protein) reductase2 (fabg) from pseudomonas aeruginosa at 2.3a resolution | |
| 88 | WP_003469200.1 | c2gwrA | 100 | 96 | 31 | PDB header:signaling protein Chain: A: PDB Molecule:dna-binding response regulator mtra; PDBTitle: crystal structure of the response regulator protein mtra from2 mycobacterium tuberculosis | |
| 89 | WP_003469256.1 | c2pptA | 99.9 | 97 | 25 | PDB header:oxidoreductase Chain: A: PDB Molecule:thioredoxin-2; PDBTitle: crystal structure of thioredoxin-2 | |
| 90 | WP_003469280.1 | c1ys7B | 100 | 97 | 41 | PDB header:transcription regulator Chain: B: PDB Molecule:transcriptional regulatory protein prra; PDBTitle: crystal structure of the response regulator protein prra complexed2 with mg2+ | |
| 91 | WP_003469507.1 | c1s7cA | 100 | 99 | 73 | PDB header:structural genomics, oxidoreductase Chain: A: PDB Molecule:glyceraldehyde 3-phosphate dehydrogenase a; PDBTitle: crystal structure of mes buffer bound form of glyceraldehyde 3-2 phosphate dehydrogenase from escherichia coli | |
| 92 | WP_003469512.1 | c2hhgA | 100 | 95 | 34 | PDB header:structural genomics, unknown function Chain: A: PDB Molecule:hypothetical protein rpa3614; PDBTitle: structure of protein of unknown function rpa3614, possible tyrosine2 phosphatase, from rhodopseudomonas palustris cga009 | |
| 93 | WP_003469598.1 | c6an0A | 100 | 99 | 47 | PDB header:oxidoreductase Chain: A: PDB Molecule:histidinol dehydrogenase; PDBTitle: crystal structure of histidinol dehydrogenase from elizabethkingia2 anophelis | |
| 94 | WP_003469599.1 | c3eucB | 100 | 96 | 30 | PDB header:transferase Chain: B: PDB Molecule:histidinol-phosphate aminotransferase 2; PDBTitle: crystal structure of histidinol-phosphate aminotransferase2 (yp_297314.1) from ralstonia eutropha jmp134 at 2.05 a resolution | |
| 95 | WP_003469626.1 | c1qu2A | 100 | 98 | 43 | PDB header:ligase/rna Chain: A: PDB Molecule:isoleucyl-trna synthetase; PDBTitle: insights into editing from an ile-trna synthetase structure2 with trna(ile) and mupirocin | |
| 96 | WP_003469645.1 | c2yevA | 100 | 93 | 27 | PDB header:electron transport Chain: A: PDB Molecule:cytochrome c oxidase polypeptide i+iii; PDBTitle: structure of caa3-type cytochrome oxidase | |
| 97 | WP_003469868.1 | c4pmyB | 100 | 92 | 67 | PDB header:hydrolase Chain: B: PDB Molecule:xylanase; PDBTitle: crystal structure of gh10 endo-b-1,4-xylanase (xynb) from xanthomonas2 axonopodis pv citri complexed with xylose | |
| 98 | WP_003469871.1 | c4pmuD | 100 | 88 | 82 | PDB header:hydrolase Chain: D: PDB Molecule:endo-1,4-beta-xylanase a; PDBTitle: crystal structure of a novel reducing-end xylose-releasing exo-2 oligoxylanase (xyna) belonging to gh10 family (space group p1211) | |
| 99 | WP_003469968.1 | c3gehA | 100 | 99 | 39 | PDB header:hydrolase Chain: A: PDB Molecule:trna modification gtpase mnme; PDBTitle: crystal structure of mnme from nostoc in complex with gdp, folinic2 acid and zn | |
| 100 | WP_003469990.1 | c4i3hA | 100 | 95 | 52 | PDB header:isomerase/dna Chain: A: PDB Molecule:topoisomerase iv subunit b, dna topoisomerase 4 subunit a PDBTitle: a three-gate structure of topoisomerase iv from streptococcus2 pneumoniae | |
| 101 | WP_003470030.1 | c2gq1A | 100 | 97 | 43 | PDB header:hydrolase Chain: A: PDB Molecule:fructose-1,6-bisphosphatase; PDBTitle: crystal structure of recombinant type i fructose-1,6-bisphosphatase2 from escherichia coli complexed with sulfate ions | |
| 102 | WP_003470036.1 | c1lluD | 100 | 99 | 71 | PDB header:oxidoreductase Chain: D: PDB Molecule:alcohol dehydrogenase; PDBTitle: the ternary complex of pseudomonas aeruginosa alcohol2 dehydrogenase with its coenzyme and weak substrate | |
| 103 | WP_003470124.1 | c1u0bB | 100 | 99 | 46 | PDB header:ligase/rna Chain: B: PDB Molecule:cysteinyl trna; PDBTitle: crystal structure of cysteinyl-trna synthetase binary2 complex with trnacys | |
| 104 | WP_003470165.1 | c4xkmB | 100 | 97 | 62 | PDB header:isomerase Chain: B: PDB Molecule:xylose isomerase; PDBTitle: crystal structure of xylose isomerase from an human intestinal tract2 microbe bacteroides thetaiotaomicron | |
| 105 | WP_003470173.1 | c3kwcD | 100 | 95 | 32 | PDB header:lyase, protein binding, photosynthesis Chain: D: PDB Molecule:carbon dioxide concentrating mechanism protein; PDBTitle: oxidized, active structure of the beta-carboxysomal gamma-carbonic2 anhydrase, ccmm | |
| 106 | WP_003470192.1 | c5j1jA | 100 | 90 | 55 | PDB header:transcription Chain: A: PDB Molecule:site-determining protein; PDBTitle: structure of flen-amppnp complex | |
| 107 | WP_003470254.1 | c4am4B | 100 | 99 | 51 | PDB header:metal binding protein Chain: B: PDB Molecule:bacterioferritin; PDBTitle: bacterioferritin from blastochloris viridis | |
| 108 | WP_003470294.1 | c1j9zB | 100 | 89 | 33 | PDB header:oxidoreductase Chain: B: PDB Molecule:nadph-cytochrome p450 reductase; PDBTitle: cypor-w677g | |
| 109 | WP_003470297.1 | c3agjC | 100 | 61 | 33 | PDB header:translation/hydrolase Chain: C: PDB Molecule:elongation factor 1-alpha; PDBTitle: crystal structure of archaeal pelota and gtp-bound ef1 alpha complex | |
| 110 | WP_003470359.1 | c2nwqA | 100 | 97 | 58 | PDB header:oxidoreductase Chain: A: PDB Molecule:probable short-chain dehydrogenase; PDBTitle: short chain dehydrogenase from pseudomonas aeruginosa | |
| 111 | WP_003470405.1 | c4rulA | 100 | 94 | 38 | PDB header:isomerase/dna Chain: A: PDB Molecule:dna topoisomerase 1; PDBTitle: crystal structure of full-length e.coli topoisomerase i in complex2 with ssdna | |
| 112 | WP_003470459.1 | c1xnwD | 100 | 99 | 28 | PDB header:ligase Chain: D: PDB Molecule:propionyl-coa carboxylase complex b subunit; PDBTitle: acyl-coa carboxylase beta subunit from s. coelicolor (pccb),2 apo form #2, mutant d422i | |
| 113 | WP_003470478.1 | c4do7B | 100 | 95 | 35 | PDB header:hydrolase Chain: B: PDB Molecule:amidohydrolase 2; PDBTitle: crystal structure of an amidohydrolase (cog3618) from burkholderia2 multivorans (target efi-500235) with bound zn, space group c2 | |
| 114 | WP_003470527.1 | c1pzsA | 100 | 67 | 37 | PDB header:oxidoreductase, metal binding protein Chain: A: PDB Molecule:superoxide dismutase [cu-zn]; PDBTitle: crystal structure of a cu-zn superoxide dismutase from mycobacterium2 tuberculosis at 1.63 resolution | |
| 115 | WP_003470587.1 | c4xk2A | 100 | 99 | 74 | PDB header:oxidoreductase Chain: A: PDB Molecule:aldo/keto reductase; PDBTitle: crystal structure of aldo-keto reductase from polaromonas sp. Js666 | |
| 116 | WP_003470612.1 | c1b8vA | 100 | 99 | 78 | PDB header:oxidoreductase Chain: A: PDB Molecule:protein (malate dehydrogenase); PDBTitle: malate dehydrogenase from aquaspirillum arcticum | |
| 117 | WP_003470615.1 | c1ihgA | 100 | 93 | 42 | PDB header:isomerase Chain: A: PDB Molecule:cyclophilin 40; PDBTitle: bovine cyclophilin 40, monoclinic form | |
| 118 | WP_003470617.1 | c4zciA | 100 | 99 | 70 | PDB header:gtp-binding protein Chain: A: PDB Molecule:gtp-binding protein typa/bipa; PDBTitle: crystal structure of escherichia coli gtpase bipa/typa | |
| 119 | WP_003470629.1 | c3iydA | 100 | 97 | 65 | PDB header:transcription/dna Chain: A: PDB Molecule:dna-directed rna polymerase subunit alpha; PDBTitle: three-dimensional em structure of an intact activator-dependent2 transcription initiation complex | |
| 120 | WP_003470630.1 | c5o5jD | 100 | 99 | 48 | PDB header:ribosome Chain: D: PDB Molecule:30s ribosomal protein s4; PDBTitle: structure of the 30s small ribosomal subunit from mycobacterium2 smegmatis | |
| 121 | WP_003470641.1 | c5o5jE | 100 | 86 | 53 | PDB header:ribosome Chain: E: PDB Molecule:30s ribosomal protein s5; PDBTitle: structure of the 30s small ribosomal subunit from mycobacterium2 smegmatis | |
| 122 | WP_003470646.1 | c5o5jH | 100 | 97 | 53 | PDB header:ribosome Chain: H: PDB Molecule:30s ribosomal protein s8; PDBTitle: structure of the 30s small ribosomal subunit from mycobacterium2 smegmatis | |
| 123 | WP_003470648.1 | c3bbnN | 100 | 98 | 40 | PDB header:ribosome Chain: N: PDB Molecule:ribosomal protein s14; PDBTitle: homology model for the spinach chloroplast 30s subunit fitted to 9.4a2 cryo-em map of the 70s chlororibosome. | |
| 124 | WP_003470669.1 | c3j3wC | 100 | 99 | 56 | PDB header:ribosome Chain: C: PDB Molecule:50s ribosomal protein l2; PDBTitle: atomic model of the immature 50s subunit from bacillus subtilis (state2 ii-a) | |
| 125 | WP_003470782.1 | c4s05B | 100 | 95 | 45 | PDB header:transcription/dna Chain: B: PDB Molecule:dna-binding transcriptional regulator basr; PDBTitle: crystal structure of klebsiella pneumoniae pmra in complex with pmra2 box dna | |
| 126 | WP_003470870.1 | c3ut2B | 100 | 99 | 61 | PDB header:oxidoreductase Chain: B: PDB Molecule:catalase-peroxidase 2; PDBTitle: crystal structure of fungal magkatg2 | |
| 127 | WP_003470923.1 | c1xxiF | 100 | 98 | 29 | PDB header:transferase Chain: F: PDB Molecule:dna polymerase iii, delta subunit; PDBTitle: adp bound e. coli clamp loader complex | |
| 128 | WP_003470942.1 | c3sy8C | 100 | 85 | 20 | PDB header:transcription regulator Chain: C: PDB Molecule:rocr; PDBTitle: crystal structure of the response regulator rocr | |
| 129 | WP_003471107.1 | c2ecfA | 100 | 87 | 22 | PDB header:hydrolase Chain: A: PDB Molecule:dipeptidyl peptidase iv; PDBTitle: crystal structure of dipeptidyl aminopeptidase iv from2 stenotrophomonas maltophilia | |
| 130 | WP_003471132.1 | c3obkH | 100 | 99 | 52 | PDB header:lyase Chain: H: PDB Molecule:delta-aminolevulinic acid dehydratase; PDBTitle: crystal structure of delta-aminolevulinic acid dehydratase2 (porphobilinogen synthase) from toxoplasma gondii me49 in complex3 with the reaction product porphobilinogen | |
| 131 | WP_003471196.1 | c4hg0A | 100 | 79 | 48 | PDB header:transport protein Chain: A: PDB Molecule:magnesium and cobalt efflux protein corc; PDBTitle: crystal structure of magnesium and cobalt efflux protein corc,2 northeast structural genomics consortium (nesg) target er40 | |
| 132 | WP_003471228.1 | c3breA | 100 | 95 | 29 | PDB header:signaling protein Chain: A: PDB Molecule:probable two-component response regulator; PDBTitle: crystal structure of p.aeruginosa pa3702 | |
| 133 | WP_003471283.1 | c4j2hA | 100 | 94 | 30 | PDB header:oxidoreductase Chain: A: PDB Molecule:short chain alcohol dehydrogenase-related dehydrogenase; PDBTitle: crystal structure of a putative short-chain alcohol dehydrogenase from2 sinorhizobium meliloti 1021 (target nysgrc-011708) | |
| 134 | WP_003471299.1 | c4i3hA | 100 | 97 | 39 | PDB header:isomerase/dna Chain: A: PDB Molecule:topoisomerase iv subunit b, dna topoisomerase 4 subunit a PDBTitle: a three-gate structure of topoisomerase iv from streptococcus2 pneumoniae | |
| 135 | WP_003471310.1 | c2fymA | 100 | 98 | 64 | PDB header:lyase Chain: A: PDB Molecule:enolase; PDBTitle: crystal structure of e. coli enolase complexed with the2 minimal binding segment of rnase e. | |
| 136 | WP_003471314.1 | c2xwlB | 100 | 97 | 32 | PDB header:transferase Chain: B: PDB Molecule:2-c-methyl-d-erythritol 4-phosphate cytidylyltransferase; PDBTitle: crystal structure of ispd from mycobacterium smegmatis in complex2 with ctp and mg | |
| 137 | WP_003471337.1 | c4z8xC | 100 | 71 | 58 | PDB header:hydrolase Chain: C: PDB Molecule:atp-dependent zinc metalloprotease ftsh; PDBTitle: truncated ftsh from a. aeolicus | |
| 138 | WP_003471348.1 | c1t1eA | 100 | 96 | 44 | PDB header:hydrolase Chain: A: PDB Molecule:kumamolisin; PDBTitle: high resolution crystal structure of the intact pro-2 kumamolisin, a sedolisin type proteinase (previously3 called kumamolysin or kscp) | |
| 139 | WP_003471408.1 | c3cgnA | 100 | 98 | 49 | PDB header:isomerase Chain: A: PDB Molecule:peptidyl-prolyl cis-trans isomerase; PDBTitle: crystal structure of thermophilic slyd | |
| 140 | WP_003471593.1 | c3rm5B | 100 | 98 | 30 | PDB header:transferase Chain: B: PDB Molecule:hydroxymethylpyrimidine/phosphomethylpyrimidine kinase PDBTitle: structure of trifunctional thi20 from yeast | |
| 141 | WP_003471664.1 | c2jg6A | 100 | 92 | 30 | PDB header:hydrolase Chain: A: PDB Molecule:dna-3-methyladenine glycosidase; PDBTitle: crystal structure of a 3-methyladenine dna glycosylase i2 from staphylococcus aureus | |
| 142 | WP_003471665.1 | c5fl3A | 100 | 92 | 43 | PDB header:transport protein Chain: A: PDB Molecule:pili retraction protein pilt; PDBTitle: pilt2 from thermus thermophilus | |
| 143 | WP_003471666.1 | c2gszE | 100 | 99 | 49 | PDB header:protein transport Chain: E: PDB Molecule:twitching motility protein pilt; PDBTitle: structure of a. aeolicus pilt with 6 monomers per2 asymmetric unit | |
| 144 | WP_003471682.1 | c3zokB | 100 | 98 | 47 | PDB header:lyase Chain: B: PDB Molecule:3-dehydroquinate synthase; PDBTitle: structure of 3-dehydroquinate synthase from actinidia2 chinensis in complex with nad | |
| 145 | WP_003471820.1 | c2a8cE | 100 | 98 | 48 | PDB header:lyase Chain: E: PDB Molecule:carbonic anhydrase 2; PDBTitle: haemophilus influenzae beta-carbonic anhydrase | |
| 146 | WP_003471844.1 | c2yevA | 100 | 97 | 32 | PDB header:electron transport Chain: A: PDB Molecule:cytochrome c oxidase polypeptide i+iii; PDBTitle: structure of caa3-type cytochrome oxidase | |
| 147 | WP_003471850.1 | c1m56G | 100 | 95 | 54 | PDB header:oxidoreductase Chain: G: PDB Molecule:cytochrome c oxidase; PDBTitle: structure of cytochrome c oxidase from rhodobactor2 sphaeroides (wild type) | |
| 148 | WP_003471878.1 | c3guxA | 100 | 85 | 30 | PDB header:hydrolase Chain: A: PDB Molecule:putative zn-dependent exopeptidase; PDBTitle: crystal structure of a putative zn-dependent exopeptidase (bvu_1317)2 from bacteroides vulgatus atcc 8482 at 1.80 a resolution | |
| 149 | WP_003471965.1 | c2r3aA | 100 | 97 | 30 | PDB header:transferase Chain: A: PDB Molecule:histone-lysine n-methyltransferase suv39h2; PDBTitle: methyltransferase domain of human suppressor of variegation2 3-9 homolog 2 | |
| 150 | WP_003471967.1 | c2vrnA | 100 | 96 | 42 | PDB header:hydrolase Chain: A: PDB Molecule:protease i; PDBTitle: the structure of the stress response protein dr1199 from2 deinococcus radiodurans: a member of the dj-1 superfamily | |
| 151 | WP_003472015.1 | c3i47A | 100 | 98 | 47 | PDB header:lyase Chain: A: PDB Molecule:enoyl coa hydratase/isomerase (crotonase); PDBTitle: crystal structure of putative enoyl coa hydratase/isomerase2 (crotonase) from legionella pneumophila subsp. pneumophila str.3 philadelphia 1 | |
| 152 | WP_003472018.1 | c4o5oA | 100 | 99 | 55 | PDB header:oxidoreductase Chain: A: PDB Molecule:3-hydroxyacyl-coa dehydrogenase; PDBTitle: x-ray crystal structure of a 3-hydroxyacyl-coa dehydrogenase from2 brucella suis | |
| 153 | WP_003472081.1 | c4zrsA | 100 | 83 | 25 | PDB header:hydrolase Chain: A: PDB Molecule:esterase; PDBTitle: crystal structure of a cloned feruloyl esterase from a soil2 metagenomic library | |
| 154 | WP_003472086.1 | c2lssA | 99.9 | 97 | 40 | PDB header:rna binding protein, dna binding protein Chain: A: PDB Molecule:cold shock-like protein; PDBTitle: solution structure of the r. rickettsii cold shock-like protein | |
| 155 | WP_003472098.1 | c4afnB | 100 | 99 | 44 | PDB header:oxidoreductase Chain: B: PDB Molecule:3-oxoacyl-[acyl-carrier-protein] reductase fabg; PDBTitle: crystal structure of 3-ketoacyl-(acyl-carrier-protein) reductase2 (fabg) from pseudomonas aeruginosa at 2.3a resolution | |
| 156 | WP_003472119.1 | c3h4lB | 100 | 52 | 27 | PDB header:dna binding protein, protein binding Chain: B: PDB Molecule:dna mismatch repair protein pms1; PDBTitle: crystal structure of n terminal domain of a dna repair protein | |
| 157 | WP_003472142.1 | c1x55A | 100 | 97 | 39 | PDB header:ligase Chain: A: PDB Molecule:asparaginyl-trna synthetase; PDBTitle: crystal structure of asparaginyl-trna synthetase from pyrococcus2 horikoshii complexed with asparaginyl-adenylate analogue | |
| 158 | WP_003472490.1 | c3dldA | 100 | 98 | 79 | PDB header:hydrolase Chain: A: PDB Molecule:peptide deformylase; PDBTitle: crystal structure of peptide deformylase, xoo1075, from xanthomonas2 oryzae pv. oryzae kacc10331 | |
| 159 | WP_003472589.1 | c1eqrC | 100 | 99 | 55 | PDB header:ligase Chain: C: PDB Molecule:aspartyl-trna synthetase; PDBTitle: crystal structure of free aspartyl-trna synthetase from2 escherichia coli | |
| 160 | WP_003472647.1 | c4h60A | 100 | 98 | 26 | PDB header:signaling protein Chain: A: PDB Molecule:chemotaxis protein chey; PDBTitle: high resolution structure of vibrio cholerae chemotaxis protein chey42 crystallized in low ph (4.0) condition | |
| 161 | WP_003472648.1 | c2ayxA | 100 | 98 | 26 | PDB header:transferase Chain: A: PDB Molecule:sensor kinase protein rcsc; PDBTitle: solution structure of the e.coli rcsc c-terminus (residues2 700-949) containing linker region and phosphoreceiver3 domain | |
| 162 | WP_003472675.1 | c1hyuA | 100 | 97 | 64 | PDB header:oxidoreductase Chain: A: PDB Molecule:alkyl hydroperoxide reductase subunit f; PDBTitle: crystal structure of intact ahpf | |
| 163 | WP_003472685.1 | c5bnzA | 100 | 96 | 60 | PDB header:ligase Chain: A: PDB Molecule:glutamine--trna ligase; PDBTitle: crystal structure of glutamine-trna ligase /glutaminyl-trna synthetase2 (glnrs) from pseudomonas aeruginosa | |
| 164 | WP_003472695.1 | c3ocqA | 100 | 82 | 28 | PDB header:hydrolase Chain: A: PDB Molecule:putative cytosine/adenosine deaminase; PDBTitle: crystal structure of trna-specific adenosine deaminase from salmonella2 enterica | |
| 165 | WP_003472750.1 | c5c7tB | 100 | 94 | 27 | PDB header:hydrolase Chain: B: PDB Molecule:nudf protein; PDBTitle: crystal structure of the bdellovibrio bacteriovorus nucleoside2 diphosphate sugar hydrolase in complex with adp-ribose | |
| 166 | WP_003472753.1 | c3b8bA | 100 | 94 | 41 | PDB header:hydrolase Chain: A: PDB Molecule:cysq, sulfite synthesis pathway protein; PDBTitle: crystal structure of cysq from bacteroides thetaiotaomicron, a2 bacterial member of the inositol monophosphatase family | |
| 167 | WP_003472789.1 | c5mkkA | 100 | 98 | 32 | PDB header:transport protein Chain: A: PDB Molecule:multidrug resistance abc transporter atp-binding and PDBTitle: crystal structure of the heterodimeric abc transporter tmrab, a2 homolog of the antigen translocation complex tap | |
| 168 | WP_003472896.1 | c1q6uA | 100 | 90 | 29 | PDB header:isomerase Chain: A: PDB Molecule:fkbp-type peptidyl-prolyl cis-trans isomerase fkpa; PDBTitle: crystal structure of fkpa from escherichia coli | |
| 169 | WP_003472954.1 | c2cfoA | 100 | 99 | 41 | PDB header:ligase Chain: A: PDB Molecule:glutamyl-trna synthetase; PDBTitle: non-discriminating glutamyl-trna synthetase from2 thermosynechococcus elongatus in complex with glu | |
| 170 | WP_003473108.1 | c3r3rA | 100 | 97 | 47 | PDB header:transferase Chain: A: PDB Molecule:ferripyochelin binding protein; PDBTitle: structure of the yrda ferripyochelin binding protein from salmonella2 enterica | |
| 171 | WP_003473181.1 | c5gpeB | 100 | 95 | 32 | PDB header:transcription Chain: B: PDB Molecule:transcriptional regulator, merr-family; PDBTitle: crystal structure of the transcription regulator pbrr691 from2 ralstonia metallidurans ch34 in complex with lead(ii) | |
| 172 | WP_003473187.1 | c4ka8A | 100 | 99 | 40 | PDB header:hydrolase Chain: A: PDB Molecule:oligopeptidase a; PDBTitle: structure of organellar oligopeptidase | |
| 173 | WP_003473271.1 | c3lutA | 100 | 99 | 40 | PDB header:membrane protein Chain: A: PDB Molecule:voltage-gated potassium channel subunit beta-2; PDBTitle: a structural model for the full-length shaker potassium channel kv1.2 | |
| 174 | WP_003473476.1 | c1yt3A | 100 | 98 | 31 | PDB header:hydrolase,translation Chain: A: PDB Molecule:ribonuclease d; PDBTitle: crystal structure of escherichia coli rnase d, an2 exoribonuclease involved in structured rna processing | |
| 175 | WP_003475546.1 | c3vx3A | 100 | 89 | 32 | PDB header:adp binding protein Chain: A: PDB Molecule:atpase involved in chromosome partitioning, para/mind PDBTitle: crystal structure of [nife] hydrogenase maturation protein hypb from2 thermococcus kodakarensis kod1 | |
| 176 | WP_003476601.1 | c2rccC | 100 | 89 | 39 | PDB header:oxidoreductase Chain: C: PDB Molecule:ribonucleoside-diphosphate reductase subunit beta; PDBTitle: crystal structure of putative class i ribonucleotide reductase2 (np_241368.1) from bacillus halodurans at 1.90 a resolution | |
| 177 | WP_003477416.1 | c2kn9A | 100 | 95 | 54 | PDB header:electron transport Chain: A: PDB Molecule:rubredoxin; PDBTitle: solution structure of zinc-substituted rubredoxin b (rv3250c) from2 mycobacterium tuberculosis. seattle structural genomics center for3 infectious disease target mytud.01635.a | |
| 178 | WP_003477578.1 | c1c0mA | 99.7 | 61 | 23 | PDB header:transferase Chain: A: PDB Molecule:protein (integrase); PDBTitle: crystal structure of rsv two-domain integrase | |
| 179 | WP_003480258.1 | c4b7cB | 100 | 97 | 47 | PDB header:oxidoreductase Chain: B: PDB Molecule:probable oxidoreductase; PDBTitle: crystal structure of hypothetical protein pa1648 from2 pseudomonas aeruginosa. | |
| 180 | WP_003480260.1 | c4xk2A | 100 | 99 | 38 | PDB header:oxidoreductase Chain: A: PDB Molecule:aldo/keto reductase; PDBTitle: crystal structure of aldo-keto reductase from polaromonas sp. js666 | |
| 181 | WP_003481247.1 | c5c7tB | 100 | 97 | 28 | PDB header:hydrolase Chain: B: PDB Molecule:nudf protein; PDBTitle: crystal structure of the bdellovibrio bacteriovorus nucleoside2 diphosphate sugar hydrolase in complex with adp-ribose | |
| 182 | WP_003481607.1 | c4kfcB | 100 | 93 | 37 | PDB header:transcription regulator/dna Chain: B: PDB Molecule:kdp operon transcriptional regulatory protein kdpe; PDBTitle: crystal structure of a hyperactive mutant of response regulator kdpe2 complexed to its promoter dna | |
| 183 | WP_003488188.1 | c3a0jB | 99.9 | 98 | 58 | PDB header:transcription Chain: B: PDB Molecule:cold shock protein; PDBTitle: crystal structure of cold shock protein 1 from thermus2 thermophilus hb8 | |
| 184 | WP_004425452.1 | c3dhwC | 100 | 99 | 49 | PDB header:membrane protein/hydrolase Chain: C: PDB Molecule:methionine import atp-binding protein metn; PDBTitle: crystal structure of methionine importer metni | |
| 185 | WP_004425502.1 | c2ragB | 100 | 91 | 46 | PDB header:hydrolase Chain: B: PDB Molecule:dipeptidase; PDBTitle: crystal structure of aminohydrolase from caulobacter crescentus | |
| 186 | WP_004425521.1 | c4z9yA | 100 | 99 | 63 | PDB header:oxidoreductase Chain: A: PDB Molecule:2-deoxy-d-gluconate 3-dehydrogenase; PDBTitle: crystal structure of 2-keto-3-deoxy-d-gluconate dehydrogenase from2 pectobacterium carotovorum | |
| 187 | WP_004425530.1 | c4fg5B | 100 | 94 | 25 | PDB header:hydrolase Chain: B: PDB Molecule:e3 alpha-esterase-7 caboxylesterase; PDBTitle: crystal structure of the alpha-esterase-7 carboxylesterase, e3, from2 lucilia cuprina | |
| 188 | WP_004425664.1 | c3dkuB | 100 | 93 | 43 | PDB header:hydrolase Chain: B: PDB Molecule:putative phosphohydrolase; PDBTitle: crystal structure of nudix hydrolase orf153, ymfb, from2 escherichia coli k-1 | |
| 189 | WP_004425670.1 | c1r6bX | 100 | 98 | 65 | PDB header:hydrolase Chain: X: PDB Molecule:clpa protein; PDBTitle: high resolution crystal structure of clpa | |
| 190 | WP_004425677.1 | c4ql5A | 100 | 93 | 69 | PDB header:translation Chain: A: PDB Molecule:translation initiation factor if-1; PDBTitle: crystal structure of translation initiation factor if-1 from2 streptococcus pneumoniae tigr4 | |
| 191 | WP_004425727.1 | c4m98A | 100 | 94 | 25 | PDB header:transferase Chain: A: PDB Molecule:pilin glycosylation protein; PDBTitle: acetyltransferase domain of pglb from neisseria gonorrhoeae fa1090 | |
| 192 | WP_004425920.1 | c1y791 | 100 | 94 | 55 | PDB header:hydrolase Chain: 1: PDB Molecule:peptidyl-dipeptidase dcp; PDBTitle: crystal structure of the e.coli dipeptidyl carboxypeptidase2 dcp in complex with a peptidic inhibitor | |
| 193 | WP_004425978.1 | c2et6A | 100 | 88 | 23 | PDB header:oxidoreductase Chain: A: PDB Molecule:(3r)-hydroxyacyl-coa dehydrogenase; PDBTitle: (3r)-hydroxyacyl-coa dehydrogenase domain of candida tropicalis2 peroxisomal multifunctional enzyme type 2 | |
| 194 | WP_004426268.1 | c3g25B | 100 | 99 | 65 | PDB header:transferase Chain: B: PDB Molecule:glycerol kinase; PDBTitle: 1.9 angstrom crystal structure of glycerol kinase (glpk) from2 staphylococcus aureus in complex with glycerol. | |
| 195 | WP_004426362.1 | c1sr9A | 100 | 95 | 26 | PDB header:transferase Chain: A: PDB Molecule:2-isopropylmalate synthase; PDBTitle: crystal structure of leua from mycobacterium tuberculosis | |
| 196 | WP_004426406.1 | c1egaB | 100 | 96 | 48 | PDB header:hydrolase Chain: B: PDB Molecule:protein (gtp-binding protein era); PDBTitle: crystal structure of a widely conserved gtpase era | |
| 197 | WP_004426468.1 | c5e84B | 100 | 99 | 38 | PDB header:chaperone Chain: B: PDB Molecule:78 kda glucose-regulated protein; PDBTitle: atp-bound state of bip | |
| 198 | WP_004426584.1 | c1q6uA | 100 | 62 | 38 | PDB header:isomerase Chain: A: PDB Molecule:fkbp-type peptidyl-prolyl cis-trans isomerase fkpa; PDBTitle: crystal structure of fkpa from escherichia coli | |
| 199 | WP_009592098.1 | c1qvcA | 100 | 81 | 44 | PDB header:dna binding protein Chain: A: PDB Molecule:single stranded dna binding protein monomer; PDBTitle: crystal structure analysis of single stranded dna binding protein2 (ssb) from e.coli | |
| 200 | WP_038236283.1 | c2k8iA | 100 | 45 | 99 | PDB header:isomerase Chain: A: PDB Molecule:peptidyl-prolyl cis-trans isomerase; PDBTitle: solution structure of e.coli slyd | |
| 201 | WP_038236501.1 | c5cioA | 100 | 92 | 17 | PDB header:metal binding protein Chain: A: PDB Molecule:pyrroloquinoline quinone biosynthesis protein pqqf; PDBTitle: crystal structure of pqqf | |
| 202 | WP_038236644.1 | c2ashB | 100 | 96 | 48 | PDB header:transferase Chain: B: PDB Molecule:queuine trna-ribosyltransferase; PDBTitle: crystal structure of queuine trna-ribosyltransferase (ec 2.4.2.29)2 (trna-guanine (tm1561) from thermotoga maritima at 1.90 a resolution | |
| 203 | WP_038236685.1 | c2o7pA | 100 | 95 | 51 | PDB header:hydrolase, oxidoreductase Chain: A: PDB Molecule:riboflavin biosynthesis protein ribd; PDBTitle: the crystal structure of ribd from escherichia coli in complex with2 the oxidised nadp+ cofactor in the active site of the reductase3 domain | |
| 204 | WP_038237017.1 | c3e5kA | 100 | 98 | 37 | PDB header:oxidoreductase Chain: A: PDB Molecule:cytochrome p450 (cytochrome p450 hydroxylase); PDBTitle: crystal structure of cyp105p1 wild-type 4-phenylimidazole complex | |
| 205 | WP_038237239.1 | c1xa0B | 100 | 97 | 45 | PDB header:structural genomics, unknown function Chain: B: PDB Molecule:putative nadph dependent oxidoreductases; PDBTitle: crystal structure of mcsg target apc35536 from bacillus2 stearothermophilus | |
| 206 | WP_038238095.1 | c2j8zA | 100 | 95 | 40 | PDB header:oxidoreductase Chain: A: PDB Molecule:quinone oxidoreductase; PDBTitle: crystal structure of human p53 inducible oxidoreductase (2 tp53i3,pig3) | |
| 207 | WP_038238334.1 | c5x40A | 100 | 88 | 33 | PDB header:transport protein Chain: A: PDB Molecule:cobalt abc transporter atp-binding protein; PDBTitle: structure of a cbio dimer bound with amppcp | |
| 208 | WP_038238529.1 | c4je5C | 100 | 95 | 23 | PDB header:transferase Chain: C: PDB Molecule:aromatic/aminoadipate aminotransferase 1; PDBTitle: crystal structure of the aromatic aminotransferase aro8, a putative2 alpha-aminoadipate aminotransferase in saccharomyces cerevisiae | |
| 209 | WP_038238706.1 | c3l9eC | 100 | 78 | 42 | PDB header:oxidoreductase Chain: C: PDB Molecule:superoxide dismutase [cu-zn]; PDBTitle: crystal structures of holo and cu-deficient cu/znsod from the silkworm2 bombyx mori and the implications in amyotrophic lateral sclerosis | |
| 210 | WP_038238771.1 | c5kiaA | 100 | 98 | 58 | PDB header:oxidoreductase Chain: A: PDB Molecule:l-threonine 3-dehydrogenase; PDBTitle: crystal structure of l-threonine 3-dehydrogenase from burkholderia2 thailandensis | |
| 211 | WP_038238963.1 | c2gb5B | 100 | 87 | 29 | PDB header:hydrolase Chain: B: PDB Molecule:nadh pyrophosphatase; PDBTitle: crystal structure of nadh pyrophosphatase (ec 3.6.1.22) (1790429) from2 escherichia coli k12 at 2.30 a resolution | |
| 212 | WP_038239202.1 | c4g50A | 100 | 87 | 58 | PDB header:isomerase, protein binding Chain: A: PDB Molecule:ubiquitin-like protein smt3, peptidyl-prolyl cis-trans PDBTitle: crystal structure of a smt fusion peptidyl-prolyl cis-trans isomerase2 with surface mutation d44g from burkholderia pseudomallei complexed3 with cj168 | |
| 213 | WP_038239399.1 | c3uorB | 100 | 89 | 76 | PDB header:sugar binding protein Chain: B: PDB Molecule:abc transporter sugar binding protein; PDBTitle: the structure of the sugar-binding protein male from the phytopathogen2 xanthomonas citri | |
| 214 | WP_047324411.1 | c5cioA | 100 | 92 | 16 | PDB header:metal binding protein Chain: A: PDB Molecule:pyrroloquinoline quinone biosynthesis protein pqqf; PDBTitle: crystal structure of pqqf | |
| 215 | WP_047324417.1 | c3ezuA | 100 | 63 | 23 | PDB header:signaling protein Chain: A: PDB Molecule:ggdef domain protein; PDBTitle: crystal structure of multidomain protein of unknown function with2 ggdef-domain (np_951600.1) from geobacter sulfurreducens at 1.95 a3 resolution | |
| 216 | WP_047324421.1 | c3rcmA | 100 | 98 | 55 | PDB header:hydrolase Chain: A: PDB Molecule:tatd family hydrolase; PDBTitle: crystal structure of efi target 500140:tatd family hydrolase from2 pseudomonas putida | |
| 217 | WP_047324427.1 | c2ecfA | 100 | 96 | 77 | PDB header:hydrolase Chain: A: PDB Molecule:dipeptidyl peptidase iv; PDBTitle: crystal structure of dipeptidyl aminopeptidase iv from2 stenotrophomonas maltophilia | |
| 218 | WP_047324433.1 | c3pwzA | 100 | 96 | 44 | PDB header:oxidoreductase Chain: A: PDB Molecule:shikimate dehydrogenase 3; PDBTitle: crystal structure of an ael1 enzyme from pseudomonas putida | |
| 219 | WP_047324452.1 | c5idxB | 100 | 99 | 71 | PDB header:oxidoreductase Chain: B: PDB Molecule:short-chain dehydrogenase/reductase sdr; PDBTitle: crystal structure of an oxidoreductase from burkholderia vietnamiensis | |
| 220 | WP_047324462.1 | c3c04A | 100 | 59 | 58 | PDB header:isomerase Chain: A: PDB Molecule:phosphomannomutase/phosphoglucomutase; PDBTitle: structure of the p368g mutant of pmm/pgm from p. Aeruginosa | |
| 221 | WP_047324470.1 | c1uufA | 100 | 97 | 66 | PDB header:oxidoreductase Chain: A: PDB Molecule:zinc-type alcohol dehydrogenase-like protein PDBTitle: crystal structure of a zinc-type alcohol dehydrogenase-like2 protein yahk | |
| 222 | WP_047324475.1 | c1m57H | 100 | 75 | 33 | PDB header:oxidoreductase Chain: H: PDB Molecule:cytochrome c oxidase; PDBTitle: structure of cytochrome c oxidase from rhodobacter2 sphaeroides (eq(i-286) mutant)) | |
| 223 | WP_047324485.1 | c5of4A | 100 | 93 | 23 | PDB header:transcription Chain: A: PDB Molecule:tfiih basal transcription factor complex helicase xpb PDBTitle: the cryo-em structure of human tfiih | |
| 224 | WP_047324501.1 | c2w3tA | 100 | 98 | 55 | PDB header:hydrolase Chain: A: PDB Molecule:peptide deformylase; PDBTitle: chloro complex of the ni-form of e.coli deformylase | |
| 225 | WP_047324520.1 | c5m7nA | 100 | 99 | 36 | PDB header:signaling protein Chain: A: PDB Molecule:nitrogen assimilation regulatory protein; PDBTitle: crystal structure of ntrx from brucella abortus in complex with atp2 processed with the crystaldirect automated mounting and cryo-cooling3 technology | |
| 226 | WP_047324534.1 | c1qvrB | 100 | 92 | 31 | PDB header:chaperone Chain: B: PDB Molecule:clpb protein; PDBTitle: crystal structure analysis of clpb | |
| 227 | WP_047324537.1 | c3ciaA | 100 | 91 | 49 | PDB header:hydrolase Chain: A: PDB Molecule:cold-active aminopeptidase; PDBTitle: crystal structure of cold-aminopeptidase from colwellia2 psychrerythraea | |
| 228 | WP_047324567.1 | c5ji5A | 100 | 98 | 49 | PDB header:hydrolase Chain: A: PDB Molecule:bupha.10154.a.b1; PDBTitle: crystal structure of a histone deacetylase superfamily protein from2 burkholderia phymatumphymatum | |
| 229 | WP_047324573.1 | c5e2eB | 100 | 87 | 41 | PDB header:hydrolase Chain: B: PDB Molecule:beta-lactamase; PDBTitle: crystal structure of beta-lactamase precursor blaa from yersinia2 enterocolitica | |
| 230 | WP_047324579.1 | c3agqA | 100 | 99 | 79 | PDB header:translation,transferase Chain: A: PDB Molecule:elongation factor ts, elongation factor tu 1, linker, q PDBTitle: structure of viral polymerase form ii | |
| 231 | WP_047324585.1 | c4dvjA | 100 | 99 | 49 | PDB header:oxidoreductase Chain: A: PDB Molecule:putative zinc-dependent alcohol dehydrogenase protein; PDBTitle: crystal structure of a putative zinc-dependent alcohol dehydrogenase2 protein from rhizobium etli cfn 42 | |
| 232 | WP_047324608.1 | c3fwlA | 100 | 72 | 25 | PDB header:transferase, hydrolase Chain: A: PDB Molecule:penicillin-binding protein 1b; PDBTitle: crystal structure of the full-length transglycosylase pbp1b2 from escherichia coli | |
| 233 | WP_047324609.1 | c4kxdA | 100 | 96 | 25 | PDB header:hydrolase Chain: A: PDB Molecule:glutamyl aminopeptidase; PDBTitle: crystal structure of human aminopeptidase a complexed with glutamate2 and calcium | |
| 234 | WP_047324617.1 | c2vf7B | 100 | 99 | 71 | PDB header:dna binding protein Chain: B: PDB Molecule:excinuclease abc, subunit a.; PDBTitle: crystal structure of uvra2 from deinococcus radiodurans | |
| 235 | WP_047324619.1 | c2dphA | 100 | 99 | 36 | PDB header:oxidoreductase Chain: A: PDB Molecule:formaldehyde dismutase; PDBTitle: crystal structure of formaldehyde dismutase | |
| 236 | WP_047324635.1 | C1c0mA | 99.8 | 61 | 23 | PDB header:transferase Chain: A: PDB Molecule:protein (integrase); PDBTitle: crystal structure of rsv two-domain integrase | |
| 237 | WP_047324647.1 | c4cdgA | 100 | 99 | 37 | PDB header:hydrolase Chain: A: PDB Molecule:bloom syndrome protein; PDBTitle: crystal structure of the bloom's syndrome helicase blm in2 complex with nanobody | |
| 238 | WP_047324648.1 | c4cy9A | 100 | 84 | 27 | PDB header:iron-binding protein Chain: A: PDB Molecule:dpsa; PDBTitle: dpsa14 from streptomyces coelicolor | |
| 239 | WP_047324659.1 | c2x0kB | 100 | 91 | 34 | PDB header:transferase Chain: B: PDB Molecule:riboflavin biosynthesis protein ribf; PDBTitle: crystal structure of modular fad synthetase from2 corynebacterium ammoniagenes | |
| 240 | WP_047324660.1 | c2yevA | 100 | 90 | 36 | PDB header:electron transport Chain: A: PDB Molecule:cytochrome c oxidase polypeptide i+iii; PDBTitle: structure of caa3-type cytochrome oxidase | |
| 241 | WP_047324671.1 | c4fc6B | 100 | 98 | 26 | PDB header:oxidoreductase Chain: B: PDB Molecule:peroxisomal 2,4-dienoyl-coa reductase; PDBTitle: studies on dcr shed new light on peroxisomal beta-oxidation: crystal2 structure of the ternary complex of pdcr | |
| 242 | WP_047324673.1 | c2vk2A | 100 | 90 | 54 | PDB header:transport protein Chain: A: PDB Molecule:abc transporter periplasmic-binding protein ytfq; PDBTitle: crystal structure of a galactofuranose binding protein | |
| 243 | WP_047324677.1 | c2j289 | 100 | 93 | 67 | PDB header:ribosome Chain: 9: PDB Molecule:signal recognition particle 54; PDBTitle: model of e. coli srp bound to 70s rncs | |
| 244 | WP_047324684.1 | c2d3tB | 100 | 97 | 32 | PDB header:lyase, oxidoreductase/transferase Chain: B: PDB Molecule:fatty oxidation complex alpha subunit; PDBTitle: fatty acid beta-oxidation multienzyme complex from2 pseudomonas fragi, form v | |
| 245 | WP_047324687.1 | c4qjty | 100 | 99 | 58 | PDB header:ribosome Chain: Y: PDB Molecule: PDBTitle: crystal structure of elongation factor 4 (ef4/lepa) bound to the2 thermus thermophilus 70s ribosome, 30s subunit of the 70s ribosome | |
| 246 | WP_047324691.1 | c5lqwC | 100 | 53 | 22 | PDB header:splicing Chain: C: PDB Molecule:pre-mrna-splicing helicase brr2; PDBTitle: yeast activated spliceosome | |
| 247 | WP_047324692.1 | c1x9nA | 100 | 99 | 23 | PDB header:ligase/dna Chain: A: PDB Molecule:dna ligase i; PDBTitle: crystal structure of human dna ligase i bound to 5'-adenylated, nicked2 dna | |
| 248 | WP_047324698.1 | c1lehB | 100 | 98 | 50 | PDB header:oxidoreductase Chain: B: PDB Molecule:leucine dehydrogenase; PDBTitle: leucine dehydrogenase from bacillus sphaericus | |
| 249 | WP_047324715.1 | c5dmnA | 100 | 95 | 69 | PDB header:transferase Chain: A: PDB Molecule:homocysteine s-methyltransferase; PDBTitle: crystal structure of the homocysteine methyltransferase mmum from2 escherichia coli, apo form | |
| 250 | WP_047324717.1 | c3qdnA | 100 | 99 | 31 | PDB header:oxidoreductase Chain: A: PDB Molecule:putative thioredoxin protein; PDBTitle: putative thioredoxin protein from salmonella typhimurium | |
| 251 | WP_047324718.1 | c4ariA | 100 | 99 | 58 | PDB header:ligase/rna Chain: A: PDB Molecule:leucine--trna ligase; PDBTitle: ternary complex of e. coli leucyl-trna synthetase, trna(leu) and the2 benzoxaborole an2679 in the editing conformation | |
| 252 | WP_047324738.1 | c1iqcB | 100 | 96 | 35 | PDB header:oxidoreductase Chain: B: PDB Molecule:di-heme peroxidase; PDBTitle: crystal structure of di-heme peroxidase from nitrosomonas europaea | |
| 253 | WP_047324740.1 | c1ew2A | 100 | 87 | 45 | PDB header:hydrolase Chain: A: PDB Molecule:phosphatase; PDBTitle: crystal structure of a human phosphatase | |
| 254 | WP_047324744.1 | c3zukB | 100 | 96 | 27 | PDB header:hydrolase/inhibitor Chain: B: PDB Molecule:endopeptidase, peptidase family m13; PDBTitle: crystal structure of mycobacterium tuberculosis zinc metalloprotease2 zmp1 in complex with inhibitor | |
| 255 | WP_047324745.1 | c3zukB | 100 | 92 | 40 | PDB header:hydrolase/inhibitor Chain: B: PDB Molecule:endopeptidase, peptidase family m13; PDBTitle: crystal structure of mycobacterium tuberculosis zinc metalloprotease2 zmp1 in complex with inhibitor | |
| 256 | WP_047324753.1 | c3pdkB | 100 | 98 | 42 | PDB header:isomerase Chain: B: PDB Molecule:phosphoglucosamine mutase; PDBTitle: crystal structure of phosphoglucosamine mutase from b. anthracis | |
| 257 | WP_047324755.1 | c3tjrA | 100 | 97 | 23 | PDB header:oxidoreductase Chain: A: PDB Molecule:short chain dehydrogenase; PDBTitle: crystal structure of a rv0851c ortholog short chain dehydrogenase from2 mycobacterium paratuberculosis | |
| 258 | WP_047324760.1 | c3j4jA | 100 | 63 | 45 | PDB header:translation Chain: A: PDB Molecule:translation initiation factor if-2; PDBTitle: model of full-length t. thermophilus translation initiation factor 22 refined against its cryo-em density from a 30s initiation complex map | |
| 259 | WP_047324767.1 | c4r60A | 100 | 98 | 80 | PDB header:hydrolase Chain: A: PDB Molecule:proline dipeptidase; PDBTitle: crystal structure of xaa-pro dipeptidase from xanthomonas campestris | |
| 260 | WP_047324776.1 | c4dzhA | 100 | 98 | 89 | PDB header:hydrolase Chain: A: PDB Molecule:amidohydrolase; PDBTitle: crystal structure of an adenosine deaminase from xanthomonas2 campestris (target nysgrc-200456) with bound zn | |
| 261 | WP_047324777.1 | c4kdcA | 100 | 90 | 53 | PDB header:transferase Chain: A: PDB Molecule:3-demethylubiquinone-9 3-methyltransferase; PDBTitle: crystal structure of ubig | |
| 262 | WP_047324781.1 | c2gwnA | 100 | 99 | 50 | PDB header:structural genomics, unknown function Chain: A: PDB Molecule:dihydroorotase; PDBTitle: the structure of putative dihydroorotase from porphyromonas2 gingivalis. | |
| 263 | WP_047324797.1 | c3uk2B | 100 | 98 | 49 | PDB header:ligase Chain: B: PDB Molecule:pantothenate synthetase; PDBTitle: the structure of pantothenate synthetase from burkholderia2 thailandensis | |
| 264 | WP_047324805.1 | c2f1dP | 100 | 52 | 52 | PDB header:lyase Chain: P: PDB Molecule:imidazoleglycerol-phosphate dehydratase 1; PDBTitle: x-ray structure of imidazoleglycerol-phosphate dehydratase | |
| 265 | WP_047324810.1 | c1qf6A | 100 | 99 | 61 | PDB header:ligase/rna Chain: A: PDB Molecule:threonyl-trna synthetase; PDBTitle: structure of e. coli threonyl-trna synthetase complexed with its2 cognate trna | |
| 266 | WP_047324812.1 | c4p71B | 100 | 99 | 46 | PDB header:ligase Chain: B: PDB Molecule:phenylalanine--trna ligase beta subunit; PDBTitle: apo phers from p. Aeuriginosa | |
| 267 | WP_047324824.1 | c4ct4B | 100 | 81 | 31 | PDB header:rna binding protein Chain: B: PDB Molecule:probable atp-dependent rna helicase ddx6; PDBTitle: cnot1 mif4g domain - ddx6 complex | |
| 268 | WP_047324842.1 | c4j80B | 100 | 92 | 42 | PDB header:chaperone Chain: B: PDB Molecule:chaperone protein dnaj 2; PDBTitle: thermus thermophilus dnaj | |
| 269 | WP_047324869.1 | c2o0rA | 100 | 98 | 43 | PDB header:transferase Chain: A: PDB Molecule:rv0858c (n-succinyldiaminopimelate aminotransferase); PDBTitle: the three-dimensional structure of n-succinyldiaminopimelate2 aminotransferase from mycobacterium tuberculosis | |
| 270 | WP_047324914.1 | c2pg8C | 100 | 95 | 52 | PDB header:ligand binding protein Chain: C: PDB Molecule:dpgc; PDBTitle: crystal structure of r254k mutanat of dpgc with bound substrate analog | |
| 271 | WP_047324929.1 | c3cbfA | 100 | 96 | 48 | PDB header:transferase Chain: A: PDB Molecule:alpha-aminodipate aminotransferase; PDBTitle: crystal structure of lysn, alpha-aminoadipate2 aminotransferase, from thermus thermophilus hb27 | |
| 272 | WP_047324934.1 | c3m97X | 99.9 | 83 |  | PDB header:electron transport Chain: X: PDB Molecule:cytochrome c-552; PDBTitle: structure of the soluble domain of cytochrome c552 with its flexible2 linker segment from paracoccus denitrificans | |
| 273 | WP_047324950.1 | c3pk0B | 100 | 96 | 31 | PDB header:oxidoreductase Chain: B: PDB Molecule:short-chain dehydrogenase/reductase sdr; PDBTitle: crystal structure of short-chain dehydrogenase/reductase sdr from2 mycobacterium smegmatis | |
| 274 | WP_047324951.1 | c4fc6B | 100 | 99 | 25 | PDB header:oxidoreductase Chain: B: PDB Molecule:peroxisomal 2,4-dienoyl-coa reductase; PDBTitle: studies on dcr shed new light on peroxisomal beta-oxidation: crystal2 structure of the ternary complex of pdcr | |
| 275 | WP_047324952.1 | c4m9cC | 100 | 98 | 26 | PDB header:transferase Chain: C: PDB Molecule:bacterial transferase hexapeptide (three repeats) family PDBTitle: weei from acinetobacter baumannii aye | |
| 276 | WP_047324961.1 | c3to5A | 100 | 94 | 80 | PDB header:signaling protein Chain: A: PDB Molecule:chey homolog; PDBTitle: high resolution structure of chey3 from vibrio cholerae | |
| 277 | WP_047324979.1 | c4ex5A | 100 | 97 | 61 | PDB header:transferase Chain: A: PDB Molecule:lysine--trna ligase; PDBTitle: crystal structure of lysyl-trna synthetase lysrs from burkholderia2 thailandensis bound to lysine | |
| 278 | WP_047324986.1 | c3wqyB | 100 | 99 | 32 | PDB header:ligase/rna Chain: B: PDB Molecule:alanine--trna ligase; PDBTitle: crystal structure of archaeoglobus fulgidus alanyl-trna synthetase in2 complex with wild-type trna(ala) having g3.u70 | |
| 279 | WP_047325000.1 | c3fvqB | 100 | 94 | 36 | PDB header:hydrolase Chain: B: PDB Molecule:fe(3+) ions import atp-binding protein fbpc; PDBTitle: crystal structure of the nucleotide binding domain fbpc complexed with2 atp | |
| 280 | WP_047325029.1 | c3cwbQ | 100 | 90 | 28 | PDB header:oxidoreductase Chain: Q: PDB Molecule:mitochondrial cytochrome c1, heme protein; PDBTitle: chicken cytochrome bc1 complex inhibited by an iodinated analogue of2 the polyketide crocacin-d | |
| 281 | WP_047325048.1 | c4mcwA | 100 | 57 | 42 | PDB header:hydrolase Chain: A: PDB Molecule:metal dependent phosphohydrolase; PDBTitle: metallo-enzyme from p. marina | |
| 282 | WP_047325053.1 | c4unfA | 100 | 96 | 29 | PDB header:lyase Chain: A: PDB Molecule:endonuclease iii-1; PDBTitle: crystal structure of deinococcus radiodurans endonuclease iii-1 | |
| 283 | WP_047325063.1 | c3r2rA | 100 | 95 | 70 | PDB header:metal binding protein Chain: A: PDB Molecule:bacterioferritin; PDBTitle: 1.65a resolution structure of iron soaked ftna from pseudomonas2 aeruginosa (ph 6.0) | |
| 284 | WP_047325067.1 | c1vgyB | 100 | 99 | 54 | PDB header:structural genomics, unknown function Chain: B: PDB Molecule:succinyl-diaminopimelate desuccinylase; PDBTitle: crystal structure of succinyl diaminopimelate desuccinylase | |
| 285 | WP_047325082.1 | c2qmiH | 100 | 92 | 26 | PDB header:hydrolase Chain: H: PDB Molecule:pbp related beta-lactamase; PDBTitle: structure of the octameric penicillin-binding protein2 homologue from pyrococcus abyssi | |
| 286 | WP_047325085.1 | c4ljyA | 100 | 58 | 31 | PDB header:hydrolase Chain: A: PDB Molecule:pre-mrna-processing atp-dependent rna helicase prp5; PDBTitle: crystal structure of rna splicing effector prp5 in complex with adp | |
| 287 | WP_047325089.1 | c2hcyD | 100 | 99 | 28 | PDB header:oxidoreductase Chain: D: PDB Molecule:alcohol dehydrogenase 1; PDBTitle: yeast alcohol dehydrogenase i, saccharomyces cerevisiae fermentative2 enzyme | |
| 288 | WP_047325093.1 | c2eyqA | 100 | 99 | 52 | PDB header:hydrolase Chain: A: PDB Molecule:transcription-repair coupling factor; PDBTitle: crystal structure of escherichia coli transcription-repair2 coupling factor | |
| 289 | WP_047325103.1 | c5l8sD | 100 | 96 | 22 | PDB header:hydrolase Chain: D: PDB Molecule:amino acyl peptidase; PDBTitle: the crystal structure of a cold-adapted acylaminoacyl peptidase2 reveals a novel quaternary architecture based on the arm-exchange3 mechanism | |
| 290 | WP_047325123.1 | c4exqA | 100 | 99 | 63 | PDB header:biosynthetic protein Chain: A: PDB Molecule:uroporphyrinogen decarboxylase; PDBTitle: crystal structure of uroporphyrinogen decarboxylase (upd) from2 burkholderia thailandensis e264 | |
| 291 | WP_047325129.1 | c3r9cA | 100 | 95 | 31 | PDB header:oxidoreductase Chain: A: PDB Molecule:cytochrome p450 164a2; PDBTitle: crystal structure of mycobacterium smegmatis cyp164a2 with econazole2 bound | |
| 292 | WP_047325137.1 | c3rcnA | 100 | 61 | 45 | PDB header:hydrolase Chain: A: PDB Molecule:beta-n-acetylhexosaminidase; PDBTitle: crystal structure of beta-n-acetylhexosaminidase from arthrobacter2 aurescens | |
| 293 | WP_047325171.1 | c1cmwA | 100 | 94 | 35 | PDB header:transferase Chain: A: PDB Molecule:protein (dna polymerase i); PDBTitle: crystal structure of taq dna-polymerase shows a new orientation for2 the structure-specific nuclease domain | |
| 294 | WP_047325188.1 | c4f4hA | 100 | 99 | 47 | PDB header:ligase Chain: A: PDB Molecule:glutamine dependent nad+ synthetase; PDBTitle: crystal structure of a glutamine dependent nad+ synthetase from2 burkholderia thailandensis | |
| 295 | WP_047325199.1 | c3iibA | 100 | 98 | 51 | PDB header:hydrolase Chain: A: PDB Molecule:peptidase m28; PDBTitle: crystal structure of peptidase m28 precursor (yp_926796.1) from2 shewanella amazonensis sb2b at 1.70 a resolution | |
| 296 | WP_047325217.1 | c3w52A | 100 | 90 | 32 | PDB header:hydrolase Chain: A: PDB Molecule:endonuclease 2; PDBTitle: zinc-dependent bifunctional nuclease | |
| 297 | WP_047325218.1 | c4efzB | 100 | 98 | 42 | PDB header:hydrolase Chain: B: PDB Molecule:metallo-beta-lactamase family protein; PDBTitle: crystal structure of a hypothetical metallo-beta-lactamase from2 burkholderia pseudomallei | |
| 298 | WP_047325232.1 | c1w7vD | 100 | 97 | 44 | PDB header:hydrolase Chain: D: PDB Molecule:xaa-pro aminopeptidase; PDBTitle: znmg substituted aminopeptidase p from e. coli | |
| 299 | WP_047325244.1 | c3u1hA | 100 | 97 | 59 | PDB header:oxidoreductase Chain: A: PDB Molecule:3-isopropylmalate dehydrogenase; PDBTitle: crystal structure of ipmdh from the last common ancestor of bacillus | |
| 300 | WP_047325261.1 | c5jp6A | 100 | 72 | 28 | PDB header:hydrolase Chain: A: PDB Molecule:putative polysaccharide deacetylase; PDBTitle: bdellovibrio bacteriovorus peptidoglycan deacetylase bd3279 | |
| 301 | WP_047325264.1 | c4b6gA | 100 | 99 | 61 | PDB header:hydrolase Chain: A: PDB Molecule:putative esterase; PDBTitle: the crystal structure of the neisserial esterase d. | |
| 302 | WP_047325265.1 | c1ma0B | 100 | 99 | 64 | PDB header:oxidoreductase Chain: B: PDB Molecule:glutathione-dependent formaldehyde dehydrogenase; PDBTitle: ternary complex of human glutathione-dependent formaldehyde2 dehydrogenase with nad+ and dodecanoic acid | |
| 303 | WP_047325269.1 | c5lo9B | 100 | 80 | 30 | PDB header:oxidoreductase Chain: B: PDB Molecule:cytochrome c; PDBTitle: thiosulfate dehydrogenase (tsdba) from marichromatium purpuratum - "as2 isolated" form | |
| 304 | WP_047325270.1 | c2remB | 100 | 86 | 61 | PDB header:oxidoreductase Chain: B: PDB Molecule:disulfide oxidoreductase; PDBTitle: crystal structure of oxidoreductase dsba from xylella2 fastidiosa | |
| 305 | WP_047325273.1 | c2p50C | 100 | 98 | 41 | PDB header:hydrolase Chain: C: PDB Molecule:n-acetylglucosamine-6-phosphate deacetylase; PDBTitle: crystal structure of n-acetyl-d-glucosamine-6-phosphate deacetylase2 liganded with zn | |
| 306 | WP_047325314.1 | c1gaxB | 100 | 99 | 44 | PDB header:ligase/rna Chain: B: PDB Molecule:valyl-trna synthetase; PDBTitle: crystal structure of thermus thermophilus valyl-trna2 synthetase complexed with trna(val) and valyl-adenylate3 analogue | |
| 307 | WP_047325324.1 | c4pxaA | 100 | 82 | 42 | PDB header:translation, rna binding protein Chain: A: PDB Molecule:atp-dependent rna helicase ddx3x; PDBTitle: dead-box rna helicase ddx3x cancer-associated mutant d354v | |
| 308 | WP_047325406.1 | c4ljyA | 100 | 99 | 30 | PDB header:hydrolase Chain: A: PDB Molecule:pre-mrna-processing atp-dependent rna helicase prp5; PDBTitle: crystal structure of rna splicing effector prp5 in complex with adp | |
| 309 | WP_047325420.1 | c4wecA | 100 | 99 | 31 | PDB header:oxidoreductase Chain: A: PDB Molecule:short chain dehydrogenase; PDBTitle: crystal structure of a short chain dehydrogenase from mycobacterium2 smegmatis | |
| 310 | WP_047325424.1 | c4q1vA | 100 | 92 | 23 | PDB header:hydrolase Chain: A: PDB Molecule:putative dipeptidyl aminopeptidase iv; PDBTitle: crystal structure of a putative dipeptidyl aminopeptidase iv2 (bacova_01349) from bacteroides ovatus atcc 8483 at 2.48 a resolution | |
| 311 | WP_047325433.1 | c5nikK | 100 | 99 | 41 | PDB header:transport protein Chain: K: PDB Molecule:macrolide export atp-binding/permease protein macb; PDBTitle: structure of the macab-tolc abc-type tripartite multidrug efflux pump | |
| 312 | WP_047325436.1 | c2ek8A | 100 | 91 | 27 | PDB header:hydrolase Chain: A: PDB Molecule:aminopeptidase; PDBTitle: aminopeptidase from aneurinibacillus sp. strain am-1 | |
| 313 | WP_047325451.1 | c1nijA | 100 | 92 | 29 | PDB header:structural genomics, unknown function Chain: A: PDB Molecule:hypothetical protein yjia; PDBTitle: yjia protein | |
| 314 | WP_047325465.1 | c2ecfA | 100 | 96 | 24 | PDB header:hydrolase Chain: A: PDB Molecule:dipeptidyl peptidase iv; PDBTitle: crystal structure of dipeptidyl aminopeptidase iv from2 stenotrophomonas maltophilia | |
| 315 | WP_047325488.1 | c4d0jD | 100 | 95 | 34 | PDB header:hydrolase Chain: D: PDB Molecule:beta-glucosidase; PDBTitle: crystal structure of glycoside hydrolase family 3 beta-2 glucosidase cel3a from the moderately thermophilic fungus3 rasamsonia emersonii | |
| 316 | WP_047325492.1 | c4xkmB | 100 | 97 | 62 | PDB header:isomerase Chain: B: PDB Molecule:xylose isomerase; PDBTitle: crystal structure of xylose isomerase from an human intestinal tract2 microbe bacteroides thetaiotaomicron | |
| 317 | WP_047325525.1 | c2bh7A | 100 | 85 | 39 | PDB header:hydrolase Chain: A: PDB Molecule:n-acetylmuramoyl-l-alanine amidase; PDBTitle: crystal structure of a semet derivative of amid at 2.22 angstroms | |
| 318 | WP_047325527.1 | c4nhoA | 100 | 66 | 36 | PDB header:hydrolase Chain: A: PDB Molecule:probable atp-dependent rna helicase ddx23; PDBTitle: structure of the spliceosomal dead-box protein prp28 | |
| 319 | WP_047325529.1 | c3lf2B | 100 | 97 | 30 | PDB header:oxidoreductase Chain: B: PDB Molecule:short chain oxidoreductase q9hya2; PDBTitle: nadph bound structure of the short chain oxidoreductase q9hya2 from2 pseudomonas aeruginosa pao1 containing an atypical catalytic center | |
| 320 | WP_047325583.1 | c5ldgA | 100 | 97 | 38 | PDB header:oxidoreductase Chain: A: PDB Molecule:(-)-isopiperitenone reductase; PDBTitle: isopiperitenone reductase from mentha piperita in complex with2 isopiperitenone and nadp | |
| 321 | WP_047325628.1 | c3moyA | 100 | 99 | 44 | PDB header:lyase Chain: A: PDB Molecule:probable enoyl-coa hydratase; PDBTitle: crystal structure of probable enoyl-coa hydratase from mycobacterium2 smegmatis | |
| 322 | WP_047325669.1 | d1x6ma | 100 | 98 | 66 | Fold:Mss4-like Superfamily:Mss4-like Family:Glutathione-dependent formaldehyde-activating enzyme, Gfa | |
| 323 | WP_047325670.1 | c2remB | 100 | 67 | 45 | PDB header:oxidoreductase Chain: B: PDB Molecule:disulfide oxidoreductase; PDBTitle: crystal structure of oxidoreductase dsba from xylella2 fastidiosa | |
| 324 | WP_047325674.1 | c4i14B | 100 | 94 | 45 | PDB header:hydrolase, lyase Chain: B: PDB Molecule:riboflavin biosynthesis protein ribba; PDBTitle: crystal structure of mtb-riba2 (rv1415) | |
| 325 | WP_047325700.1 | c4xk2A | 100 | 96 | 26 | PDB header:oxidoreductase Chain: A: PDB Molecule:aldo/keto reductase; PDBTitle: crystal structure of aldo-keto reductase from polaromonas sp. js666 | |
| 326 | WP_047325709.1 | c2ic7A | 100 | 97 | 51 | PDB header:transferase Chain: A: PDB Molecule:maltose transacetylase; PDBTitle: crystal structure of maltose transacetylase from2 geobacillus kaustophilus | |
| 327 | WP_053008585.1 | c4je5C | 100 | 88 | 23 | PDB header:transferase Chain: C: PDB Molecule:aromatic/aminoadipate aminotransferase 1; PDBTitle: crystal structure of the aromatic aminotransferase aro8, a putative2 alpha-aminoadipate aminotransferase in saccharomyces cerevisiae | |
| 328 | WP_053008603.1 | c2h47C | 100 | 97 | 46 | PDB header:oxidoreductase/electron transport Chain: C: PDB Molecule:azurin; PDBTitle: crystal structure of an electron transfer complex between2 aromatic amine dephydrogenase and azurin from alcaligenes3 faecalis (form 1) | |
| 329 | WP_080627636.1 | c4fo7B | 100 | 98 | 67 | PDB header:hydrolase Chain: B: PDB Molecule:methionine aminopeptidase; PDBTitle: pseudomonas aeruginosa metap, in mn form | |
| 330 | WP_080964761.1 | c4i3gB | 100 | 69 | 38 | PDB header:hydrolase Chain: B: PDB Molecule:beta-glucosidase; PDBTitle: crystal structure of desr, a beta-glucosidase from streptomyces2 venezuelae in complex with d-glucose. | |
| 331 | WP_080964784.1 | c2ix1A | 100 | 79 | 29 | PDB header:hydrolase Chain: A: PDB Molecule:exoribonuclease 2; PDBTitle: rnase ii d209n mutant | |
| 332 | WP_080964790.1 | c2gu1A | 100 | 69 | 25 | PDB header:hydrolase Chain: A: PDB Molecule:zinc peptidase; PDBTitle: crystal structure of a zinc containing peptidase from2 vibrio cholerae | |
| 333 | WP_080964812.1 | c3j09A | 100 | 90 | 43 | PDB header:hydrolase, metal transport Chain: A: PDB Molecule:copper-exporting p-type atpase a; PDBTitle: high resolution helical reconstruction of the bacterial p-type atpase2 copper transporter copa | |
| 334 | WP_080964844.1 | c3x17B | 100 | 91 | 40 | PDB header:hydrolase Chain: B: PDB Molecule:endoglucanase; PDBTitle: crystal structure of metagenome-derived glycoside hydrolase family 92 endoglucanase | |
| 335 | WP_080964854.1 | c5tf0B | 100 | 95 | 37 | PDB header:hydrolase Chain: B: PDB Molecule:glycosyl hydrolase family 3 n-terminal domain protein; PDBTitle: crystal structure of glycosil hydrolase family 3 n-terminal domain2 protein from bacteroides intestinalis | |

**Supplementary material table S2: Binding residues for Zn-binding proteins of *Xtu***

| **S. No.** | **Sequence Id of putative Zn-binding protein modeled by Phyre2** | **Binding sites in putative Zn-binding protein predicted by MIB server** | **Template on the basis of which MIB predicted the Zn-binding sites in the putatively modeled Zn-binding proteins** | **Binding Score of the prediction/docking provided by MIB server** | **Interaction analysis in the binding sites of putative Zn-binding protein predicted by MIB server using Ligplot^+^ visualizer tool** |
| --- | --- | --- | --- | --- | --- |
| 1 | WP_003465234.1 | 117R ,  120R | 1l9hA1 | 1.188 | 120R NH2 1.90, NE 2.95 |
| 2 | WP_003465242.1 | 47E ,  50E | 3caoA3 | 1.4 | 46R NH1 4.72, NH2 4.26, 50E OE1 2.60 |
| 3 | WP_003465267.1 | 359E ,  363D | 1a7w_0 | 1.688 | 359E O 4.26, 363D OD2, 407R NH1 3.36 |
| 4 | WP_003465369.1 | 180H ,  181D | 1udtA0 | 1.495 | 180H NE2 3.59, 181D OD2 2.14, 354Q NE2 4.85 |
| 5 | WP_003465432.1 | 45D ,  49T | 1pv9A1 | 1.049 | 45D O 4.20, 49T OG1 2.52, 97K NZ 3.99 |
| 6 | WP_003465445.1 | 215E ,  218E | 3caoA3 | 1.68 | 215E OE2 4.70, 218E OE1 2.46 |
| 7 | WP_003465502.1 | 275E ,  278E | 3caoA3 | 1.864 | 274M O 4.43, 278E OE1 3.75 |
| 8 | WP_003465522.1 | 115H ,  118H | 1fioA1 | 1.788 | 115H ND1 2.34, 246V O 3.63 |
| 9 | WP_003465615.1 | 196T ,  200E | 7mdhB4 | 1.4 | 196T OG1 4.96, 200E OE1 3.97 |
| 10 | WP_003465620.1 | 86D ,  87D | 1bawA0 | 1.964 | 87D N 4.15, OD2 4.40, 99Q N 4.68 |
| 11 | WP_003465625.1 | 447D ,  449H ,  684D | 1c3rA0 | 1.805 | 447D OD1 2.04, 448T OG1 3.95, 449H N 3.62, 450N N 4.04, 684D O 4.49 |
| 12 | WP_003465697.1 | 170H ,  171D | 258lA0 | 1.481 | 168N ND2 2.81, 171D OD2 2.16, 215R O 4.20 |
| 13 | WP_003465707.1 | 118D ,  119H | 1ei6A0 | 1.705 | 118D OD2 4.13, 119H ND1 3.32 |
| 14 | WP_003465766.1 | 353D ,  354D | 1bawA0 | 1.467 | 352N ND2 3.39, N 3.70, 354D N 4.15, 357S OG 3.63, O 3.20, N 3.85 |
| 15 | WP_003465837.1 | 262H ,  263E | 1k9zA6 | 1.849 | 205R NH1 4.65, NH2 3.76, 259E O 4.51, 260A O 4.66, 263E N 4.94 |
| 16 | WP_003465861.1 | 57H ,  84C ,  87C | 1rb7A0 | 2.41 | 57H ND1 2.16, 59E OE1 4.06, 84C SG, N 3.85, 87C SG 2.44, N 3.83 |
| 17 | WP_003465899.1 | 36E ,  39H | 1b71A0 | 1.531 | 35N O 4.60, 39H ND1 2.56 |
| 18 | WP_003465966.1 | 14A ,  17D ,  18H | 1ei6A0 | 1.5 | 14A O 3.83, 17D OD2 4.52, 18H NE2 2.40 |
| 19 | WP_003465967.1 | 340K ,  343R | 1l9hA1 | 1.653 | 340K NZ 4.24, 343R NH2 4.02, NE 4.53 |
| 20 | WP_003465985.1 | 447H ,  448D | 1f0jA0 | 1.615 | 446D OD1 3.93, OD2 3.78, 448D OD2 2.29 |
| 21 | WP_003466074.1 | 68E ,  83D ,  85L | 1k9zA1 | 1.849 | 45D OD2 4.07, 68E OE1 4.28, 83D OD1, 85L O 2.11, N 3.57, 88T OG1 3.99, 211D OD1 4.84 |
| 22 | WP_003466157.1 | 144H ,  146H ,  157D | 1k1dA1 | 1.503 | 146H NE2 2.35, 158G N 4.62 |
| 23 | WP_003466231.1 | 552Q ,  553H ,  602C ,  607H ,  612M | 1e67A0 | 1.572 | 508E OE2 3.81, 510H NE2 4.97, 552Q O 4.52, 553H ND1, 602C SG 2.52, 607H ND1 2.33 |
| 24 | WP_003466285.1 | 203H ,  206E | 1uwyA0 | 1.482 | 169E OE1 4.65, 171L N 4.45, 206E OE2 2.31 |
| 25 | WP_003466345.1 | 171H ,  172E | 258lA0 | 1.668 | 168I O 3.77, 172E OE2 2.96 |
| 26 | WP_003466372.1 | 217E ,  221E | 2a0b_0 | 1.301 | 217E O 4.62, 221E OE2 2.64 |
| 27 | WP_003466374.1 | 30E ,  33E | 3caoA3 | 1.886 | 33E OE1 2.91 |
| 28 | WP_003466549.1 | 168H ,  172D | 1ak0_1 | 1.518 | 168H NE2 3.22, 172D OD1 1.91, 175R NH2 4.97 |
| 29 | WP_003466552.1 | 62H ,  92C ,  95C | 1rb7A0 | 2.414 | 62H ND1 2.16, 64E OE1 4.06, 92C SG, N 3.85, 95C SG 2.44, N 3.83 |
| 30 | WP_003466555.1 | 34E ,  37E | 3caoA3 | 1.927 | 37E OE1 2.39, 106R NH1 4.41, 107H NE2 4.60, 132E OE2 2.41 |
| 31 | WP_003466655.1 | 110T ,  114E | 7mdhB4 | 1.513 | 110T 4.93, 114E OE1 4.91 |
| 32 | WP_003466735.1 | 299H ,  300E | 258lA0 | 1.945 | 299H NE2 2 |
| 33 | WP_003466789.1 | 223D ,  225L ,  254E | 1cnqA0 | 1.496 | 186S OG 4.75, 223D OD1 2.33, 224W N 4.45, O 3.95, 253S OG 3.24, N 2.66, O 3.58 |
| 34 | WP_003466792.1 | 228H ,  232E | 1i1iP1 | 1.455 | 228H NE2 2.50, 232E OE2 2.14 |
| 35 | WP_003466794.1 | 237D ,  239D ,  241D | 1kfiA0 | 2.179 | 107K NZ 3.97, 237D OD2 2.10, 239D OD1 2.07, N 4.58, 241D OD1 1.96, N 4.10, 242R N 4.49, NE 4.27 |
| 36 | WP_003466920.1 | 270H ,  274D | 1ibqA2 | 1.394 | 268G O 4.80, 270H N 3.71, 274D OD1 2.81, OD2 2.90 |
| 37 | WP_003466936.1 | 246E ,  250E | 1no5B2 | 1.464 | 250E OE2 2.99, 264M N2.22, 265E N 3.26 |
| 38 | WP_003466959.1 | 208H ,  212H | 1slm_0 | 1.695 | 208H NE2 2.08 |
| 39 | WP_003466981.1 | 481E ,  485E | 2a0b_0 | 1.722 | 481E OE1 4.79, O 4.71, 485E OE2 |
| 40 | WP_003467030.1 | 8D ,  109D ,  112I ,  132D | 1taq_0 | 2.291 | 8D OD2 3.04, 11S OG 4.23, 109D 3.07, 130T OG1 2.77, 132D OD1 2.31 |
| 41 | WP_003467261.1 | 39C ,  98H ,  101C | 1g5cA0 | 2.453 | 39C SG, 41D OD2 3.88, N 4.68, 64G N 4.42, 65N OD1 4.96, 98H NE2 2.08, 101C SG 2.45, 102G 4.02, 103A N 4.79 |
| 42 | WP_003467331.1 | 118E ,  122E | 2a0b_0 | 1.46 | 118E O 4.65, 122E OE1 3.82 |
| 43 | WP_003467341.1 | 241D ,  242D ,  243E | 1mxdA3 | 1.238 | 242D OD1 2.80, N 4.70, 243E N 4.28 |
| 44 | WP_003467345.1 | 11H ,  65D | 1dvfD0 | 1.368 | 64E O 4.40, 65D OD2 2.17 |
| 45 | WP_003467386.1 | 220E ,  224E | 1no5B2 | 1.343 | 224E OE2 4.89 |
| 46 | WP_003467454.1 | 32C ,  35C ,  51C ,  54C | 1qf8A0 | 1.74 | 32C SG 2.80, 35C SG 4.10, 37A N 4.90, 51C SG, 53K N 4.63, 54C SG 2.53, N 4.13 |
| 47 | WP_003467543.1 | 110E ,  113E | 3caoA3 | 1.698 | 113E OE1 2.81 |
| 48 | WP_003467872.1 | 111E ,  115E | 2a0b_0 | 1.425 | 111E OE1 3.68, O 4.71, 115E OE2 |
| 49 | WP_003467936.1 | 34H ,  64Q | 1hp7A0 | 0.932 | 61K NZ 3.64, 62G O 4.94 |
| 50 | WP_003467941.1 | 179K ,  182R | 1l9hA1 | 1.475 | 182R NH2, NE 3.64 |
| 51 | WP_003467943.1 | 7H ,  9H ,  202D | 1hzyA0 | 1.746 | 7H NE2 3, 9H NE2 2.60, 93E OE1 3.72, OE2 3.84, 202D OD2 2, O 4.51, |
| 52 | WP_003467994.1 | 86H ,  88D ,  107E ,  124H | 1mzbA0 | 2.5 | 86H NE2 2.49, 88D OD1 1.99, 107E OE1 2.38, 110Q OE1 4.27, NE2 4.24, 124H NE2 1.93 |
| 53 | WP_003468012.1 | 353Q ,  357E | 1jk0A0 | 1.917 | 353Q NE2 3.45, 357E 2.40 |
| 54 | WP_003468017.1 | 266H ,  270E | 1bi0_0 | 1.53 | 266H NE2 2.88, 270E OE1 3.67 |
| 55 | WP_003468030.1 | 196S ,  197R | 1hbmD0 | 1.87 | 196S O 3.41, 201D OD1 3.34, 197R O 2.40 |
| 56 | WP_003468120.1 | 200D ,  298H | 1cy5A3 | 1.441 | 200D OD2 3.42, 298H O 4.48, 300Y N 2.04, O 2.53 |
| 57 | WP_003468235.1 | 14C ,  94C | 1mwzA0 | 1.373 | 12L O 4.52, 14C SG 3.23, N 3.89, 94C 3.92 |
| 58 | WP_003468255.1 | 117E ,  120E | 3caoA3 | 1.8 | 120E OE1 2.81, 190R NH2 3.63 |
| 59 | WP_003468393.1 | 304Q ,  308E | 1jk0A0 | 1.359 | 308E OE2 1.96 |
| 60 | WP_003468480.1 | 94E ,  97E | 1r4vA0 | 1.686 | 94E 2.97, 97E OE1 2.09 |
| 61 | WP_003468516.1 | 181D ,  182H | 1qh3A1 | 1.449 | 178Q NE2 3.43, O 4.06, 181D OD2 4.43, 182H NE2 2.81 |
| 62 | WP_003468529.1 | 286Q ,  289D ,  290H | 1ei6A0 | 1.482 | 286Q O 3.95, 289D OD2 3.77 |
| 63 | WP_003468538.1 | 71E ,  74E | 3caoA3 | 1.711 | 74EOE1 2.33 |
| 64 | WP_003468546.1 | 52H ,  54H ,  110H | 1smlA1 | 1.549 | 52H NE2 2.26, 53H O 4.56, 55D OD1 4.03, N 4.83, 56D OD1 4.67, 110H NE2, 127D OD1 2.68 |
| 65 | WP_003468608.1 | 51D ,  52D | 1bawA0 | 1.36 | 49T OG1 3.73, C, CA, O, N, 51D N 4.15, 52D N 4.15, 99L N 4.66 |
| 66 | WP_003468623.1 | 282C ,  284R ,  285C ,  356C ,  359C | 2gatA0 | 2.31 | 282C SG, N 3.71, 284R N 4.64, 285C SG 3.47, N 3.74, 356C SG, 359C SG 3.26, N 3.06, 358V N 4.39 |
| 67 | WP_003468634.1 | 154D ,  158E | 1f30A0 | 1.712 | 158E OE2 2.16 |
| 68 | WP_003468654.1 | 372D ,  375L | 1taq_0 | 1.662 | 175E OE2 4.59, 186V O 3.55, 372D OD1 2.22 |
| 69 | WP_003468804.1 | 70E ,  74E | 2a0b_0 | 1.46 | 70E O 4.81, 74E OE2 |
| 70 | WP_003468806.1 | 58E ,  61E | 1r4vA0 | 1.506 | 61E OE2 4.88 |
| 71 | WP_003468858.1 | 59E ,  92E ,  95H | 1mftA0 | 1.967 | 59E OE2 3.62, 142E OE2 4.92, 149Y OH 4.84, 170I O 4.93, 174E OE2 2.89 |
| 72 | WP_003468914.1 | 741C ,  744C ,  748G ,  752V ,  766V ,  767C | 1exkA1 | 2.354 | 741C SG 2.31, 744C SG N 3.26, 743A N 4.47, 748G N 4.82, 767C SG 2.86, N 4.12 |
| 73 | WP_003469000.1 | 148H ,  149E | 258lA0 | 1.583 | 143R NH1 3.13, 149E OE2 2.24 |
| 74 | WP_003469157.1 | 76E ,  77H | 1smlA0 | 1.074 | 76E OE2 3.14 |
| 75 | WP_003469161.1 | 514H ,  578H | 1oekA0 | 1.484 | 512Y OH 2.74, CE1, 576S OG 4.70 |
| 76 | WP_003469170.1 | 109H ,  113D | 1cy5A3 | 1.448 | 113D OD1 4.21, 109H O 4.81 |
| 77 | WP_003469200.1 | 201D ,  205H | 1uvqB0 | 1.493 | 201D OD2 2.10, O 4.76 |
| 78 | WP_003469256.1 | 32C ,  34G ,  35C | 1a7i_0 | 1.501 | 32C SG, 34G N 4.85, 35C SG 3.16, N 3.96, 77T OG1 3.06, O 2.33, N 4.38 |
| 79 | WP_003469280.1 | 85Q ,  89E | 1jk0A0 | 1.692 | 89E OE2 3.61 |
| 80 | WP_003469507.1 | 257E ,  261E | 1no5B2 | 1.502 | 261E OE2 3.88 |
| 81 | WP_003469598.1 | 262H ,  356E ,  360D | 1kahB0 | 1.857 | 258S OG 4.10, O 4.52, 262H NE2 2.78, 356E OE2 4.11, 360D OD2 |
| 82 | WP_003469599.1 | 19H ,  23D | 1cy5A3 | 1.869 | 19H NE2 2.45, O 4.96 |
| 83 | WP_003469626.1 | 912C ,  915C ,  932C ,  935C | 1dvpA1 | 3.177 | 912C SG 2.31, 915C SG 2.37, N 3.78, 916W N 4.93, 919R NE 4.92, 932C SG, N 3.66, 935C SG 2.63, N 4.24 |
| 84 | WP_003469645.1 | 120E ,  123E | 3caoA3 | 1.656 | 75E OE1 3.18, 203Y OH 2.12, 123E OE1 2.12 |
| 85 | WP_003469868.1 | 308D ,  309D | 1bawA0 | 1.531 | 307R NH1 4.16, N 4.46, 308D N 4.12, 309D OD1 2.59, N 4.15, 311S N 4.89, O 4.86 |
| 86 | WP_003469871.1 | 388D ,  391L | 1taq_0 | 1.412 | 388D OD1 2.51 |
| 87 | WP_003469968.1 | 171E ,  175D | 1a7w_0 | 1.537 | 171E O 3.93, 175D OD2 2.82 |
| 88 | WP_003469990.1 | 531E ,  534E | 1r4vA0 | 1.797 | 531E OE2 2.37, 534E OE1 2.06 |
| 89 | WP_003470030.1 | 90E ,  112D ,  114L | 1cnqA0 | 2.361 | 67D OD2 4.83, 91E OE1 3.67, 112D OD2 2.48, 114L O 2.86, N 3.99, 116G N 4.44 |
| 90 | WP_003470036.1 | 96C ,  99C ,  102C ,  110C | 1njgA0 | 2.729 | 96C SG 2.43, N 3.66, 97G N 3.63, 98H N 4.38, 99C SG 2.35, 102C SG 2.46, N 4.21, 110C SG 2.65 |
| 91 | WP_003470124.1 | 30C ,  210C ,  235H ,  239E | 1li7A0 | 2.646 | 28Y OH 4.79, 30C SG 2.65, 68N OD1 4.88, ND2 4.99, 210C SG, 235H NE2, 239Q 2.81 |
| 92 | WP_003470165.1 | 240E ,  276E ,  304D ,  347D | 1xllA1 | 2.413 | 240E OE2 2.37, 276E OE1, 304D OD2 2.22, 347D OD1 2.03 |
| 93 | WP_003470173.1 | 163E ,  167E | 1no5B2 | 1.739 | 167E OE2 3.30 |
| 94 | WP_003470254.1 | 51E ,  54H ,  127E | 1b71A0 | 2.346 | 18E OE2 2.10, 51E OE1 2.09, 54H ND1 2.44, 94E OE1 4.91, 127E OE2 |
| 95 | WP_003470294.1 | 328H ,  329E | 1k9zA6 | 1.689 | 317H ND1 4.40,329E N 4.87 |
| 96 | WP_003470297.1 | 272E ,  275E | 1r4vA0 | 1.453 | 148D OD2 3.62, 275E OE1 2.06 |
| 97 | WP_003470459.1 | 120H ,  121E | 1k9zA6 | 1.863 | 40R NH1 2.97, 85V O 4.39, 87E N 4.75, 118R CD, NH1 3.03, O 4.38, 121E OE2 1.97, N 4.95, |
| 98 | WP_003470478.1 | 7H ,  9H ,  234D | 1j79A0 | 2.016 | 7H NE2 2.37, 9H NE2, 166H NE2 3.70, 234D OD1 2.40 |
| 99 | WP_003470527.1 | 126H ,  132D | 1lhnA0 | 1.699 | 124S O 4.65, 126H NE2, 132D N 4.83, O 4.54 |
| 100 | WP_003470587.1 | 250E ,  253H | 1xllA0 | 1.476 | 250E OE2 2.38, 252E OE1 2.55, 256R NH1 4.49 |
| 101 | WP_003470615.1 | 124D ,  127D | 1mxdA1 | 1.192 | 124D OD1 3.27, 127D OD2 2 |
| 102 | WP_003470617.1 | 16D ,  17H | 1smlA0 | 1.954 | 14H ND1 4.04, 16D OD2 4.54, 17H NE2 3.11, 104D OD2 2.27 |
| 103 | WP_003470629.1 | 301E ,  304E | 3caoA3 | 1.664 | 304E OE1 2.40 |
| 104 | WP_003470669.1 | 229D ,  230H | 1qh3A1 | 1.489 | 225M O 4.21, 226N ND2 4.35, 229D OD2 1.99, 230H ND1 3.24 |
| 105 | WP_003470782.1 | 71D ,  72E | 1mxdA3 | 1.722 | 70R O 2.47, 72E OE1 4.06, N 4.29 |
| 106 | WP_003470923.1 | 145H ,  146E | 1k9zA6 | 1.774 | 143K NZ 4.86, N 4.18, 146E N 5 |
| 107 | WP_003470942.1 | 137D ,  139H | 1jazA0 | 1.806 | 137D OD2 1.94, 139H N 4.74, 140M N 3.58, 164V O 4.86, 189K NZ 4.15 |
| 108 | WP_003471132.1 | 65E ,  69E | 2a0b_0 | 1.888 | 65E OE2 2.63, 69E OE2, O 4.70 |
| 109 | WP_003471228.1 | 431E ,  432H | 1jm7B1 | 1.658 | 368S OG 4.63, 430V O 4.36, 432H ND1 |
| 110 | WP_003471299.1 | 34H ,  38E | 1i1iP1 | 1.474 | 26Y O, 27T OG1 2.97, 95H ND1 3.83, N 4.55 |
| 111 | WP_003471314.1 | 99D ,  100D | 1bawA0 | 1.884 | 5W N 2.74, O 4.71, 99D OD1 2.39, 100D N 4.26, 101F N 2.95, O 2.20 |
| 112 | WP_003471337.1 | 172E ,  175E | 3caoA3 | 1.698 | 172E OE2 |
| 113 | WP_003471408.1 | 61D ,  131H | 1lbu_0 | 1.493 | 60V O 4.57, 131H ND1 2.05 |
| 114 | WP_003471593.1 | 91H ,  95D | 1hfeS0 | 1.527 | 64R O 3.44, 68D OD1 4.10, 91H O 4.87, 95D OD2 2.59 |
| 115 | WP_003471664.1 | 65D ,  67D | 1f35A3 | 1.499 | 65D OD2, 67D N 4.50, 68T OG1 2.99, N 3.83 |
| 116 | WP_003471666.1 | 58K ,  61R | 1l9hA1 | 1.42 | 58K NZ 3.51, 61R NH1 4.69, 62D OD1 3.71, OD2 4.01, 65E OE1 4.69 |
| 117 | WP_003471682.1 | 229E ,  233E | 2a0b_0 | 1.774 | 226R NH1 4.89, 229E O 4.97, 233 OE1 3.53 |
| 118 | WP_003471820.1 | 42C ,  44D ,  98H ,  101C | 1ddzA1 | 2.998 | 42C SG 2.24, 44D OD2, N 4.37, 67A N 4.74, 98H NE2 2.16, 101C SG, 102G N 4.13, 03G N 4.35 |
| 119 | WP_003471844.1 | 94E ,  239H | 1r3nA1 | 1.813 | 94E OE2 2.19, 239H NE2 2.35, 273Y OH 3.59 |
| 120 | WP_003471850.1 | 68E ,  72Q | 1no5B2 | 1.263 | 72Q NE2 3.03, 136G O 2.59, 139A N 4.20, 151Q NE2 4.23 |
| 121 | WP_003471878.1 | 429D ,  431V ,  458E | 1k9zA1 | 1.542 | 429D OD1 2.02, 430L O 3.33, N 4.21, 456H O 4.88, 457A O 4.72, 458E O 3.79, 460S N, CA, CE |
| 122 | WP_003471965.1 | 82C ,  139C ,  141C ,  146C | 1pegB3 | 2.548 | 80H NE2 4.37, 82C SG, N 4.09, 139C SG 2.50, 141C SG 2.74, 146C SG 2.29, 147S N 3.48, OG 4.41, 148G N 4.16 |
| 123 | WP_003471967.1 | 33E ,  38Q | 1gudA3 | 1.453 | 33E OE2 3.51, 39V N 2.34, O 2.01, 40D OD1 4.70 |
| 124 | WP_003472015.1 | 242E ,  245E | 3caoA3 | 1.667 | 242E OE2 4.84, 257N ND2 2.11, N 4.79 |
| 125 | WP_003472018.1 | 110D ,  113M | 1taq_0 | 1.43 | 110D OD1 2.19 |
| 126 | WP_003472081.1 | 121M ,  122H | 1fioA0 | 1.876 | 110R NH2 4.74, 118D O 2.55, 122H N 2.12, 123D N 3.07, 124A N 4.65 |
| 127 | WP_003472086.1 | 5Q ,  6C | 1xpa_0 | 1.313 | 4R O 4.92, 6C SG 2.92, N 3.33 |
| 128 | WP_003472098.1 | 105H ,  106E | 1k9zA6 | 1.694 | 92R NH1 3.92, NH2 4.30, 106E OE1 5 |
| 129 | WP_003472119.1 | 32E ,  36D | 1a7w_0 | 1.678 | 32E OE2 3.37, O 4.38, 36D OD2 2.02 |
| 130 | WP_003472142.1 | 341H ,  342E | 1k9zA6 | 1.732 | 242R NH2 4.09, 336D OD1 4.98, 337L O, 342E N 4.84 |
| 131 | WP_003472490.1 | 51Q ,  99C ,  141H ,  145H | 1ix1A0 | 2.698 | 51Q 3.87, 98G O 4.13, 99C SG 2.33, 100L N 4.77, 141H NE2 2.12, 142E OE1 4.60, OE2 4.30, 145H NE2 2.38 |
| 132 | WP_003472589.1 | 109H ,  111H | 1odhA0 | 1.519 | 109H ND1 |
| 133 | WP_003472647.1 | 39K ,  42R | 1l9hA1 | 1.535 | 43E OE2 4.06 |
| 134 | WP_003472648.1 | 20D ,  21D | 1bawA0 | 1.714 | 20D OD1 2.22, 21D OD2 3.32, N 4.28, 22S N 3.55, O 4.40, 64D OD1 3.97, O 4.28, 66M N 3.66, 116K NZ |
| 135 | WP_003472675.1 | 107E ,  110E | 3caoA3 | 1.757 | 107E OE1 3.35, 110E OE1 2.17 |
| 136 | WP_003472685.1 | 158E ,  162D | 1a7w_0 | 1.74 | 158E OE2 3.71, O 4.35 |
| 137 | WP_003472695.1 | 78H ,  112C ,  115C | 1rb7A0 | 2.509 | 78H ND 2.14, 80E OE2 3.92, 112C SG 2.39, 115C SG, N 3.83 |
| 138 | WP_003472750.1 | 13E ,  25E | 1l0iA5 | 1.401 | 12R O 4.98, 13E OE2 4.36, 24E OE1 3.39, 40R O 4.11, 42T OG1 2.44, N 4.25 |
| 139 | WP_003472753.1 | 89D ,  92D ,  214D | 1k9zA2 | 1.486 | 89D OD2 1.92, 91L O 4.50, 92D OD1 2.20, 93G N 4.64, 213W NE1 4.59, 214D OD2 2.28 |
| 140 | WP_003472896.1 | 83E ,  86Q | 3caoA3 | 1.161 | 86Q OE1 2.42, NE2 2.72 |
| 141 | WP_003472954.1 | 202D ,  203H | 1qh3A1 | 1.851 | 202D OD2 2.39 |
| 142 | WP_003473108.1 | 154D ,  158E | 1f30A0 | 1.465 | 158E OE2 3.05 |
| 143 | WP_003473181.1 | 57R ,  58C | 1i7wC0 | 1.724 | 54E SG 3.11, O 4.82, 58C SG 3.11, 61R NH1 3.67, 79Q NE2 4.62 |
| 144 | WP_003473187.1 | 464H ,  468H ,  494E | 1i1iP0 | 2.11 | 464H NE2 2.43, 465E OE1 4.86, 468H NE2 2.13, 494E OE1, 497S OG 3.57, 601Y OH 4.30, 60Y OH 4.48 |
| 145 | WP_003473476.1 | 10E ,  13E | 3caoA3 | 1.695 | 13E OE1 2.35 |
| 146 | WP_003476601.1 | 186E ,  220E ,  223H | 1mftA0 | 2.291 | 89D OD1 4.51, 119E OE2 2.55, 122H ND1 4.48, 186E OE2 2.28, 220E OE1 2.00, 223H ND1 1.95 |
| 147 | WP_003477416.1 | 14C ,  16V ,  17C ,  19F ,  47C ,  50C ,  52V | 1dx8A0 | 2.884 | 14C SG 2.40, 17C SG 2.66, N 4.55, 47C SG 2.41, 49D OD2 4.22, 50C SG, N 4.30, 52V N 4.63 |
| 148 | WP_003477578.1 | 111D ,  206E | 1vsh_0 | 1.426 | 111D OD1 2.98, 206E OE2 2.55 |
| 149 | WP_003481247.1 | 97D ,  98H | 1smlA0 | 1.793 | 97D OD2 |
| 150 | WP_003481607.1 | 26E ,  29E | 1r4vA0 | 1.457 | 29E OE1 2.10 |
| 151 | WP_003488188.1 | 30H ,  60Q | 1hp7A0 | 0.932 | 57K NZ 3.64, 58G O 4.94 |
| 152 | WP_004425452.1 | 198T ,  199H | 1qe3A0 | 1.394 | 44K NZ 4.96, 197I O 4.19, 198T OG1, 199H N 2.22, 200E OE1 3.78, O 4.92, N 3.74 |
| 153 | WP_004425502.1 | 151E ,  221H ,  242H | 1itqA1 | 2.174 | 46H NE2 3.63, 151E OE2 2.33, 178H NE2 4.60, 221H NE2 2.52, 242H NE2, 253R NH2 3.48, NE 4.79, 275Y OH 3.67, 355D OD1 4.07 |
| 154 | WP_004425521.1 | 46E ,  49D | 3caoA3 | 1.254 | 49D OD2 3.87 |
| 155 | WP_004425530.1 | 486D ,  490E | 1f30A0 | 1.394 | 170R NH2 3.92, 505G O 4.49 |
| 156 | WP_004425664.1 | 57E ,  60E | 1r4vA0 | 1.497 | 45E OE2 4.31, 56R NH1 3.22, 57E OE2 2.19, 60E OE1 3.15 |
| 157 | WP_004425670.1 | 403D ,  407E | 1f30A0 | 1.714 | 395R NH1 4.70, NH2 4.74, 403D OD2 4.20, 407E OE1 3.29 |
| 158 | WP_004425677.1 | 30H ,  31E | 1k9zA6 | 1.389 | 25K NZ 3.61, 29G O 3.07 |
| 159 | WP_004425727.1 | 19E ,  23H | 1lbcB1 | 1.825 | 19E O 4.99, 23H NE2 2.74 |
| 160 | WP_004425920.1 | 506H ,  510H ,  535E | 1i1iP0 | 2.502 | 506H NE2, 507E OE1 4.85, 510H NE2 2.20, 535E OE1 2.14, 538S OG 3.57, 644Y OH 4.79, 651Y OH 4.33 |
| 161 | WP_004426268.1 | 195H ,  196E | 1k9zA6 | 1.881 | 127K NZ 4.84, 196E N 4.91, 200D OD1 2.82 |
| 162 | WP_004426362.1 | 209H ,  210C ,  234C ,  245N ,  246C | 1b8tA0 | 1.769 | 208T O 4.16, 210C SG 2.69, O 2.92, N 2.38, 233E O 2.64, N 4.83, 234C SG 3.34, 246C N 3.83 |
| 163 | WP_004426406.1 | 198E ,  202E | 2a0b_0 | 1.429 | 198E OE2 3.21, O 4.57, 202E OE2 2.65 |
| 164 | WP_004426468.1 | 49Q ,  53E | 1jk0A0 | 1.676 | 49Q NE2 2.62,53E OE2 2.53 |
| 165 | WP_004426584.1 | 186E ,  189E | 3caoA3 | 1.679 | 189E OE1 2.34 |
| 166 | WP_038236501.1 | 86H ,  90H ,  168E | 1hr6D0 | 1.805 | 86H NE2 2.32, 90H NE2 2.17, 168E OE1 2.14 |
| 167 | WP_038236644.1 | 305C ,  307C ,  310C ,  337H | 1q2rA0 | 2.954 | 305C SG 2.24, 307C SG 2.26, 310C SG 2.41, N 4.01, 337H ND1 2.19, O 4.62 |
| 168 | WP_038237017.1 | 137H ,  167D ,  174H | 1hp7A0 | 1.543 | 137H ND1 3.78, 167D OD2 2.80 |
| 169 | WP_038237239.1 | 287H ,  291D | 1cy5A3 | 1.497 | 287H NE2, 291D OD1 4.11 |
| 170 | WP_038238095.1 | 207D ,  209V | 1dk4A1 | 1.587 | 207D OD1 2.58, 209V N 4.25, 235R NH1 4.41, NH2 4.61, CG |
| 171 | WP_038238334.1 | 248E ,  252D | 1a7w_0 | 1.826 | 248E OE2 3.45, O 4.46, 252D OD2 2.86 |
| 172 | WP_038238529.1 | 336H ,  337E | 1k9zA6 | 1.809 | 330C SG 3.09, 333C SG 3.09, 408I O 4.87, 409A 3.77, 412I N 4.39 |
| 173 | WP_038238706.1 | 107H ,  115H ,  124H ,  127D | 1eqwB0 | 2.866 | 107H ND1 2.11, 115H ND1 1.96, 116G O 4.90, N 4.65, 124H ND1 2.24, N 4.72, 127 D OD1 1.90, 170D OD2 2.77, 176P O 3.86 |
| 174 | WP_038238771.1 | 97C ,  100C ,  103C ,  111C | 1kolA1 | 2.952 | 97C SG 2.28, N 3.91, 98G N 3.82, 99H N 4.49, 100C SG, N 3.62, 103C SG 2.86, N 4.17, 111C SG 2.63 |
| 175 | WP_038238963.1 | 132C ,  135C ,  150C ,  153C | 1vk6A0 | 3.235 | 132C SG 2.61, 135C SG 2.37, N 4.01, 136G N 4.85, 150C SG 2.31, 153C SG N 3.57 |
| 176 | WP_038239202.1 | 56H ,  140E | 1iwlA1 | 1.337 | 140E OE1 3.53 |
| 177 | WP_038239399.1 | 359H ,  363D | 1ak0_1 | 1.78 | 359H NE2 2.19 |
| 178 | WP_047324411.1 | 88H ,  92H ,  170E | 1hr6D0 | 1.8 | 88H NE2 2.32, 92H NE2 2.17, 170E OE1 2.14 |
| 179 | WP_047324417.1 | 163E ,  167D | 1a7w_0 | 1.836 | 163E O 4.38, 167D OD2 |
| 180 | WP_047324427.1 | 502Q ,  504H | 1l0yA0 | 1.515 | 493T OG1 3.87, 502Q OE1 3.63, 504H NE2 3.46 |
| 181 | WP_047324433.1 | 252E ,  255E | 1r4vA0 | 1.39 | 252 OE1 3.93, OE2 4.27, 255E OE1 2.11, 261R NE 3 NH2 4.25 |
| 182 | WP_047324452.1 | 23C ,  233C | 1q08A0 | 1.329 | 23C SG 3.26, 229F O 4.86, 230G O 3.70, 237C SG 4.82 |
| 183 | WP_047324462.1 | 557D ,  559D ,  561D | 1k2yX0 | 2.606 | 431K NZ 3.82, 557D OD2 1.96, 559D OD2 2.07, N 4.45, 560A N 4.60, 561D OD1 2.03, N 4.21, 562R NE 4.2, N 4.55, 644H NE2 4.44 |
| 184 | WP_047324470.1 | 94C ,  97C ,  100C , 108C | 1jr3E0 | 2.674 | 93S OG 4.60, 94C SG 2.53, N 3.58, 95R N 3.71, 96H N 4.51, 97C SG 2.56, N 4.08, 100C SG, N 4.12, 108C SG 2.31 |
| 185 | WP_047324475.1 | 231D ,  233D | 1no5B0 | 1.373 | 46T OG1 3.54, 47Q N 1.90, OE1 4.83, 48S N 4.67, 231D N 4.67, 233D OD2 2.12 |
| 186 | WP_047324485.1 | 61E ,  65E | 1q9uB4 | 1.672 | 61E O 4.63, 65E OE1 |
| 187 | WP_047324501.1 | 54Q ,  94C ,  136H , 140H | 1ix1A0 | 3.696 | 54Q NE2 3.45, O 4.72, 93G O 4.72, 94C SG 2.32, 95L N 4.20, 96S N 4.82, 136H NE2 2.23, 137E OE1 4.59, OE2 4.33, 140H NE2 2.08 |
| 188 | WP_047324520.1 | 8D ,  9D | 1bawA0 | 1.795 | 8D OD1 2.15, 9D OD1 2.67, N 4.24, 10D N 3.94 |
| 189 | WP_047324534.1 | 129E ,  132E | 1r4vA0 | 1.528 | 132E OE1 2.11, 328D O 4.23, 330I N 4.16 |
| 190 | WP_047324537.1 | 305C ,  307C ,  310C , 337H | 1q2rA0 | 2.954 | 305C SG 2.24, 307C SG 2.26, N 3.82, 310C SG 2.41, N 4.01, 337H ND1 2.19, O 4.62 |
| 191 | WP_047324567.1 | 157D ,  159H ,  240D | 1c3rA0 | 2.098 | 119H NE2 4.61, 120H NE2 4.81, 157D OD2 2, 158V N 4.07, 159H ND1, N 3.31, 240D OD2 2.21 |
| 192 | WP_047324573.1 | 214E ,  218D | 1a7w_0 | 1.827 | 214E O 4.27, 218D OD2 |
| 193 | WP_047324579.1 | 142D ,  143D | 1bawA0 | 1.357 | 140M O 4.68, 142D OD1 3.71, N 4.17, 143D N 4.15, O 4.64, |
| 194 | WP_047324585.1 | 323H ,  327E | 1i1iP1 | 1.466 | 10L O 4.38, 12I N 3.28, 51R NH1 2.53 NH2, 327E OE2 |
| 195 | WP_047324608.1 | 380D ,  381D | 1bawA0 | 1.523 | 378A O 3.66, 381D N 4.15, 384I N 3.91, O, 385H ND1 4.23, |
| 196 | WP_047324609.1 | 336H ,  340H ,  359E | 1fjtA0 | 2.091 | 303E OE1 2.23, 336H NE2 2.69, 337E OE1 4.16, OE2 4.03, 340H NE2 2.03, 359E OE1 2.76 422Y OH 4.36 |
| 197 | WP_047324617.1 | 148C ,  151C ,  155G , 284C ,  286T ,  287C | 1exkA1 | 3.735 | 148C SG 2.31, 150T OG1, 151C SG 2.30, N 3.45, 152H N 4.89, 155G 4.92, 284C SG 2.33, 287C SG 2.71, N 4.12, 290K O 4.93 |
| 198 | WP_047324619.1 | 90C ,  93C ,  96C ,  104C | 1njgA0 | 3.099 | 90C SG 2.50, N 3.59, 91G 3.97, 92E N 4.43, 93C SG, N 3.83, 96C SG 2.39, N 4.30, 104C SG 2.30, 105E N 3.94, 106T OG1 3.36 |
| 199 | WP_047324635.1 | 111D ,  206E | 1vsh_0 | 1.425 | 111D OD1 2.98, 206E OE2 2.55 |
| 200 | WP_047324647.1 | 373C ,  393C ,  396C | 1e3eA1 | 1.922 | 373C SG, 375R N 4.53, 393C SG 2.31, N 4.15, 396C SG 2.41, N 4.42 |
| 201 | WP_047324648.1 | 94D ,  98E | 1f30A0 | 1.683 | 98E OE2 2.80 |
| 202 | WP_047324660.1 | 557H ,  559D | 1h1zA0 | 1.432 | 559D OD1 2.55 |
| 203 | WP_047324671.1 | 100D ,  102H | 1ei6A1 | 1.395 | 22G O 2.40, 24R NH1 3.03, N 4.20, 100D OD2 4.32 |
| 204 | WP_047324673.1 | 240Q ,  244E | 1jk0A0 | 1.748 | 210K NZ 4.06, 240Q OE1 4.94, 244E OE2 2.01 |
| 205 | WP_047324677.1 | 305E ,  308E | 1q9uB4 | 1.241 | 305E O 4.27, 306Q NE2 4.49, 309H ND1 4, N 4.22 |
| 206 | WP_047324684.1 | 40D ,  44E | 1f30A0 | 1.462 | 12H N 4.12, 44E OE1 1.93 |
| 207 | WP_047324687.1 | 353E ,  356E | 3caoA3 | 1.702 | 353E OE2, 356E OE2 4.08 |
| 208 | WP_047324691.1 | 337H ,  341D | 1cy5A3 | 1.536 | 341D OD1 2.68 |
| 209 | WP_047324698.1 | 51E ,  55H | 1m4mA0 | 1.531 | 2L N 4.93, 5T OG1 4.86 |
| 210 | WP_047324715.1 | 168T ,  228C ,  294C ,  295C | 1umyD0 | 2.114 | 228C SG 3.30, N 4.83, 254N OD1 3.12, ND2 4.85, 294C SG 2.36, 295C SG 2.52, N 2.70 |
| 211 | WP_047324717.1 | 121E ,  124E | 3caoA3 | 1.695 | 124E OE1 2.79, 153L O 3.76, 157L N 4.06 |
| 212 | WP_047324718.1 | 843E ,  846E | 1r4vA0 | 1.946 | 846E OE1 2.24 |
| 213 | WP_047324738.1 | 98H ,  99E | 1k9zA6 | 1.828 | 95R N 4.82, 99E OE1 4.32, N 4.86 |
| 214 | WP_047324744.1 | 374E ,  378D | 1a7w_0 | 1.825 | 374E OE2 3.29, O 4.28, 378D OD2 1.96 |
| 215 | WP_047324745.1 | 538H ,  542H ,  601E | 1dmtA0 | 2.486 | 538H NE2 2.03, 539E OE2 4.94, 542H NE2 2.08, 601E OE1 1.93, 661D NE2 3.81 |
| 216 | WP_047324753.1 | 245D ,  247D ,  249D | 1kfiA0 | 2.049 | 106S OG, 107H ND1 4.86, N 3.73, 116K NZ 4.82, 245D OD2 2.59, 247D OD1 2.23, N 4.41, 249D OD2 2.56 |
| 217 | WP_047324755.1 | 104T ,  105H | 1qe3A0 | 1.503 | 100R NE 4.95, 101D OD1 3.16, O, 104T OG1 4.20, 105H N 2.25,3.26, 106V N 3.70 |
| 218 | WP_047324767.1 | 255D ,  266D ,  268T , 378E | 1pv9A1 | 3.222 | 255D OD1 2.10, 266D OD1, 268T OG1 3.46, 364E OE1 4.48, 376R NH2 4.78, NE 4.98, 378E OE1 2.15 |
| 219 | WP_047324776.1 | 73H ,  75H ,  310D | 1j79A0 | 1.818 | 73H NE2 2.22, 75H NE2, 133N ND2 4.80, 222H NE2 2.42, 259H NE2 4.82, 310D OD1 2.72 |
| 220 | WP_047324777.1 | 29D ,  31D | 1f35A3 | 1.527 | 31D OD2 4.55, N 4.50 |
| 221 | WP_047324781.1 | 205M ,  206H | 1fioA0 | 1.898 | 202T O 3.52, 203P O, 204D OD1 4.65, 206H N 1.95 |
| 222 | WP_047324797.1 | 80T ,  84E | 7mdhB4 | 1.253 | 2I O 4.69, 4T OG1 4.70, 80T OG1 4.96, 84E OE2 3 |
| 223 | WP_047324805.1 | 232H ,  236D | 1ak0_1 | 1.943 | 175R NH1 3.49, 177T OG1, N 4.15, 179E OE1 3.67, N 4.73, 180T OG1 3.44, N 4.10, O 4.53, 236D OD2 2.34 |
| 224 | WP_047324810.1 | 334C ,  385H ,  511H | 1kogA0 | 2.218 | 334C SG 2.38, N 4.55, 383D OD2 4.09, 385H NE2 2.23, 484Q OE1 4.83, 511H ND1 2.14 |
| 225 | WP_047324842.1 | 24A ,  28C | 1eucB0 | 1.785 | 10L O 4.30, 24A O 3.24, 28C SG |
| 226 | WP_047324869.1 | 295D ,  299E | 1f30A0 | 1.799 | 298R NH2 4.23, 299E OE1 4.43 |
| 227 | WP_047324934.1 | 44C ,  46E ,  47C ,  112Y | 1bbo_0 | 1.596 | 43E O 4.50, 44C O 4.69, 47C SG 2.42, N 4.11, 46E OE1 4.03, N 4.99, 111K O 4.71 |
| 228 | WP_047324950.1 | 64H ,  65E | 258lA0 | 1.87 | 13L O 4.57, 14Q NE2 3.79, 16K N 4.62, 39G O 2.79 |
| 229 | WP_047324951.1 | 220H ,  221D | 1k9zA6 | 1.354 | 218T OG1 2.19, N 4.03, 221D OD2 2.74, N 4.94 |
| 230 | WP_047324952.1 | 64D ,  65D | 1bawA0 | 1.799 | 64D OD1 4.57, 65D O 3.05, N 4.26, 90R NH2 4.70, NH1 4.89, NE 2.85, N 3.73, CG |
| 231 | WP_047324961.1 | 11D ,  12D | 1bawA0 | 1.913 | 11D OD2 2.33, 12D OD1 2.46, N 4.14, 13F N 4.15, 56D OD1 3.29, 58N O 3.62, 108K NZ 4.99 |
| 232 | WP_047324979.1 | 374E ,  377E | 3caoA3 | 1.805 | 377E OE1 2.91 |
| 233 | WP_047324986.1 | 570H ,  574H ,  672C | 1lqwA0 | 1.606 | 574H NE2 2.55, 672C SG, N, CB, 676H NE2 3.87 |
| 234 | WP_047325000.1 | 235D ,  236D | 1bawA0 | 1.948 | 235D OD1 2.51, 236D O 3.05, N 4.28 |
| 235 | WP_047325029.1 | 54C ,  56G ,  57C | 1exkA1 | 1.51 | 53Y O 3.03, 54C O 3.92, 56G N 4.66, 57C SG 3.30 |
| 236 | WP_047325048.1 | 184M ,  185H | 1fioA0 | 1.859 | 181A O 3.39, 182A O, 185H ND1 2.76, N 1.91, 186D N 4.31 |
| 237 | WP_047325053.1 | 62K ,  65R | 1l9hA1 | 1.719 | 66R NH2 4.02, NE 3.98 |
| 238 | WP_047325063.1 | 18E ,  51E ,  54H | 1mftA0 | 1.915 | 18E OE1 2.32, 50E O 4.64, 51E OE1 2.01, 54H ND1, 122Q OE1 4.14, 126T OG1 4.70, 130H ND1 4.54 |
| 239 | WP_047325067.1 | 66H ,  99D ,  133E ,  162E | 1cg2A1 | 2.462 | 66H NE2 2.12, 99D OD1 2.54, 133E OE1 2.73, 134E OE2 3.60, 162E OE1 2.72, OE2 2.63 |
| 240 | WP_047325082.1 | 37D ,  41E | 1f30A0 | 1.505 | 41E OE2 2.38, 68Y OH 4.97 |
| 241 | WP_047325089.1 | 90C ,  93C ,  96C ,  104C | 1kolA1 | 2.847 | 90C SG 2.52, N 3.88, 91G N 3.87, 92R N 4.48, 93C SG, N 4.05, 95E OE1 4.93, 96C SG 2.50, N 4.17, 104C SG 2.60 |
| 242 | WP_047325103.1 | 515S ,  517H | 1iqbA0 | 1.5 | 516Q N 3.37, 518W NE1 4.88, 595W O 4, 596E O 4.23, 608Y OH 4.68 |
| 243 | WP_047325123.1 | 52E ,  56E | 2a0b_0 | 1.723 | 40R NH2 3.92, 52E OE1 4.65, O 4.70, 56E OE1 3.27 |
| 244 | WP_047325129.1 | 130E ,  134D | 1kahB0 | 1.512 | 130E OE2 4.18, O 4.72, 134D OD2 |
| 245 | WP_047325137.1 | 530H ,  533H | 1fioA1 | 1.47 | 529W O 4.74, 530H ND1 2.55 |
| 246 | WP_047325171.1 | 14D ,  118D ,  121I ,  141D | 1taq_0 | 2.292 | 14D OD2 3.04, 118D OD1 3.07, 139S OG 4.63, 141D OD1 2.31 |
| 247 | WP_047325199.1 | 228H ,  240D ,  274E ,  302D | 1cg2A1 | 2.224 | 228H NE2 2.01, 240D OD1 2.40, 274E OE1 3.42, 275E OE1 4.24, 302D, 2.12, O 4.51, 370Y OH 4.35 |
| 248 | WP_047325217.1 | 69D ,  86H ,  139H ,  143D | 1ak0_0 | 2.982 | 30H NE2 4.59, 69D OD1 2.57, 86H ND1 1.99, 139H NE2 2.01, 143D OD2 2.05 |
| 249 | WP_047325218.1 | 115D ,  116D | 1bawA0 | 1.838 | 116D OD1 3.36, N 4.22 |
| 250 | WP_047325232.1 | 262D ,  273D ,  275T ,  409E | 1pv9A1 | 3.199 | 231Y OH 3.61, 262D OD1 1.99, 273D OD1 2.04, 275T OG1 3.47, 385E OE1 4.12, 407R NH2 4.92, NE 4.77, 409E OE1 2.10 |
| 251 | WP_047325244.1 | 309E ,  312E | 1r4vA0 | 1.879 | 308H NE2 3.98, 309E OE1 3.18 |
| 252 | WP_047325261.1 | 82D ,  83D | 1bawA0 | 1.326 | 83D OD1 2.12, N 3.94, 106F O 3.99, 132H OD2, 133S O 4.11 |
| 253 | WP_047325264.1 | 266H ,  270H | 2psr_0 | 1.481 | 141I O 3.51, 142S OG 4.14, 166S OG, 266H ND1 4.09, 270H NE2 |
| 254 | WP_047325265.1 | 92C ,  95C ,  98C ,  106C | 1kolA1 | 3.164 | 92C SG 2.42, N 3.50, 93R N 3.86, 94K N 4.52, 95C SG 2.33, N 3.83, 98C SG 2.25, N 4.17, 106C SG 2.28, 107Q N 4.15, 108A N 4.52 |
| 255 | WP_047325269.1 | 250D ,  251D | 1bawA0 | 1.454 | 251D OD1 2.90, N 3.93 |
| 256 | WP_047325270.1 | 67E ,  71E | 1q9uB4 | 1.678 | 67E O 4.75, 71E OE1 2.12, 75R NH2 4.99, 115H ND1 3.19 |
| 257 | WP_047325273.1 | 35D ,  36D | 1bawA0 | 1.897 | 7R NH2 3.69, 20G O 2.69, 35D OD1 2.76, 36D N 4.15, 37G N 4.65 |
| 258 | WP_047325324.1 | 312D ,  314D | 1f35A3 | 1.738 | 161R NE 3.47, NH2, 312D OD1 2.01, |
| 259 | WP_047325420.1 | 87V ,  88H | 1fioA0 | 1.187 | 88H N 1.95, 89Q OE1 3.29, NE2 4.35, N 3.62, 102R NH1 4.72 |
| 260 | WP_047325424.1 | 499D ,  501D | 1bawA0 | 1.876 | 499D OD1 2.40, 501D O 4.45, N 4.16, 504Q OE1 2.54 |
| 261 | WP_047325436.1 | 342D ,  376E ,  495H | 1f2oA1 | 2.315 | 320H NE2 4.61, 342D OD2 2.02, 375E OE1 4.39, 376E OE2, 403E OE2 4.64, 494Y OH 4.27, 495H NE2 2.06 |
| 262 | WP_047325465.1 | 242H ,  293D | 1dvfD0 | 1.693 | 241R O 4.48, 293D OD2, 301K NZ 4.75 |
| 263 | WP_047325488.1 | 756D ,  757D | 1bawA0 | 1.8 | 217A O 3.22, N 2.91, 667D OD2 2.96, 757D O 3.08, N 4.19 |
| 264 | WP_047325492.1 | 240E ,  276E ,  304D ,  347D | 1xllA1 | 2.409 | 240E OE2 2.37, 276E OE1, 304D OD1 2.22, 347D OD1 2.03 |
| 265 | WP_047325525.1 | 53Q ,  57D | 1a7w_0 | 1.233 | 53Q O 4.27, 57D OD2 |
| 266 | WP_047325527.1 | 373E ,  377E | 1no5B2 | 1.537 | 377E OE2 2.46, 384P O 4.92 |
| 267 | WP_047325529.1 | 209E ,  213E | 1no5B2 | 1.527 | 209E OE2 2.37, 213E OE2 4.12 |
| 268 | WP_047325583.1 | 180Q ,  184E | 1jk0A0 | 1.391 | 180Q NE2 2.59, 184E OE2 2.28 |
| 269 | WP_047325628.1 | 241D ,  244E | 1r4vA0 | 1.323 | 241D OD1 3.22, 256L O 2.00, 258R NH1 3.98, N, 259N N 4.38 |
| 270 | WP_047325669.1 | 30C ,  35V ,  96C ,  99C ,  101V | 1irn_0 | 1.892 | 28C SG, 29H N 4.05, 30C SG 2.44, N 3.62, 35V N 4.58, 78H NE2 4.63, 81K NZ 3.74, 96C 2.63 SG 2.36, 99C SG 2.63 |
| 271 | WP_047325670.1 | 174H ,  175E | 1k9zA6 | 1.697 | 127Q NE2 4.15, 171E O 4.35, 175E N 4.82 |
| 272 | WP_047325674.1 | 8E ,  11E | 3caoA3 | 1.779 | 8E OE2 2.29, 11E OE1 2.97 |
| 273 | WP_047325709.1 | 153D ,  154D | 1bawA0 | 1.779 | 136N ND2 3.75, 154D OD1 2.89, N 4.17 |
| 274 | WP_053008585.1 | 190H ,  191R ,  193C | 1hbmD0 | 1.641 | 190H O 3.42, 191R O 2.91,196L N 4.65, 235Y OH 4.49 |
| 275 | WP_053008603.1 | 27G ,  28H ,  94C ,  99H ,  103M | 1e67A0 | 3.061 | 27G O 3.09, 28H ND1 2.05, 29N N 4.85, 94C SG 2.85, 99H ND1 2.09, 103M SD 3.05 |
| 276 | WP_080627636.1 | 111D ,  174H ,  207E ,  238E | 1pv9A0 | 2.753 | 111D OD1 2.12, 174H NE2 2.91, 180Y OH 4.26, 205T OG1 4.01, 207E OE2 2.59, 238E OE2 |
| 277 | WP_080964784.1 | 302E ,  306H | 1m4mA0 | 1.9 | 306H NE2 2.88, 312Y N 4.90 |
| 278 | WP_080964790.1 | 177H ,  178D | 1udtA0 | 1.481 | 177H NE2 4.81, 178 OD2 2.16 |
| 279 | WP_080964854.1 | 192E ,  199D | 1cnqA0 | 1.46 | 192E OE2 2.94, 199D OD2 3.24, N 4.95 |

**Supplementary material, table S3: Functional classification of Zn-binding proteins of *Xtu***

| **S. No** | **Sequence Id of the putative Zn-binding protein** | **Subcellular localization of putative Zn-binding protein** | **Predicted functional domain/family in putative Zn-binding proteins** | **Broad functional class of putative Zn-binding protein based on literature review of predicted domain/family** | **References** |
| --- | --- | --- | --- | --- | --- |
| 1 | WP_003471314.1 | Cytoplasmic | 2-C-methyl-D-erythritol 4-phosphate cytidylyltransferase | Metabolic process | [1] |
| 2 | WP_004426362.1 | Cytoplasmic | 2-isopropylmalate synthase, bacterial-type | Metabolic process | [2] |
| 3 | WP_047325674.1 | Cytoplasmic | 3,4-dihydroxy-2-butanone 4-phosphate synthase | Metabolic process | [3] |
| 4 | WP_003468120.1 | Cytoplasmic | 3-beta hydroxysteroid dehydrogenase/isomerase family | Metabolic process | [4] |
| 5 | WP_003471682.1 | Cytoplasmic | 3-dehydroquinate synthase | Metabolic process | [5] |
| 6 | WP_003468654.1 | Cytoplasmic | 3-hydroxyacyl-CoA dehydrogenase, NAD binding domain | Metabolic process | [6] |
| 7 | WP_047324684.1 | Cytoplasmic | 3-hydroxyacyl-CoA dehydrogenase, NAD binding domain | Metabolic process | [6] |
| 8 | WP_003468634.1 | Cytoplasmic | 50S ribosome-binding GTPase | Protein biosynthesis | [7] |
| 9 | WP_004426406.1 | Cytoplasmic | 50S ribosome-binding GTPase | Protein biosynthesis | [7] |
| 10 | WP_047325171.1 | Cytoplasmic | 5'-3' exonuclease | DNA repair | [8] |
| 11 | WP_047324534.1 | Cytoplasmic | AAA domain (Cdc48 subfamily) | Proteolysis | [9] |
| 12 | WP_003466285.1 | InnerMembrane | ABC transporter | Transport | [10,11] |
| 13 | WP_003466345.1 | InnerMembrane | ABC transporter | Transport | [10,11] |
| 14 | WP_003468030.1 | InnerMembrane | ABC transporter | Transport | [10,11] |
| 15 | WP_004425452.1 | InnerMembrane | ABC transporter | Transport | [10,11] |
| 16 | WP_038238334.1 | InnerMembrane | ABC transporter | Transport | [10,11] |
| 17 | WP_047325000.1 | InnerMembrane | ABC transporter | Transport | [10,11] |
| 18 | WP_003468393.1 | Cytoplasmic | Acetyl co-enzyme A carboxylase carboxyltransferase alpha subunit | Metabolic process | [12] |
| 19 | WP_003467331.1 | Cytoplasmic | Adenylate kinase | Metabolic process | [13] |
| 20 | WP_003470297.1 | Cytoplasmic | Adenylylsulphate kinase | Metabolic process | [14] |
| 21 | WP_003470036.1 | Cytoplasmic | Alcohol dehydrogenase GroES-like domain | Metabolic process | [15–17] |
| 22 | WP_038237239.1 | Cytoplasmic | Alcohol dehydrogenase GroES-like domain | Metabolic process | [15–17] |
| 23 | WP_038238095.1 | Cytoplasmic | Alcohol dehydrogenase GroES-like domain | Metabolic process | [15–17] |
| 24 | WP_038238771.1 | Cytoplasmic | Alcohol dehydrogenase GroES-like domain | Metabolic process | [15–17] |
| 25 | WP_047324470.1 | Cytoplasmic | Alcohol dehydrogenase GroES-like domain | Metabolic process | [15–17] |
| 26 | WP_047324585.1 | Cytoplasmic | Alcohol dehydrogenase GroES-like domain | Metabolic process | [15–17] |
| 27 | WP_047324619.1 | Cytoplasmic | Alcohol dehydrogenase GroES-like domain | Metabolic process | [15–17] |
| 28 | WP_047325089.1 | Cytoplasmic | Alcohol dehydrogenase GroES-like domain | Metabolic process | [15–17] |
| 29 | WP_047325265.1 | Cytoplasmic | Alcohol dehydrogenase GroES-like domain | Metabolic process | [15–17] |
| 30 | WP_003465707.1 | Cytoplasmic | Aldo/keto reductase family | Metabolic process | [18] |
| 31 | WP_003470587.1 | Cytoplasmic | Aldo/keto reductase family | Metabolic process | [18] |
| 32 | WP_003472081.1 | Periplasmic | Alpha/beta hydrolase fold | Metabolic process | [19] |
| 33 | WP_003465267.1 | Cytoplasmic | Aminotransferase class I and II | Metabolic process | [20] |
| 34 | WP_003465899.1 | Cytoplasmic | Aminotransferase class I and II | Metabolic process | [20] |
| 35 | WP_003469599.1 | Cytoplasmic | Aminotransferase class I and II | Metabolic process | [20] |
| 36 | WP_038238529.1 | Cytoplasmic | Aminotransferase class I and II | Metabolic process | [20] |
| 37 | WP_047324869.1 | Cytoplasmic | Aminotransferase class I and II | Metabolic process | [20] |
| 38 | WP_053008585.1 | Cytoplasmic | Aminotransferase class I and II | Metabolic process | [20] |
| 39 | WP_047324647.1 | Cytoplasmic | ATP-dependent DNA helicase RecQ, zinc-binding domain | DNA repair | [21] |
| 40 | WP_047325527.1 | Cytoplasmic | ATP-dependent RNA helicase RhlB | RNA processing | [22] |
| 41 | WP_003466374.1 | Cytoplasmic | ATP-grasp fold, succinyl-CoA synthetase-type | Metabolic process | [23] |
| 42 | WP_038239399.1 | Periplasmic | Bacterial extracellular solute-binding protein | Transport | [24] |
| 43 | WP_003470173.1 | Cytoplasmic | Bacterial transferase hexapeptide (six repeats) | Metabolic process | [25] |
| 44 | WP_003470254.1 | Cytoplasmic | Bacterioferritin | Response to oxidative stress | [26,27] |
| 45 | WP_047325063.1 | Cytoplasmic | Bacterioferritin | Response to oxidative stress | [26,27] |
| 46 | WP_003466936.1 | Periplasmic | Beta-lactamase | Antimicrobial resistance | [28] |
| 47 | WP_047324573.1 | Periplasmic | Beta-lactamase | Antimicrobial resistance | [28] |
| 48 | WP_047325082.1 | Periplasmic | Beta-lactamase | Antimicrobial resistance | [28] |
| 49 | WP_003467261.1 | Cytoplasmic | Carbonic anhydrase | Metabolic process | [29] |
| 50 | WP_003471820.1 | Cytoplasmic | Carbonic anhydrase | Metabolic process | [29] |
| 51 | WP_003467454.1 | Cytoplasmic | Carboxyl transferase domain | Metabolic process | [30] |
| 52 | WP_003470459.1 | Cytoplasmic | Carboxyl transferase domain | Metabolic process | [30] |
| 53 | WP_004425530.1 | Periplasmic | Carboxylesterase family | Metabolic process | [31,32] |
| 54 | WP_003465766.1 | Extracellular | Cellulose-binding family II/chitobiase, carbohydrate-binding domain | Metabolic process | [33] |
| 55 | WP_004425670.1 | Cytoplasmic | Clp ATPase | Proteolysis | [34] |
| 56 | WP_003465615.1 | Cytoplasmic | CobQ/CobB/MinD/ParA nucleotide binding domain | Metabolic process | [35] |
| 57 | WP_003467936.1 | Cytoplasmic | 'Cold-shock' DNA-binding domain | Transcription regulation | [36] |
| 58 | WP_003472086.1 | Cytoplasmic | 'Cold-shock' DNA-binding domain | Transcription regulation | [36] |
| 59 | WP_003488188.1 | Cytoplasmic | 'Cold-shock' DNA-binding domain | Transcription regulation | [36] |
| 60 | WP_003466231.1 | Periplasmic | CopA/Muticopper oxidase | Response to oxidative stress | [37] |
| 61 | WP_003470527.1 | Periplasmic | Copper/zinc superoxide dismutase (SODC) | Response to oxidative stress | [38] |
| 62 | WP_038238706.1 | Periplasmic | Copper/zinc superoxide dismutase (SODC) | Response to oxidative stress | [38] |
| 63 | WP_053008603.1 | Periplasmic | Cupredoxin/Blue (type 1) copper domain | Transport | [39] |
| 64 | WP_003470615.1 | Cytoplasmic | Cyclophilin type peptidyl-prolyl cis-trans isomerase/CLD | Protein folding | [40,41] |
| 65 | WP_003465861.1 | Cytoplasmic | Cytidine and deoxycytidylate deaminase zinc-binding region | Metabolic process | [42] |
| 66 | WP_003466552.1 | Cytoplasmic | Cytidine and deoxycytidylate deaminase zinc-binding region | Metabolic process | [42] |
| 67 | WP_003472695.1 | Cytoplasmic | Cytidine and deoxycytidylate deaminase zinc-binding region | Metabolic process | [42] |
| 68 | WP_047324934.1 | Periplasmic | Cytochrome c | Transport | [43] |
| 69 | WP_047325269.1 | Periplasmic | Cytochrome c | Transport | [43] |
| 70 | WP_003471850.1 | InnerMembrane | Cytochrome C and Quinol oxidase polypeptide I | Transport | [44,45] |
| 71 | WP_047324660.1 | InnerMembrane | Cytochrome C and Quinol oxidase polypeptide I | Transport | [44,45] |
| 72 | WP_047324475.1 | InnerMembrane | Cytochrome C oxidase subunit II, periplasmic domain | Transport | [44,45] |
| 73 | WP_003469645.1 | InnerMembrane | Cytochrome c oxidase subunit III | Transport | [46] |
| 74 | WP_003471844.1 | InnerMembrane | Cytochrome c oxidase subunit III | Transport | [46] |
| 75 | WP_047325029.1 | InnerMembrane | Cytochrome C1 | Transport | [44,45] |
| 76 | WP_038237017.1 | Cytoplasmic | Cytochrome P450 | Metabolic process | [47] |
| 77 | WP_047325129.1 | Cytoplasmic | Cytochrome P450 | Metabolic process | [47] |
| 78 | WP_047325324.1 | Cytoplasmic | DEAD/DEAH box helicase | Metabolic process | [48] |
| 79 | WP_047324691.1 | Cytoplasmic | DEAD/H associated | Metabolic process | [48] |
| 80 | WP_003471132.1 | Cytoplasmic | Delta-aminolevulinic acid dehydratase | Metabolic process | [49] |
| 81 | WP_047324738.1 | Cytoplasmic | Di-haem cytochrome c peroxidase | Response to oxidative stress | [50,51] |
| 82 | WP_003465985.1 | Periplasmic | Dipeptidyl peptidase IV (DPP IV) N-terminal region | Proteolysis | [52,53] |
| 83 | WP_047324427.1 | Periplasmic | Dipeptidyl peptidase IV (DPP IV) N-terminal region | Proteolysis | [52,53] |
| 84 | WP_047325424.1 | Periplasmic | Dipeptidyl peptidase IV (DPP IV) N-terminal region | Proteolysis | [52,53] |
| 85 | WP_003469990.1 | Cytoplasmic | DNA gyrase B | DNA replication | [54] |
| 86 | WP_003471299.1 | Cytoplasmic | DNA gyrase B | DNA replication | [54] |
| 87 | WP_003467030.1 | Cytoplasmic | DNA polymerase family A | DNA replication | [55] |
| 88 | WP_003470923.1 | Cytoplasmic | DNA polymerase III, delta subunit | DNA replication | [56] |
| 89 | WP_003470629.1 | Cytoplasmic | DNA-directed RNA polymerase, alpha subunit | Transcription regulation | [57] |
| 90 | WP_047325270.1 | Periplasmic | DSBA-like thioredoxin domain | Protein folding | [58] |
| 91 | WP_047325670.1 | Periplasmic | DSBA-like thioredoxin domain | Protein folding | [58] |
| 92 | WP_003467872.1 | Cytoplasmic | D-Tyr-tRNA(Tyr) deacylase | RNA processing | [59] |
| 93 | WP_003470942.1 | Cytoplasmic | EAL domain | Cell signaling | [60] |
| 94 | WP_047324687.1 | Cytoplasmic | Elongation factor G, III-V domain | Protein biosynthesis | [61] |
| 95 | WP_003465522.1 | Cytoplasmic | Enoyl-CoA hydratase/isomerase | Metabolic process | [62] |
| 96 | WP_003468480.1 | Cytoplasmic | Enoyl-CoA hydratase/isomerase | Metabolic process | [62] |
| 97 | WP_003472015.1 | Cytoplasmic | Enoyl-CoA hydratase/isomerase | Metabolic process | [62] |
| 98 | WP_047325628.1 | Cytoplasmic | Enoyl-CoA hydratase/isomerase | Metabolic process | [62] |
| 99 | WP_003466549.1 | Cytoplasmic | Exonuclease, RNase T | RNA processing | [63] |
| 100 | WP_003465697.1 | Cytoplasmic | Extradiol aromatic ring-opening dioxygenase, DODA-type | Metabolic process | [64] |
| 101 | WP_003469000.1 | InnerMembrane | FAD binding domain | Metabolic process | [65] |
| 102 | WP_003467994.1 | Cytoplasmic | Ferric uptake regulator family | Transcription regulation | [66] |
| 103 | WP_003468858.1 | Cytoplasmic | Ferritin/DPS protein domain | Response to oxidative stress | [67] |
| 104 | WP_047324648.1 | Cytoplasmic | Ferritin/DPS protein domain | Response to oxidative stress | [67] |
| 105 | WP_004426268.1 | Cytoplasmic | FGGY family of carbohydrate kinases, N-terminal domain | Metabolic process | [68] |
| 106 | WP_003465369.1 | Cytoplasmic | Fibronectin type III-like domain | Metabolic process | [69] |
| 107 | WP_004426584.1 | Periplasmic | FKBP-type peptidyl-prolyl cis-trans isomerase domain | Protein folding | [70–72] |
| 108 | WP_038239202.1 | Periplasmic | FKBP-type peptidyl-prolyl cis-trans isomerase domain | Protein folding | [70–72] |
| 109 | WP_003470030.1 | Cytoplasmic | Fructose-1-6-bisphosphatase, N-terminal domain | Metabolic process | [73] |
| 110 | WP_003471967.1 | Cytoplasmic | GAT_1 superfamily | Metabolic process | [74] |
| 111 | WP_003466789.1 | Cytoplasmic | GDP-mannose 4,6 dehydratase | Metabolic process | [75] |
| 112 | WP_003471228.1 | Cytoplasmic | GGDEF domain | Cell Signaling | [76] |
| 113 | WP_047324417.1 | Cytoplasmic | GGDEF domain | Cell Signaling | [76] |
| 114 | WP_047324698.1 | Cytoplasmic | Glu/Leu/Phe/Val dehydrogenase, dimerisation domain | Metabolic process | [77] |
| 115 | WP_047325669.1 | Cytoplasmic | Glutathione-dependent formaldehyde-activating enzyme | Metabolic process | [78] |
| 116 | WP_003469507.1 | Cytoplasmic | Glyceraldehyde 3-phosphate dehydrogenase | Metabolic process | [79] |
| 117 | WP_003469871.1 | Periplasmic | Glycoside hydrolase family 10 | Metabolic process | [80–82] |
| 118 | WP_003469868.1 | Periplasmic | Glycoside hydrolase family 10 | Metabolic process | [80–82] |
| 119 | WP_047325488.1 | Periplasmic | Glycoside hydrolase family 3 | Metabolic process | [80–82] |
| 120 | WP_080964854.1 | Periplasmic | Glycoside hydrolase family 3 | Metabolic process | [80–82] |
| 121 | WP_047325137.1 | Periplasmic | Glycosyl hydrolase family 20, catalytic domain | Metabolic process | [80–82] |
| 122 | WP_003467345.1 | Cytoplasmic | Glyoxalase/Bleomycin resistance protein/Dioxygenase superfamily | Metabolic process | [85] |
| 123 | WP_047325053.1 | Cytoplasmic | HhH-GPD superfamily base excision DNA repair protein | DNA repair | [86] |
| 124 | WP_003469598.1 | Cytoplasmic | Histidinol dehydrogenase | Metabolic process | [87] |
| 125 | WP_047324567.1 | Cytoplasmic | Histone deacetylase domain | Transcription regulation | [88] |
| 126 | WP_003468608.1 | Cytoplasmic | HIT domain | Metabolic process | [89] |
| 127 | WP_047324715.1 | Cytoplasmic | Homocysteine S-methyltransferase | Metabolic process | [90] |
| 128 | WP_047324842.1 | Cytoplasmic | HSP40/DnaJ peptide-binding | Protein folding | [72,91] |
| 129 | WP_003468012.1 | Cytoplasmic | Hsp70 protein/DnaK | Protein folding | [72,91] |
| 130 | WP_004426468.1 | Cytoplasmic | Hsp70 protein/DnaK | Protein folding | [72,91] |
| 131 | WP_003468546.1 | Cytoplasmic | Hydroxyacylglutathione hydrolase C-terminus | Metabolic process | [92] |
| 132 | WP_047324805.1 | Cytoplasmic | Imidazoleglycerol-phosphate dehydratase | Metabolic process | [93] |
| 133 | WP_003466074.1 | Cytoplasmic | Inositol monophosphatase family | Cell signaling | [94] |
| 134 | WP_003472753.1 | Cytoplasmic | Inositol monophosphatase family | Cell signaling | [94] |
| 135 | WP_003477578.1 | Cytoplasmic | Integrase core domain | DNA integration | [95] |
| 136 | WP_047324635.1 | Cytoplasmic | Integrase core domain | DNA integration | [95] |
| 137 | WP_003466555.1 | Cytoplasmic | Iron dependent repressor, metal binding and dimerisation domain | Response to oxidative stress | [96] |
| 138 | WP_003468529.1 | Cytoplasmic | Isocitrate/isopropylmalate dehydrogenase | Metabolic process | [97] |
| 139 | WP_047325244.1 | Cytoplasmic | Isocitrate/isopropylmalate dehydrogenase | Metabolic process | [97] |
| 140 | WP_003466920.1 | Periplasmic | LacI-type HTH domain | Transcription regulation | [98] |
| 141 | WP_047324718.1 | Cytoplasmic | Leucyl-tRNA synthetase, Domain 2 | RNA processing | [99] |
| 142 | WP_047325709.1 | Cytoplasmic | Maltose/galactoside acetyltransferase | Response to oxidative stress | [100] |
| 143 | WP_004425502.1 | InnerMembrane | Membrane dipeptidase (Peptidase family M19) | Proteolysis | [101] |
| 144 | WP_003473181.1 | Cytoplasmic | MerR HTH family regulatory protein | Transcription regulation | [102] |
| 145 | WP_003470478.1 | Cytoplasmic | Metal-dependent hydrolase | Metabolic process | [103] |
| 146 | WP_047324776.1 | Cytoplasmic | Metal-dependent hydrolase | Metabolic process | [103] |
| 147 | WP_047324781.1 | Cytoplasmic | Metal-dependent hydrolase | Metabolic process | [103] |
| 148 | WP_047325273.1 | Cytoplasmic | Metal-dependent hydrolase | Metabolic process | [103] |
| 149 | WP_047325218.1 | Cytoplasmic | Metallo-beta-lactamase | Antimicrobial resistance | [104] |
| 150 | WP_038236501.1 | Periplasmic | Metalloenzyme, LuxS/M16 peptidase-like | Proteolysis | [105] |
| 151 | WP_047324411.1 | Periplasmic | Metalloenzyme, LuxS/M16 peptidase-like | Proteolysis | [105] |
| 152 | WP_047324767.1 | Cytoplasmic | Metallopeptidase family M24 | Proteolysis | [101] |
| 153 | WP_047325232.1 | Cytoplasmic | Metallopeptidase family M24 | Proteolysis | [101] |
| 154 | WP_080627636.1 | Cytoplasmic | Metallopeptidase family M24 | Proteolysis | [101] |
| 155 | WP_003471664.1 | Cytoplasmic | Methyladenine glycosylase | DNA repair | [106] |
| 156 | WP_003469968.1 | Cytoplasmic | MnmE helical domain | RNA processing | [107] |
| 157 | WP_003465837.1 | Cytoplasmic | MreB/Mbl protein | Cell signaling | [108] |
| 158 | WP_003472119.1 | Cytoplasmic | MutL C terminal dimerisation domain | DNA repair | [109] |
| 159 | WP_047325525.1 | Periplasmic | N-acetylmuramoyl-L-alanine amidase | Metabolic process | [110] |
| 160 | WP_047324950.1 | Cytoplasmic | NAD dependent epimerase/dehydratase family | Metabolic process | [111] |
| 161 | WP_047325529.1 | Cytoplasmic | NAD dependent epimerase/dehydratase family | Metabolic process | [111] |
| 162 | WP_003465966.1 | Cytoplasmic | NUDIX domain | Metabolic process | [112] |
| 163 | WP_003472750.1 | Cytoplasmic | NUDIX domain | Metabolic process | [112] |
| 164 | WP_003481247.1 | Cytoplasmic | NUDIX domain | Metabolic process | [112] |
| 165 | WP_004425664.1 | Cytoplasmic | NUDIX domain | Metabolic process | [112] |
| 166 | WP_038238963.1 | Cytoplasmic | NUDIX domain | Metabolic process | [112] |
| 167 | WP_003468516.1 | Cytoplasmic | OmpR/PhoB-type DNA-binding domain | Transcription regulation | [113] |
| 168 | WP_047324797.1 | Cytoplasmic | Pantoate ligase | Metabolic process |  |
| 169 | WP_003465625.1 | OuterMembrane | Penicillin-binding protein 1B | Antimicrobial resistance | [114,115] |
| 170 | WP_047324608.1 | InnerMembrane | Penicillin-binding protein 1C | Antimicrobial resistance | [114,115] |
| 171 | WP_047324537.1 | Periplasmic | Peptidase family M1 domain | Proteolysis | [116] |
| 172 | WP_047324609.1 | Periplasmic | Peptidase family M1 domain | Proteolysis | [116] |
| 173 | WP_047324744.1 | Periplasmic | Peptidase family M13 | Proteolysis | [117] |
| 174 | WP_047324745.1 | Periplasmic | Peptidase family M13 | Proteolysis | [117] |
| 175 | WP_047325067.1 | Cytoplasmic | Peptidase family M20/M25/M40 | Metabolic process | [118,119] |
| 176 | WP_003471878.1 | Periplasmic | Peptidase family M28 | Proteolysis | [120] |
| 177 | WP_047325199.1 | Periplasmic | Peptidase family M28 | Proteolysis | [120] |
| 178 | WP_047325436.1 | Periplasmic | Peptidase family M28 | Proteolysis | [120] |
| 179 | WP_003473187.1 | Periplasmic | Peptidase family M3 | Proteolysis | [101] |
| 180 | WP_004425920.1 | Periplasmic | Peptidase family M3 | Proteolysis | [101] |
| 181 | WP_003471337.1 | InnerMembrane | Peptidase family M41 | Proteolysis | [121] |
| 182 | WP_080964790.1 | OuterMembrane | Peptidase M23 | Antimicrobial resistance | [122] |
| 183 | WP_003466981.1 | Cytoplasmic | Peptide chain release factor 3 | Protein biosynthesis | [123] |
| 184 | WP_003471408.1 | Cytoplasmic | Peptidyl-prolyl cis-trans isomerase domain | Protein folding | [70] |
| 185 | WP_003472896.1 | Periplasmic | Peptidyl-prolyl cis-trans isomerase domain | Protein folding | [70] |
| 186 | WP_047324673.1 | Periplasmic | Periplasmic-binding protein YtfQ | Transport | [124] |
| 187 | WP_003466655.1 | Cytoplasmic | Phenylalanyl-tRNA synthetase | Protein biosynthesis | [125] |
| 188 | WP_003466794.1 | Cytoplasmic | Phosphoglucomutase/phosphomannomutase, alpha/beta/alpha domain I | Metabolic process | [126] |
| 189 | WP_047324462.1 | Cytoplasmic | Phosphoglucomutase/phosphomannomutase, alpha/beta/alpha domain I | Metabolic process | [126] |
| 190 | WP_047324753.1 | Cytoplasmic | Phosphoglucomutase/phosphomannomutase, alpha/beta/alpha domain I | Metabolic process | [126] |
| 191 | WP_003468806.1 | Cytoplasmic | Phosphopantetheine attachment site | Metabolic process | [127] |
| 192 | WP_003466735.1 | Cytoplasmic | Pilus retraction protein PilT | Transport | [128] |
| 193 | WP_003471666.1 | Cytoplasmic | Pilus retraction protein PilT | Transport | [128] |
| 194 | WP_003472490.1 | Cytoplasmic | Polypeptide deformylase | Protein biosynthesis | [129] |
| 195 | WP_047324501.1 | Cytoplasmic | Polypeptide deformylase | Protein biosynthesis | [129] |
| 196 | WP_047325261.1 | InnerMembrane | Polysaccharide deacetylase | Metabolic process | [130] |
| 197 | WP_047324952.1 | Cytoplasmic | Pre-ATP-grasp domain | Metabolic process | [131] |
| 198 | WP_047325103.1 | Periplasmic | Prolyl oligopeptidase family | Proteolysis | [132] |
| 199 | WP_047325465.1 | Periplasmic | Prolyl oligopeptidase family | Proteolysis | [132] |
| 200 | WP_047325264.1 | Periplasmic | Putative esterase | Proteolysis | [133] |
| 201 | WP_003472675.1 | Cytoplasmic | Pyridine nucleotide-disulphide oxidoreductase | Response to oxidative stress | [134,135] |
| 202 | WP_003468017.1 | Cytoplasmic | Pyridoxamine kinase/Phosphomethylpyrimidine kinase | Metabolic process | [136] |
| 203 | WP_003471593.1 | Cytoplasmic | Pyridoxamine kinase/Phosphomethylpyrimidine kinase | Metabolic process | [136] |
| 204 | WP_038236644.1 | Cytoplasmic | Queuine tRNA-ribosyltransferase | RNA processing | [137] |
| 205 | WP_003465234.1 | Cytoplasmic | Response regulator receiver domain | Cell signaling | [138,139] |
| 206 | WP_003465620.1 | Cytoplasmic | Response regulator receiver domain | Cell signaling | [138,139] |
| 207 | WP_003466157.1 | Cytoplasmic | Response regulator receiver domain | Cell signaling | [138,139] |
| 208 | WP_003467543.1 | Cytoplasmic | Response regulator receiver domain | Cell signaling | [138,139] |
| 209 | WP_003469200.1 | Cytoplasmic | Response regulator receiver domain | Cell signaling | [138,139] |
| 210 | WP_003469280.1 | Cytoplasmic | Response regulator receiver domain | Cell signaling | [138,139] |
| 211 | WP_003470782.1 | Cytoplasmic | Response regulator receiver domain | Cell signaling | [138,139] |
| 212 | WP_003472647.1 | Cytoplasmic | Response regulator receiver domain | Cell signaling | [138,139] |
| 213 | WP_003472648.1 | Cytoplasmic | Response regulator receiver domain | Cell signaling | [138,139] |
| 214 | WP_003481607.1 | Cytoplasmic | Response regulator receiver domain | Cell signaling | [138,139] |
| 215 | WP_047324961.1 | Cytoplasmic | Response regulator receiver domain | Cell signaling | [138,139] |
| 216 | WP_047325048.1 | Cytoplasmic | Response regulator receiver domain | Cell signaling | [138,139] |
| 217 | WP_003473476.1 | Cytoplasmic | Ribonuclease D | RNA processing | [140] |
| 218 | WP_003476601.1 | Cytoplasmic | Ribonucleotide reductase, small chain | Metabolic process | [141] |
| 219 | WP_003470669.1 | Cytoplasmic | Ribosomal protein L2, bacterial/organellar-type | Protein biosynthesis | [142] |
| 220 | WP_003469157.1 | Cytoplasmic | Ribosomal protein S12/S23 | Protein biosynthesis | [143] |
| 221 | WP_003466792.1 | Cytoplasmic | RmlD substrate binding domain | Metabolic process | [144] |
| 222 | WP_003467386.1 | Cytoplasmic | RNase_PH | RNA processing | [145,146] |
| 223 | WP_080964784.1 | Cytoplasmic | RNB domain | RNA processing | [145,146] |
| 224 | WP_003477416.1 | Cytoplasmic | Rubredoxin | Transport | [147] |
| 225 | WP_047325217.1 | Periplasmic | S1/P1 Nuclease | Metabolic process | [148] |
| 226 | WP_047324777.1 | Cytoplasmic | S-adenosyl-L-methionine-dependent methyltransferase | Metabolic process | [149] |
| 227 | WP_003465502.1 | Cytoplasmic | SecA DEAD-like domain | Transport | [150] |
| 228 | WP_003471965.1 | Cytoplasmic | SET domain | Transcription regulation | [151] |
| 229 | WP_047324433.1 | Cytoplasmic | Shikimate dehydrogenase substrate binding domain | Metabolic process | [152] |
| 230 | WP_003465242.1 | Cytoplasmic | Short-chain dehydrogenase/reductase SDR | Metabolic process | [153,154] |
| 231 | WP_003465445.1 | Cytoplasmic | Short-chain dehydrogenase/reductase SDR | Metabolic process | [153,154] |
| 232 | WP_003466959.1 | Cytoplasmic | Short-chain dehydrogenase/reductase SDR | Metabolic process | [153,154] |
| 233 | WP_003467341.1 | Cytoplasmic | Short-chain dehydrogenase/reductase SDR | Metabolic process | [153,154] |
| 234 | WP_003468235.1 | Cytoplasmic | Short-chain dehydrogenase/reductase SDR | Metabolic process | [153,154] |
| 235 | WP_003468804.1 | Cytoplasmic | Short-chain dehydrogenase/reductase SDR | Metabolic process | [153,154] |
| 236 | WP_003469170.1 | Cytoplasmic | Short-chain dehydrogenase/reductase SDR | Metabolic process | [153,154] |
| 237 | WP_003472018.1 | Cytoplasmic | Short-chain dehydrogenase/reductase SDR | Metabolic process | [153,154] |
| 238 | WP_003472098.1 | Cytoplasmic | Short-chain dehydrogenase/reductase SDR | Metabolic process | [153,154] |
| 239 | WP_004425521.1 | Periplasmic | Short-chain dehydrogenase/reductase SDR | Metabolic process | [153,154] |
| 240 | WP_047324452.1 | Cytoplasmic | Short-chain dehydrogenase/reductase SDR | Metabolic process | [153,154] |
| 241 | WP_047324671.1 | Cytoplasmic | Short-chain dehydrogenase/reductase SDR | Metabolic process | [153,154] |
| 242 | WP_047324755.1 | Cytoplasmic | Short-chain dehydrogenase/reductase SDR | Metabolic process | [153,154] |
| 243 | WP_047324951.1 | Cytoplasmic | Short-chain dehydrogenase/reductase SDR | Metabolic process | [153,154] |
| 244 | WP_047325420.1 | Cytoplasmic | Short-chain dehydrogenase/reductase SDR | Metabolic process | [153,154] |
| 245 | WP_047325583.1 | Cytoplasmic | Short-chain dehydrogenase/reductase SDR | Metabolic process | [153,154] |
| 246 | WP_004425727.1 | Cytoplasmic | Sialic acid O-acyltransferase NeuD-like | Metabolic process | [155] |
| 247 | WP_047324520.1 | Cytoplasmic | Sigma-54 interaction domain | Transcription regulation | [156] |
| 248 | WP_003465967.1 | InnerMembrane | SRP54-type protein, GTPase domain | Cell signaling | [157] |
| 249 | WP_047324677.1 | InnerMembrane | SRP54-type protein, GTPase domain | Cell signaling | [157] |
| 250 | WP_003466372.1 | Cytoplasmic | Succinyl-CoA synthetase-like | Metabolic process | [158] |
| 251 | WP_003470294.1 | InnerMembrane | Sulphite reductase [NADPH] flavoprotein, alpha chain | Metabolic process | [159] |
| 252 | WP_003467943.1 | Cytoplasmic | TatD related DNase | DNA repair | [160] |
| 253 | WP_003467941.1 | Cytoplasmic | ThiF family | Metabolic process | [161] |
| 254 | WP_003468623.1 | Cytoplasmic | ThiF family | Metabolic process | [161] |
| 255 | WP_003470617.1 | Cytoplasmic | Translation elongation factor EFG, V domain | Protein biosynthesis | [162] |
| 256 | WP_003469161.1 | Cytoplasmic | Translation elongation factor EFG/EF2, domain IV | Protein biosynthesis | [162] |
| 257 | WP_047324579.1 | Cytoplasmic | Translation elongation factor EFTu/EF1A, bacterial/organelle | Protein biosynthesis | [162] |
| 258 | WP_004425677.1 | Cytoplasmic | Translation initiation factor 1A / IF-1 | Protein biosynthesis | [163] |
| 259 | WP_003473108.1 | Cytoplasmic | Trimeric LpxA-like | Metabolic process | [164] |
| 260 | WP_047324810.1 | Cytoplasmic | tRNA synthetase class II core domain (G H P S and T) | Protein biosynthesis | [165,166] |
| 261 | WP_003470124.1 | Cytoplasmic | tRNA synthetases class I (C) catalytic domain | Protein biosynthesis | [167] |
| 262 | WP_003472685.1 | Cytoplasmic | tRNA synthetases class I (E and Q) catalytic domain | Protein biosynthesis | [167] |
| 263 | WP_003472954.1 | Cytoplasmic | tRNA synthetases class I (E and Q) catalytic domain | Protein biosynthesis | [167] |
| 264 | WP_003469626.1 | Cytoplasmic | tRNA synthetases class I (I L M and V) | Protein biosynthesis | [167] |
| 265 | WP_047324986.1 | Cytoplasmic | tRNA synthetases class II (A) | Protein biosynthesis | [165,166] |
| 266 | WP_003472142.1 | Cytoplasmic | tRNA synthetases class II (D K and N) | Protein biosynthesis | [165,166] |
| 267 | WP_003472589.1 | Cytoplasmic | tRNA synthetases class II (D K and N) | Protein biosynthesis | [165,166] |
| 268 | WP_047324979.1 | Cytoplasmic | tRNA synthetases class II (D K and N) | Protein biosynthesis | [165,166] |
| 269 | WP_047324485.1 | Cytoplasmic | Type III restriction enzyme, res subunit | DNA repair | [168] |
| 270 | WP_003468255.1 | Cytoplasmic | Ultra-violet resistance protein B | DNA repair | [169] |
| 271 | WP_047325123.1 | Cytoplasmic | Uroporphyrinogen decarboxylase (URO-D) | Metabolic process | [170] |
| 272 | WP_047324617.1 | Cytoplasmic | UvrA | DNA repair | [171] |
| 273 | WP_003468914.1 | Cytoplasmic | UvrABC system subunit A | DNA repair | [171] |
| 274 | WP_003470165.1 | Cytoplasmic | Xylose isomerase-like TIM barrel | DNA repair | [172] |
| 275 | WP_047325492.1 | Cytoplasmic | Xylose isomerase-like TIM barrel | DNA repair | [172] |
| 276 | WP_003465432.1 | Cytoplasmic | YbbN/Trx-like domain | Protein folding | [173] |
| 277 | WP_003469256.1 | Cytoplasmic | YbbN/Trx-like domain | Protein folding | [173] |
| 278 | WP_047324717.1 | Cytoplasmic | YbbN/Trx-like domain | Protein folding | [173] |
| 279 | WP_003468538.1 | Cytoplasmic | Zinc finger, ClpX C4-type | Transcription regulation | [174] |

**List of supplementary references**

1. Eoh H, Brennan PJ, Crick DC. 2009 The Mycobacterium tuberculosis MEP (2C-methyl-d-erythritol 4-phosphate) pathway as a new drug target. *Tuberculosis*. (doi:10.1016/j.tube.2008.07.004)

2. Koon N, Squire CJ, Baker EN. 2004 Crystal structure of LeuA from Mycobacterium tuberculosis, a key enzyme in leucine biosynthesis. *Proceedings of the National Academy of Sciences* (doi:10.1073/pnas.0400820101)

3. Kumar P, Singh M, Gautam R, Karthikeyan S. 2010 Potential anti-bacterial drug target: Structural characterization of 3,4-dihydroxy-2-butanone-4-phosphate synthase from Salmonella typhimurium LT2. *Proteins: Structure, Function and Bioinformatics* (doi:10.1002/prot.22837)

4. Yang X, Dubnau E, Smith I, Sampson NS. 2007 Rv1106c from Mycobacterium tuberculosis is a 3β-hydroxysteroid dehydrogenase. *Biochemistry* **46**, 9058–9067.

5. Chandran SS, Frost JW. 2001 Aromatic inhibitors of dehydroquinate synthase: Synthesis, evaluation and implications for gallic acid biosynthesis. *Bioorganic and Medicinal Chemistry Letters* (doi:10.1016/S0960-894X(01)00065-8)

6. Kang Y, Zarzycki-Siek J, Walton CB, Norris MH, Hoang TT. 2010 Multiple fadd acyl-CoA synthetases contribute to differential fatty acid degradation and virulence in pseudomonas aeruginosa. *PLoS ONE* (doi:10.1371/journal.pone.0013557)

7. Blombach F, Launay H, Zorraquino V, Swarts DC, Cabrita LD, Benelli D, Christodoulou J, Londei P, van der Oost J. 2011 An HflX-Type GTPase from sulfolobus solfataricus binds to the 50S ribosomal subunit in all nucleotide-bound states. *Journal of Bacteriology* (doi:10.1128/JB.01552-10)

8. Allen WJ, Li Y, Waksman G. 2001 Bacterial DNA Polymerase I. *e LS*

9. Okuno T, Yamanaka K, Ogura T. 2006 An AAA protease FtsH can initiate proteolysis from internal sites of a model substrate, apo-flavodoxin. *Genes to Cells* (doi:10.1111/j.1365-2443.2006.00940.x)

10. Capdevila DA, Wang J, Giedroc DP. 2016 Bacterial strategies to maintain zinc metallostasis at the host-pathogen interface. *Journal of Biological Chemistry*. **291**, 20858–20868. (doi:10.1074/jbc.R116.742023)

11. Tanaka KJ, Song S, Mason K, Pinkett HW. 2017 Selective substrate uptake: The role of ATP-binding cassette (ABC) importers in pathogenesis. *Biochimica et Biophysica Acta - Biomembranes*. (doi:10.1016/j.bbamem.2017.08.011)

12. Davis MS, Cronan JE. 2001 Inhibition of Escherichia coliAcetyl Coenzyme A Carboxylase by Acyl-Acyl Carrier Protein. *Journal of bacteriology* **183**, 1499–1503.

13. Thach TT, Luong TT, Lee S, Rhee DK. 2014 Adenylate kinase from Streptococcus pneumoniae is essential for growth through its catalytic activity. *FEBS Open Bio* (doi:10.1016/j.fob.2014.07.002)

14. Hansen TA. 1994 Metabolism of sulfate-reducing prokaryotes. *Antonie van Leeuwenhoek* (doi:10.1007/BF00871638)

15. Karlsson C, Jörnvall H, Höög J-O. 1995 Zinc binding of alcohol and sorbitol dehydrogenases. In *Enzymology and Molecular Biology of Carbonyl Metabolism 5*, pp. 397–406. Springer.

16. Beckham KSH *et al.* 2014 The metabolic enzyme AdhE controls the virulence of Escherichia coliO157: H7. *Molecular Microbiology* **93**, 199–211. (doi:10.1111/mmi.12651)

17. Luong TT, Kim EH, Bak JP, Nguyen CT, Choi S, Briles DE, Pyo S, Rhee DK. 2015 Ethanol-induced alcohol dehydrogenase E (AdhE) potentiates pneumolysin in Streptococcus pneumoniae. *Infection and Immunity* **83**, 108–119. (doi:10.1128/IAI.02434-14)

18. Barski OA, Tipparaju SM, Bhatnagar A. 2008 The aldo-keto reductase superfamily and its role in drug metabolism and detoxification. *Drug metabolism reviews* **40**, 553–624.

19. Sultana R, Tanneeru K, Guruprasad L. 2011 The PE-PPE domain in mycobacterium reveals a serine α/β hydrolase fold and function: an in-silico analysis. *PloS one* **6**, e16745.

20. Oikawa T. 2006 Alanine, aspartate, and asparagine metabolism in microorganisms. In *Amino Acid Biosynthesis~ Pathways, Regulation and Metabolic Engineering*, pp. 273–288. Springer.

21. Marino F, Mojumdar A, Zucchelli C, Bhardwaj A, Buratti E, Vindigni A, Musco G, Onesti S. 2016 Structural and biochemical characterization of an RNA/DNA binding motif in the N-terminal domain of RecQ4 helicases. *Scientific reports* **6**, 21501.

22. Tseng Y-T, Chiou N-T, Gogiraju R, Lin-Chao S. 2015 The protein interaction of RNA helicase B (RhlB) and polynucleotide phosphorylase (PNPase) contributes to the homeostatic control of cysteine in Escherichia coli. *Journal of Biological Chemistry* **290**, 29953–29963.

23. Galperin MY, Koonin E V. 1997 A diverse superfamily of enzymes with ATP‐dependent carboxylate—amine/thiol ligase activity. *Protein Science* **6**, 2639–2643.

24. Neupane DP, Avalos D, Fullam S, Roychowdhury H, Yukl ET. 2017 Mechanisms of zinc binding to the solute-binding protein AztC and transfer from the metallochaperone AztD. *Journal of Biological Chemistry* **292**, 17496–17505.

25. Vuorio R, Härkönen T, Tolvanen M, Vaara M. 1994 The novel hexapeptide motif found in the acyltransferases LpxA and LpxD of lipid A biosynthesis is conserved in various bacteria. *FEBS letters* **337**, 289–292.

26. Janowski R, Auerbach‐Nevo T, Weiss MS. 2008 Bacterioferritin from Mycobacterium smegmatis contains zinc in its di‐nuclear site. *Protein Science* **17**, 1138–1150.

27. Wang G, Olczak AA, Walton JP, Maier RJ. 2005 Contribution of the Helicobacter pylori thiol peroxidase bacterioferritin comigratory protein to oxidative stress resistance and host colonization. *Infection and immunity* **73**, 378–384.

28. Tamilselvi A, Mugesh G. 2008 Zinc and antibiotic resistance: metallo-β-lactamases and their synthetic analogues. *JBIC Journal of Biological Inorganic Chemistry* **13**, 1039–1053.

29. Capasso C, T Supuran C. 2017 Inhibition of bacterial carbonic anhydrases as a novel approach to escape drug resistance. *Current topics in medicinal chemistry* **17**, 1237–1248.

30. Bilder P *et al.* 2006 The structure of the carboxyltransferase component of acetyl-coA carboxylase reveals a zinc-binding motif unique to the bacterial enzyme. *Biochemistry* **45**, 1712–1722.

31. Biundo A, Steinkellner G, Gruber K, Spreitzhofer T, Ribitsch D, Guebitz GM. 2017 Engineering of the zinc-binding domain of an esterase from Clostridium botulinum towards increased activity on polyesters. *Catalysis Science and Technology* (doi:10.1039/c7cy00168a)

32. Lun S, Bishai WR. 2007 Characterization of a novel cell wall-anchored protein with carboxylesterase activity required for virulence in Mycobacterium tuberculosis. *Journal of Biological Chemistry* (doi:10.1074/jbc.M700035200)

33. Tomme P, Driver DP, Amandoron EA, Miller RC, Antony R, Warren J, Kilburn DG. 1995 Comparison of a fungal (family I) and bacterial (family II) cellulose-binding domain. *Journal of bacteriology* **177**, 4356–4363.

34. Frees D, Gerth U, Ingmer H. 2014 Clp chaperones and proteases are central in stress survival, virulence and antibiotic resistance of Staphylococcus aureus. *International Journal of Medical Microbiology*. **304**, 142–149. (doi:10.1016/j.ijmm.2013.11.009)

35. Huang L, Qin Y, Yan Q, Lin G, Huang L, Huang B, Huang W. 2015 MinD plays an important role in Aeromonas hydrophila adherence to Anguilla japonica mucus. *Gene* **565**, 275–281.

36. Kim JS, Park SJ, Kwak KJ, Kim YO, Kim JY, Song J, Jang B, Jung C-H, Kang H. 2006 Cold shock domain proteins and glycine-rich RNA-binding proteins from Arabidopsis thaliana can promote the cold adaptation process in Escherichia coli. *Nucleic acids research* **35**, 506–516.

37. Lu M, Li Z, Liang J, Wei Y, Rensing C, Wei G. 2016 Zinc Resistance Mechanisms of P 1B-type ATPases in Sinorhizobium meliloti CCNWSX0020. *Scientific Reports* **6**. (doi:10.1038/srep29355)

38. Fones H, Preston GM. 2012 Reactive oxygen and oxidative stress tolerance in plant pathogenic Pseudomonas. *FEMS Microbiology Letters*. **327**, 1–8. (doi:10.1111/j.1574-6968.2011.02449.x)

39. Adman ET. 1991 Copper protein structures. In *Advances in protein chemistry*, pp. 145–197. Elsevier.

40. Krummrei U, Bang R, Schmidtchen R, Brune K, Bang H. 1995 Cyclophilin‐A is a zinc‐dependent DNA binding protein in macrophages. *FEBS letters* **371**, 47–51.

41. Roset MS, Fernández LG, DelVecchio VG, Briones G. 2013 Intracellularly induced cyclophilins play an important role in stress adaptation and virulence of Brucella abortus. *Infection and immunity* **81**, 521–530.

42. Li Y, Guo Z, Jin L, Wang D, Gao Z, Su X, Hou H, Dong Y. 2016 Mechanism of the allosteric regulation of Streptococcus mutans 2′-deoxycytidylate deaminase. *Acta Crystallographica Section D: Structural Biology* **72**, 883–891.

43. Long AR, Anthony C. 1991 Characterization of the periplasmic cytochromes c of Paracoccus denitrificans: identification of the electron acceptor for methanol dehydrogenase, and description of a novel cytochrome c heterodimer. *Microbiology* **137**, 415–425.

44. Lee D-W, El Khoury Y, Francia F, Zambelli B, Ciurli S, Venturoli G, Hellwig P, Daldal F. 2011 Zinc inhibition of bacterial cytochrome bc 1 reveals the role of cytochrome b E295 in proton release at the Qo site. *Biochemistry* **50**, 4263–4272.

45. Mills DA, Schmidt B, Hiser C, Westley E, Ferguson-Miller S. 2002 Membrane Potential-controlled Inhibition of Cytochromec Oxidase by Zinc. *Journal of Biological Chemistry* **277**, 14894–14901.

46. Ekici S, Pawlik G, Lohmeyer E, Koch HG, Daldal F. 2012 Biogenesis of cbb3-type cytochrome c oxidase in Rhodobacter capsulatus. *Biochimica et Biophysica Acta - Bioenergetics*. **1817**, 898–910. (doi:10.1016/j.bbabio.2011.10.011)

47. Miller CD, Pettee B, Zhang C, Pabst M, McLean JE, Anderson AJ. 2009 Copper and cadmium: Responses in Pseudomonas putida KT2440. *Letters in Applied Microbiology* (doi:10.1111/j.1472-765X.2009.02741.x)

48. Linder P. 2006 Dead-box proteins: a family affair—active and passive players in RNP-remodeling. *Nucleic acids research* **34**, 4168–4180.

49. Chauhan S, O’Brian MR. 1995 A mutant Bradyrhizobium japonicum δ-aminolevulinic acid dehydratase with an altered metal requirement functions in situ for tetrapyrrole synthesis in soybean root nodules. *Journal of biological chemistry* **270**, 19823–19827.

50. Zapata C, Paillavil B, Chávez R, Álamos P, Levicán G. 2017 Cytochrome c peroxidase (CcP) is a molecular determinant of the oxidative stress response in the extreme acidophilic Leptospirillum sp. CF-1. *FEMS microbiology ecology* **93**.

51. Liang N, Pielak GJ, Mauk AG, Smith M, Hoffman BM. 1987 Yeast cytochrome c with phenylalanine or tyrosine at position 87 transfers electrons to (zinc cytochrome c peroxidase)+ at a rate ten thousand times that of the serine-87 or glycine-87 variants. *Proceedings of the National Academy of Sciences* **84**, 1249–1252.

52. Baral PK, Jajčanin-Jozić N, Deller S, Macheroux P, Abramić M, Gruber K. 2008 The first structure of dipeptidyl-peptidase III provides insight into the catalytic mechanism and mode of substrate binding. *Journal of Biological Chemistry* **283**, 22316–22324.

53. Clais S *et al.* 2014 Importance of biofilm formation and dipeptidyl peptidase IV for the pathogenicity of clinical Porphyromonas gingivalis isolates. *Pathogens and disease* **70**, 408–413.

54. Lodish H, Berk A, Zipursky SL, Matsudaira P, Baltimore D, Darnell J. 2000 The Role of topoisomerases in DNA replication. In *Molecular Cell Biology. 4th edition*, WH Freeman.

55. Garcia-Diaz M, Bebenek K. 2007 Multiple functions of DNA polymerases. *Critical reviews in plant sciences* **26**, 105–122.

56. Leu FP, Hingorani MM, Turner J, O’Donnell M. 2000 The δ subunit of DNA polymerase III holoenzyme serves as a sliding clamp unloader in Escherichia coli. *Journal of Biological Chemistry* **275**, 34609–34618.

57. Ebright RH, Busby S. 1995 The Escherichia coli RNA polymerase α subunit: structure and function. *Current opinion in genetics & development* **5**, 197–203.

58. Hu S-H, Peek JA, Rattigan E, Taylor RK, Martin JL. 1997 Structure of TcpG, the DsbA protein folding catalyst from Vibrio cholerae. *Journal of molecular biology* **268**, 137–146.

59. Ferri-Fioni M-L, Schmitt E, Soutourina J, Plateau P, Mechulam Y, Blanquet S. 2001 Structure of Crystallined-Tyr-tRNATyr Deacylase A REPRESENTATIVE OF A NEW CLASS OF tRNA-DEPENDENT HYDROLASES. *Journal of Biological Chemistry* **276**, 47285–47290.

60. Wei C, Jiang W, Zhao M, Ling J, Zeng X, Deng J, Jin D, Dow JM, Sun W. 2016 A systematic analysis of the role of GGDEF-EAL domain proteins in virulence and motility in Xanthomonas oryzae pv. oryzicola. *Scientific Reports* **6**. (doi:10.1038/srep23769)

61. Savelsbergh A, Rodnina M V, Wintermeyer W. 2009 Distinct functions of elongation factor G in ribosome recycling and translocation. *Rna* **15**, 772–780.

62. Tsuge T, Fukui T, Matsusaki H, Taguchi S, Kobayashi G, Ishizaki A, Doi Y. 2000 Molecular cloning of two (R)-specific enoyl-CoA hydratase genes from Pseudomonas aeruginosa and their use for polyhydroxyalkanoate synthesis. *FEMS microbiology letters* **184**, 193–198.

63. Hsiao Y-Y, Fang W-H, Lee C-C, Chen Y-P, Yuan HS. 2014 Structural insights Into DNA repair by RNase T—an exonuclease processing 3′ end of structured DNA in repair pathways. *PLoS biology* **12**, e1001803.

64. Gandía-Herrero F, García-Carmona F. 2014 Escherichia coli protein YgiD produces the structural unit of plant pigments betalains: characterization of a prokaryotic enzyme with DOPA-extradiol-dioxygenase activity. *Applied microbiology and biotechnology* **98**, 1165–1174.

65. Dym O, Eisenberg D. 2001 Sequence‐structure analysis of FAD‐containing proteins. *Protein Science* **10**, 1712–1728.

66. Fillat MF. 2014 The FUR (ferric uptake regulator) superfamily: diversity and versatility of key transcriptional regulators. *Archives of biochemistry and biophysics* **546**, 41–52.

67. Havukainen H, Haataja S, Kauko A, Pulliainen AT, Salminen A, Haikarainen T, Finne J, Papageorgiou AC. 2008 Structural basis of the zinc‐and terbium‐mediated inhibition of ferroxidase activity in Dps ferritin‐like proteins. *Protein Science* **17**, 1513–1521.

68. Zhang Y, Zagnitko O, Rodionova I, Osterman A, Godzik A. 2011 The FGGY carbohydrate kinase family: insights into the evolution of functional specificities. *PLoS computational biology* **7**, e1002318.

69. Kataeva IA, Seidel RD, Shah A, West LT, Li X-L, Ljungdahl LG. 2002 The fibronectin type 3-like repeat from the Clostridium thermocellum cellobiohydrolase CbhA promotes hydrolysis of cellulose by modifying its surface. *Appl. Environ. Microbiol.* **68**, 4292–4300.

70. Ünal CM, Steinert M. 2014 Microbial peptidyl-prolyl cis/trans isomerases (PPIases): virulence factors and potential alternative drug targets. *Microbiology and Molecular Biology Reviews* **78**, 544–571.

71. Riboldi-Tunnicliffe A, König B, Jessen S, Weiss MS, Rahfeld J, Hacker J, Fischer G, Hilgenfeld R. 2001 Crystal structure of Mip, a prolylisomerase from Legionella pneumophila. *Nature Structural & Molecular Biology* **8**, 779.

72. Neckers L, Tatu U. 2008 Molecular chaperones in pathogen virulence: emerging new targets for therapy. *Cell host & microbe* **4**, 519–527.

73. Cooper SJ, Leonard GA, McSweeney SM, Thompson AW, Naismith JH, Qamar S, Plater A, Berry A, Hunter WN. 1996 The crystal structure of a class II fructose-1, 6-bisphosphate aldolase shows a novel binuclear metal-binding active site embedded in a familiar fold. *Structure* **4**, 1303–1315.

74. Korolev S, Skarina T, Evdokimova E, Beasley S, Edwards A, Joachimiak A, Savchenko A. 2002 Crystal structure of glutamine amidotransferase from Thermotoga maritima. *Proteins* **49**, 420.

75. Kneidinger B, Graninger M, Adam G, Puchberger M, Kosma P, Zayni S, Messner P. 2001 Identification of two GDP-6-deoxy-D-lyxo-4-hexulose reductases synthesizing GDP-D-rhamnose in Aneurinibacillus thermoaerophilus L420-91T. *Journal of Biological Chemistry* **276**, 5577–5583.

76. Ryan RP, McCarthy Y, Andrade M, Farah CS, Armitage JP, Dow JM. 2010 Cell-cell signal-dependent dynamic interactions between HD-GYP and GGDEF domain proteins mediate virulence in Xanthomonas campestris. *Proceedings of the National Academy of Sciences* **107**, 5989–5994. (doi:10.1073/pnas.0912839107)

77. BRITTON KL, BAKER PJ, RICE DW, STILLMAN TJ. 1992 Structural relationship between the hexameric and tetrameric family of glutamate dehydrogenases. *European journal of biochemistry* **209**, 851–859.

78. Goenrich M, Bartoschek S, Hagemeier CH, Griesinger C, Vorholt JA. 2002 A glutathione-dependent formaldehyde-activating enzyme (Gfa) from Paracoccus denitrificans detected and purified via two-dimensional proton exchange NMR spectroscopy. *Journal of biological chemistry* **277**, 3069–3072.

79. Piszczatowski RT, Rafferty BJ, Rozado A, Tobak S, Lents NH. 2014 The glyceraldehyde 3-phosphate dehydrogenase gene (GAPDH) is regulated by myeloid zinc finger 1 (MZF-1) and is induced by calcitriol. *Biochemical and biophysical research communications* **451**, 137–141.

80. Faure D. 2002 The family-3 glycoside hydrolases: from housekeeping functions to host-microbe interactions. *Appl. Environ. Microbiol.* **68**, 1485–1490.

81. Zhou J *et al.* 2017 Distinctive molecular and biochemical characteristics of a glycoside hydrolase family 20 β-N-acetylglucosaminidase and salt tolerance. *BMC biotechnology* **17**, 37.

82. Schröder C, Blank S, Antranikian G. 2015 First glycoside hydrolase family 2 enzymes from Thermus antranikianii and Thermus brockianus with β-glucosidase activity. *Frontiers in bioengineering and biotechnology* **3**, 76.

83. Hématy K, Cherk C, Somerville S. 2009 Host-pathogen warfare at the plant cell wall. *Current Opinion in Plant Biology*. **12**, 406–413. (doi:10.1016/j.pbi.2009.06.007)

84. Okuyama M, Yoshida T, Hondoh H, Mori H, Yao M, Kimura A. 2014 Catalytic role of the calcium ion in GH97 inverting glycoside hydrolase. *FEBS Letters* **588**, 3213–3217. (doi:10.1016/j.febslet.2014.07.002)

85. Armstrong RN. 2000 Mechanistic diversity in a metalloenzyme superfamily. *Biochemistry* **39**, 13625–13632.

86. Nash HM, Bruner SD, Schärer OD, Kawate T, Addona TA, Spooner E, Lane WS, Verdine GL. 1996 Cloning of a yeast 8-oxoguanine DNA glycosylase reveals the existence of a base-excision DNA-repair protein superfamily. *Current Biology* **6**, 968–980.

87. Teng H, Grubmeyer C. 1999 Mutagenesis of histidinol dehydrogenase reveals roles for conserved histidine residues. *Biochemistry* **38**, 7363–7371.

88. Lombardi PM, Cole KE, Dowling DP, Christianson DW. 2011 Structure, mechanism, and inhibition of histone deacetylases and related metalloenzymes. *Current Opinion in Structural Biology*. **21**, 735–743. (doi:10.1016/j.sbi.2011.08.004)

89. Bardaweel S, Ghosh B, Chou T-F, Sadowsky MJ, Wagner CR. 2011 E. coli histidine triad nucleotide binding protein 1 (ecHinT) is a catalytic regulator of D-alanine dehydrogenase (DadA) activity in vivo. *PloS one* **6**, e20897.

90. Plener L, Boistard P, González A, Boucher C, Genin S. 2012 Metabolic adaptation of Ralstonia solanacearum during plant infection: a methionine biosynthesis case study. *PLoS One* **7**, e36877.

91. Linke K, Wolfram T, Bussemer J, Jakob U. 2003 The Roles of the Two Zinc Binding Sites in DnaJ. *Journal of Biological Chemistry* (doi:10.1074/jbc.M307491200)

92. O’Young J, Sukdeo N, Honek JF. 2007 Escherichia coli glyoxalase II is a binuclear zinc-dependent metalloenzyme. *Archives of biochemistry and biophysics* **459**, 20–26.

93. Dietl A-M, Amich J, Leal S, Beckmann N, Binder U, Beilhack A, Pearlman E, Haas H. 2016 Histidine biosynthesis plays a crucial role in metal homeostasis and virulence of Aspergillus fumigatus. *Virulence* **7**, 465–476.

94. Berridge MJ, Irvine RF. 1989 Inositol phosphates and cell signalling. *Nature* **341**, 197.

95. Agúndez L, MacHón C, Elvira César C, Rosa-Garrido M, Delgado MD, Llosa M. 2011 Nuclear targeting of a bacterial integrase that mediates site-specific recombination between bacterial and human target sequences. *Applied and Environmental Microbiology* **77**, 201–210. (doi:10.1128/AEM.01371-10)

96. Semavina M, Beckett D, Logan TM. 2006 Metal-linked dimerization in the iron-dependent regulator from Mycobacterium tuberculosis. *Biochemistry* **45**, 12480–12490.

97. Imada K, Sato M, Tanaka N, Katsube Y, Matsuura Y, Oshima T. 1991 Three-dimensional structure of a highly thermostable enzyme, 3-isopropylmalate dehydrogenase of Thermus thermophilus at 2.2 Å resolution. *Journal of molecular biology* **222**, 725–738.

98. Nguyen CC, Saier Jr MH. 1995 Phylogenetic, structural and functional analyses of the LacI-GalR family of bacterial transcription factors. *FEBS letters* **377**, 98–102.

99. Betha AK, Williams AM, Martinis SA. 2007 Isolated CP1 domain of Escherichia coli leucyl-tRNA synthetase is dependent on flanking hinge motifs for amino acid editing activity. *Biochemistry* **46**, 6258–6267.

100. Wang X-G, Olsen LR, Roderick SL. 2002 Structure of the lac operon galactoside acetyltransferase. *Structure* **10**, 581–588.

101. Rawlings ND, Barrett AJ. 1995 Evolutionary families of metallopeptidases. *Methods in Enzymology* **248**, 183–228. (doi:10.1016/0076-6879(95)48015-3)

102. Brown NL, Stoyanov J V., Kidd SP, Hobman JL. 2003 The MerR family of transcriptional regulators. *FEMS Microbiology Reviews*. **27**, 145–163. (doi:10.1016/S0168-6445(03)00051-2)

103. Seibert CM, Raushel FM. 2005 Structural and catalytic diversity within the amidohydrolase superfamily. *Biochemistry* **44**, 6383–6391.

104. Palzkill T. 2013 Metallo‐β‐lactamase structure and function. *Annals of the New York Academy of Sciences* **1277**, 91–104.

105. Ali F, Yao Z, Li W, Lina S, Lin W, Lin X. 2017 LuxS quorum sensing system, its protein modeling and active-binding sites and phylogenetic analysis from Aeromonas hydrophila.

106. Metz AH, Hollis T, Eichman BF. 2007 DNA damage recognition and repair by 3‐methyladenine DNA glycosylase I (TAG). *The EMBO journal* **26**, 2411–2420.

107. Yim L, Martínez-Vicente M, Villarroya M, Aguado C, Knecht E, Armengod M-E. 2003 The GTPase activity and C-terminal cysteine of the Escherichia coli MnmE protein are essential for its tRNA modifying function. *Journal of Biological Chemistry* **278**, 28378–28387.

108. Schirner K, Eun Y-J, Dion M, Luo Y, Helmann JD, Garner EC, Walker S. 2015 Lipid-linked cell wall precursors regulate membrane association of bacterial actin MreB. *Nature chemical biology* **11**, 38.

109. Bolz NJ, Lenhart JS, Weindorf SC, Simmons LA. 2012 Residues in the N-terminal domain of MutL required for mismatch repair in bacillus subtilis. *Journal of Bacteriology* **194**, 5361–5367. (doi:10.1128/JB.01142-12)

110. Kerff F *et al.* 2010 Specific structural features of the N-acetylmuramoyl-L-alanine amidase AmiD from Escherichia coli and mechanistic implications for enzymes of this family. *Journal of molecular biology* **397**, 249–259.

111. Allard STM, Giraud M-F, Naismith JH. 2001 Epimerases: structure, function and mechanism. *Cellular and Molecular Life Sciences CMLS* **58**, 1650–1665.

112. McLennan AG. 2006 The Nudix hydrolase superfamily. *Cellular and Molecular Life Sciences CMLS* **63**, 123–143.

113. Kenney LJ. 2002 Structure/function relationships in OmpR and other winged-helix transcription factors. *Current opinion in microbiology* **5**, 135–141.

114. Barbas JA, Díaz J, Rodríguez-Tébar A, Vázquez D. 1986 Specific location of penicillin-binding proteins within the cell envelope of Escherichia coli. *Journal of bacteriology* **165**, 269–75.

115. Massova I, Mobashery S. 1998 Kinship and diversification of bacterial penicillin-binding proteins and ??-lactamases. *Antimicrobial Agents and Chemotherapy*. **42**, 1–17.

116. Peer WA. 2011 The role of multifunctional M1 metallopeptidases in cell cycle progression. *Annals of botany* **107**, 1171–1181.

117. Yang J-Y *et al.* 2016 Characterization of a New M13 Metallopeptidase from Deep-Sea Shewanella sp. E525-6 and Mechanistic Insight into Its Catalysis. *Frontiers in microbiology* **6**, 1498.

118. Martínez-Rodríguez S, García-Pino A, Las Heras-Vázquez FJ, Clemente-Jiménez JM, Rodríguez-Vico F, García-Ruiz JM, Loris R, Gavira JA. 2012 Mutational and structural analysis of LN-carbamoylase reveals new insights into a peptidase M20/M25/M40 family member. *Journal of bacteriology* **194**, 5759–5768.

119. Ettema TJG, Brinkman AB, Tani TH, Rafferty JB, Van der Oost J. 2002 A novel ligand-binding domain involved in regulation of amino acid metabolism in prokaryotes. *Journal of Biological Chemistry* **277**, 37464–37468.

120. Hecht KA, Wytiaz VA, Ast T, Schuldiner M, Brodsky JL. 2013 Characterization of an M28 metalloprotease family member residing in the yeast vacuole. *FEMS yeast research* **13**, 471–484.

121. Jang H Bin *et al.* 2012 RNA-Seq-Based Metatranscriptomic and Microscopic Investigation Reveals Novel Metalloproteases of Neobodo sp. as Potential Virulence Factors for Soft Tunic Syndrome in Halocynthia roretzi. *PLoS ONE* **7**. (doi:10.1371/journal.pone.0052379)

122. Grabowska M, Jagielska E, Czapinska H, Bochtler M, Sabala I. 2015 High resolution structure of an M23 peptidase with a substrate analogue. *Scientific Reports* (doi:10.1038/srep14833)

123. Zaher HS, Green R. 2011 A primary role for release factor 3 in quality control during translation elongation in Escherichia coli. *Cell* **147**, 396–408.

124. Horler RSP, Müller A, Williamson DC, Potts JR, Wilson KS, Thomas GH. 2009 Furanose-specific sugar transport characterization of a bacterial galactofuranose-binding protein. *Journal of Biological Chemistry* **284**, 31156–31163.

125. Beyer D *et al.* 2004 New class of bacterial phenylalanyl-tRNA synthetase inhibitors with high potency and broad-spectrum activity. *Antimicrobial agents and chemotherapy* **48**, 525–532.

126. Sanfélix‐Haywood N, Coll‐Marqués JM, Yebra MJ. 2011 Role of α‐phosphoglucomutase and phosphoglucose isomerase activities at the branching point between sugar catabolism and anabolism in Lactobacillus casei. *Journal of applied microbiology* **111**, 433–442.

127. Roujeinikova A, Baldock C, Simon WJ, Gilroy J, Baker PJ, Stuitje AR, Rice DW, Slabas AR, Rafferty JB. 2002 X-ray crystallographic studies on butyryl-ACP reveal flexibility of the structure around a putative acyl chain binding site. *Structure* **10**, 825–835.

128. Chiang P, Sampaleanu LM, Ayers M, Pahuta M, Howell PL, Burrows LL. 2008 Functional role of conserved residues in the characteristic secretion NTPase motifs of the Pseudomonas aeruginosa type IV pilus motor proteins PilB, PilT and PilU. *Microbiology* **154**, 114–126.

129. Bandow JE, Becher D, Büttner K, Hochgräfe F, Freiberg C, Brötz H, Hecker M. 2003 The role of peptide deformylase in protein biosynthesis: a proteomic study. *Proteomics* **3**, 299–306.

130. Blair DE, Schüttelkopf AW, MacRae JI, van Aalten DMF. 2005 Structure and metal-dependent mechanism of peptidoglycan deacetylase, a streptococcal virulence factor. *Proceedings of the National Academy of Sciences* **102**, 15429–15434.

131. Iyer LM, Abhiman S, Burroughs AM, Aravind L. 2009 Amidoligases with ATP-grasp, glutamine synthetase-like and acetyltransferase-like domains: synthesis of novel metabolites and peptide modifications of proteins. *Molecular bioSystems* **5**, 1636–1660.

132. Kaushik S, Sowdhamini R. 2014 Distribution, classification, domain architectures and evolution of prolyl oligopeptidases in prokaryotic lineages. *BMC genomics* **15**, 985.

133. McQueen DA, Schottel JL. 1987 Purification and characterization of a novel extracellular esterase from pathogenic Streptomyces scabies that is inducible by zinc. *Journal of bacteriology* **169**, 1967–1971.

134. Rocha ER, Tzianabos AO, Smith CJ. 2007 Thioredoxin reductase is essential for thiol/disulfide redox control and oxidative stress survival of the anaerobe Bacteroides fragilis. *Journal of bacteriology* **189**, 8015–8023.

135. Russel M, Model P. 1988 Sequence of thioredoxin reductase from Escherichia coli. Relationship to other flavoprotein disulfide oxidoreductases. *Journal of Biological Chemistry* **263**, 9015–9019.

136. Yang Y, Tsui H-CT, Man T-K, Winkler ME. 1998 Identification and function of the pdxY gene, which encodes a novel pyridoxal kinase involved in the salvage pathway of pyridoxal 5′-phosphate biosynthesis in Escherichia coli K-12. *Journal of bacteriology* **180**, 1814–1821.

137. Kittendorf JD, Barcomb LM, Nonekowski ST, Garcia GA. 2001 tRNA− Guanine Transglycosylase from Escherichia coli: Molecular Mechanism and Role of Aspartate 89. *Biochemistry* **40**, 14123–14133.

138. Wang S. 2012 Bacterial Two-Component Systems : Structures and Signaling Mechanisms. *Protein Phosphorylation in Human Health, Dr. Cai Huang (Ed.)* , 439–466. (doi:10.5772/2944)

139. Gao R, Mack TR, Stock AM. 2007 Bacterial response regulators: versatile regulatory strategies from common domains. *Trends in Biochemical Sciences*. **32**, 225–234. (doi:10.1016/j.tibs.2007.03.002)

140. Zuo Y, Wang Y, Malhotra A. 2005 Crystal structure of Escherichia coli RNase D, an exoribonuclease involved in structured RNA processing. *Structure* **13**, 973–984.

141. Torrents E. 2014 Ribonucleotide reductases: essential enzymes for bacterial life. *Frontiers in cellular and infection microbiology* **4**, 52.

142. Chandler JR *et al.* 2012 Bactobolin resistance is conferred by mutations in the L2 ribosomal protein. *mBio* **3**. (doi:10.1128/mBio.00499-12)

143. Kornder JD. 2002 Streptomycin revisited: Molecular action in the microbial cell. *Medical Hypotheses*. **58**, 34–46. (doi:10.1054/mehy.2001.1450)

144. Giraud M-F, Leonard GA, Field RA, Berlind C, Naismith JH. 2000 RmlC, the third enzyme of dTDP-L-rhamnose pathway, is a new class of epimerase. *Nature Structural and Molecular Biology* **7**, 398.

145. Matos RG, Bárria C, Moreira RN, Barahona S, Domingues S, Arraiano CM. 2014 The importance of proteins of the RNase II/RNB-family in pathogenic bacteria. *Frontiers in cellular and infection microbiology* **4**, 68.

146. Büttner K, Wenig K, Hopfner KP. 2005 Structural framework for the mechanism of archaeal exosomes in RNA processing. *Molecular Cell* **20**, 461–471. (doi:10.1016/j.molcel.2005.10.018)

147. Lee W-Y, Brune DC, Lobrutto R, Blankenship RE. 1995 Isolation, characterization, and primary structure of rubredoxin from the photosynthetic bacterium, Heliobacillus mobilis. *Archives of biochemistry and biophysics* **318**, 80–88.

148. Desai NA, Shankar V. 2003 Single-strand-specific nucleases. *FEMS microbiology reviews* **26**, 457–491.

149. Zhang Y, Mühlen S, Oates C V, Pearson JS, Hartland EL. 2016 Identification of a distinct substrate-binding domain in the bacterial cysteine methyltransferase effectors NleE and OspZ. *Journal of Biological Chemistry* **291**, 20149–20162.

150. Lill R, Cunningham K, Brundage LA, Ito K, Oliver D, Wickner W. 1989 SecA protein hydrolyzes ATP and is an essential component of the protein translocation ATPase of Escherichia coli. *The EMBO journal* **8**, 961–966.

151. Alvarez-Venegas R. 2014 Bacterial SET domain proteins and their role in eukaryotic chromatin modification. *Frontiers in genetics* **5**, 65.

152. Michel G, Roszak AW, Sauvé V, Maclean J, Matte A, Coggins JR, Cygler M, Lapthorn AJ. 2003 Structures of shikimate dehydrogenase aroe and its paralog ydib a common structural framework for different activities. *Journal of Biological Chemistry* **278**, 19463–19472.

153. Kavanagh KL, Jörnvall H, Persson B, Oppermann U. 2008 Medium- and short-chain dehydrogenase/reductase gene and protein families: The SDR superfamily: Functional and structural diversity within a family of metabolic and regulatory enzymes. *Cellular and Molecular Life Sciences*. **65**, 3895–3906. (doi:10.1007/s00018-008-8588-y)

154. Pumirat P, Vanaporn M, Pinweha P, Tandhavanant S, Korbsrisate S, Chantratita N. 2014 The role of short-chain dehydrogenase/oxidoreductase, induced by salt stress, on host interaction of B. pseudomallei. *BMC microbiology* **14**, 1.

155. Steenbergen SM, Lee Y-C, Vann WF, Vionnet J, Wright LF, Vimr ER. 2006 Separate pathways for O acetylation of polymeric and monomeric sialic acids and identification of sialyl O-acetyl esterase in Escherichia coli K1. *Journal of bacteriology* **188**, 6195–6206.

156. Kazmierczak MJ, Wiedmann M, Boor KJ. 2005 Alternative sigma factors and their roles in bacterial virulence. *Microbiology and Molecular Biology Reviews* **69**, 527–543. (doi:10.1128/MMBR.69.4.527)

157. Akopian D, Shen K, Zhang X, Shan S. 2013 Signal recognition particle: an essential protein-targeting machine. *Annual review of biochemistry* **82**, 693–721.

158. Fraser ME, James MNG, Bridger WA, Wolodko WT. 1999 A detailed structural description of Escherichia coli succinyl-CoA synthetase. *Journal of molecular biology* **285**, 1633–1653.

159. Eschenbrenner M, Covès J, Fontecave M. 1995 NADPH-sulfite reductase flavoprotein from Escherichia coli: contribution to the flavin content and subunit interaction. *FEBS letters* **374**, 82–84.

160. Chen YC, Li CL, Hsiao YY, Duh Y, Yuan HS. 2014 Structure and function of TatD exonuclease in DNA repair. *Nucleic Acids Research* **42**, 10776–10785. (doi:10.1093/nar/gku732)

161. Lehmann C, Begley TP, Ealick SE. 2006 Structure of the Escherichia coli ThiS− ThiF Complex, a Key Component of the Sulfur Transfer System in Thiamin Biosynthesis. *Biochemistry* **45**, 11–19.

162. Andersen GR, Nissen P, Nyborg J. 2003 Elongation factors in protein biosynthesis. *Trends in Biochemical Sciences*. **28**, 434–441. (doi:10.1016/S0968-0004(03)00162-2)

163. Kapralou S, Fabbretti A, Garulli C, Spurio R, Gualerzi CO, Dahlberg AE, Pon CL. 2008 Translation initiation factor IF1 of Bacillus stearothermophilus and Thermus thermophilus substitute for Escherichia coli IF1 in vivo and in vitro without a direct IF1–IF2 interaction. *Molecular microbiology* **70**, 1368–1377.

164. Wyckoff TJO, Raetz CRH. 1999 The Active Site of Escherichia coliUDP-N-acetylglucosamine Acyltransferase CHEMICAL MODIFICATION AND SITE-DIRECTED MUTAGENESIS. *Journal of Biological Chemistry* **274**, 27047–27055.

165. Hurdle JG, O’Neill AJ, Chopra I. 2005 Prospects for aminoacyl-tRNA synthetase inhibitors as new antimicrobial agents. *Antimicrobial agents and chemotherapy* **49**, 4821–4833.

166. Reader JS, Ordoukhanian PT, Kim JC, De Crécy-Lagard V, Hwang I, Farrand S, Schimmel P. 2005 Virology: Major biocontrol of plant tumors targets tRNA synthetase. *Science* **309**, 1533. (doi:10.1126/science.1116841)

167. Woese CR, Olsen GJ, Ibba M, Söll D. 2000 Aminoacyl-tRNA synthetases, the genetic code, and the evolutionary process. *Microbiol. Mol. Biol. Rev.* **64**, 202–236.

168. Rao DN, Dryden DTF, Bheemanaik S. 2013 Type III restriction-modification enzymes: a historical perspective. *Nucleic acids research* **42**, 45–55.

169. Van Houten B, Snowden A. 1993 Mechanism of action of the Escherichia coli UvrABC nuclease: clues to the damage recognition problem. *Bioessays* **15**, 51–59.

170. Fan J, Liu Q, Hao Q, Teng M, Niu L. 2007 Crystal structure of uroporphyrinogen decarboxylase from Bacillus subtilis. *Journal of bacteriology* **189**, 3573–3580.

171. Croteau DL, DellaVecchia MJ, Wang H, Bienstock RJ, Melton MA, Van Houten B. 2006 The C-terminal zinc finger of UvrA does not bind DNA directly but regulates damage-specific DNA binding. *Journal of Biological Chemistry* **281**, 26370–26381. (doi:10.1074/jbc.M603093200)

172. Aravind L, Walker DR, Koonin E V. 1999 Conserved domains in DNA repair proteins and evolution of repair systems. *Nucleic acids research* **27**, 1223–1242.

173. Caldas T, Malki A, Kern R, Abdallah J, Richarme G. 2006 The Escherichia coli thioredoxin homolog YbbN/Trxsc is a chaperone and a weak protein oxidoreductase. *Biochemical and biophysical research communications* **343**, 780–786.

174. Banecki B, Wawrzynow A, Puzewicz J, Georgopoulos C, Zylicz M. 2001 Structure-Function Analysis of the Zinc-binding Region of the ClpX Molecular Chaperone. *Journal of Biological Chemistry* **276**, 18843–18848. (doi:10.1074/jbc.M007507200)

**Supplementary material, table S4: GO Biological process terms of putative Zn-binding proteins of *Xtu***

| S. No. | GO Biological process ID | GO Biological process Term | GO biological process Groups (Based on kappa score) | Neighboring Genes | Degree/ Links of the GO term | Associated Genes Found in particular GO term |
| --- | --- | --- | --- | --- | --- | --- |
| 1 | GO:0005975 | carbohydrate metabolic process | [Group00] | 10 | 10 | [FD63_RS01790, FD63_RS02025, FD63_RS07060, FD63_RS13230, FD63_RS14410, FD63_RS15020, FD63_RS15190, FD63_RS15970, FD63_RS17605, FD63_RS18685] |
| 2 | GO:1901135 | carbohydrate derivative metabolic process | [Group01] | 7 | 9 | [FD63_RS01935, FD63_RS04480, FD63_RS04805, FD63_RS15190, FD63_RS15980, FD63_RS15995, FD63_RS18060] |
| 3 | GO:1901137 | carbohydrate derivative biosynthetic process | [Group01] | 4 | 8 | [FD63_RS04480, FD63_RS04805, FD63_RS15980, FD63_RS18060] |
| 4 | GO:0044036 | cell wall macromolecule metabolic process | [Group01] | 3 | 5 | [FD63_RS04480, FD63_RS04805, FD63_RS18685] |
| 5 | GO:0043170 | macromolecule metabolic process | [Group02, Group07, Group12] | 42 | 57 | [FD63_RS00430, FD63_RS01125, FD63_RS01330, FD63_RS01865, FD63_RS02450, FD63_RS02640, FD63_RS02920, FD63_RS03805, FD63_RS04035, FD63_RS04480, FD63_RS04540, FD63_RS04775, FD63_RS04805, FD63_RS05020, FD63_RS05925, FD63_RS06120, FD63_RS06445, FD63_RS06450, FD63_RS06850, FD63_RS06915, FD63_RS07895, FD63_RS08075, FD63_RS08095, FD63_RS08365, FD63_RS09220, FD63_RS10745, FD63_RS11575, FD63_RS12400, FD63_RS12830, FD63_RS14425, FD63_RS14595, FD63_RS14945, FD63_RS15535, FD63_RS16210, FD63_RS16230, FD63_RS17735, FD63_RS18060, FD63_RS18175, FD63_RS18300, FD63_RS18585, FD63_RS18685, hflB] |
| 6 | GO:0019538 | protein metabolic process | [Group02, Group07] | 20 | 28 | [FD63_RS00430, FD63_RS01125, FD63_RS01330, FD63_RS01865, FD63_RS02920, FD63_RS03805, FD63_RS04540, FD63_RS04805, FD63_RS05925, FD63_RS06445, FD63_RS06450, FD63_RS06915, FD63_RS08365, FD63_RS11575, FD63_RS12400, FD63_RS14595, FD63_RS16210, FD63_RS17735, FD63_RS18300, hflB] |
| 7 | GO:0006508 | proteolysis | [Group02, Group07] | 16 | 20 | [FD63_RS00430, FD63_RS01125, FD63_RS01330, FD63_RS01865, FD63_RS02920, FD63_RS04540, FD63_RS04805, FD63_RS06445, FD63_RS06450, FD63_RS06915, FD63_RS12400, FD63_RS14595, FD63_RS16210, FD63_RS17735, FD63_RS18300, hflB] |
| 8 | GO:1901564 | organonitrogen compound metabolic process | [Group02] | 30 | 35 | [FD63_RS00430, FD63_RS01125, FD63_RS01330, FD63_RS01365, FD63_RS01865, FD63_RS01935, FD63_RS02920, FD63_RS03475, FD63_RS03805, FD63_RS04480, FD63_RS04540, FD63_RS04805, FD63_RS05760, FD63_RS05925, FD63_RS06075, FD63_RS06445, FD63_RS06450, FD63_RS06915, FD63_RS08235, FD63_RS08365, FD63_RS10450, FD63_RS11575, FD63_RS12400, FD63_RS12505, FD63_RS14240, FD63_RS14595, FD63_RS16210, FD63_RS17735, FD63_RS18300, hflB] |
| 9 | GO:0030258 | lipid modification | [Group03, Group04, Group11] | 4 | 13 | [FD63_RS05600, FD63_RS07060, FD63_RS09110, FD63_RS13185] |
| 10 | GO:1901575 | organic substance catabolic process | [Group03, Group11] | 8 | 13 | [FD63_RS03475, FD63_RS05600, FD63_RS07060, FD63_RS09110, FD63_RS14425, FD63_RS15085, FD63_RS18685, hflB] |
| 11 | GO:0044282 | small molecule catabolic process | [Group03, Group11] | 4 | 11 | [FD63_RS05600, FD63_RS07060, FD63_RS09110, FD63_RS15085] |
| 12 | GO:0044248 | cellular catabolic process | [Group03, Group11] | 5 | 10 | [FD63_RS03475, FD63_RS05600, FD63_RS09110, FD63_RS14425, FD63_RS15085] |
| 13 | GO:0009057 | macromolecule catabolic process | [Group03] | 3 | 6 | [FD63_RS14425, FD63_RS18685, hflB] |
| 14 | GO:0019637 | organophosphate metabolic process | [Group04, Group11] | 5 | 11 | [FD63_RS01935, FD63_RS07060, FD63_RS08235, FD63_RS13185, FD63_RS18060] |
| 15 | GO:0006796 | phosphate-containing compound metabolic process | [Group04] | 7 | 12 | [FD63_RS01935, FD63_RS07060, FD63_RS08235, FD63_RS10150, FD63_RS10450, FD63_RS13185, FD63_RS18060] |
| 16 | GO:0006793 | phosphorus metabolic process | [Group04] | 7 | 12 | [FD63_RS01935, FD63_RS07060, FD63_RS08235, FD63_RS10150, FD63_RS10450, FD63_RS13185, FD63_RS18060] |
| 17 | GO:0016310 | phosphorylation | [Group04] | 6 | 11 | [FD63_RS01935, FD63_RS07060, FD63_RS08235, FD63_RS10150, FD63_RS10450, FD63_RS13185] |
| 18 | GO:1901615 | organic hydroxy compound metabolic process | [Group04] | 3 | 5 | [FD63_RS07060, FD63_RS08235, FD63_RS15095] |
| 19 | GO:0044267 | cellular protein metabolic process | [Group05] | 4 | 13 | [FD63_RS03805, FD63_RS05925, FD63_RS08365, FD63_RS11575] |
| 20 | GO:0006464 | cellular protein modification process | [Group05] | 4 | 13 | [FD63_RS03805, FD63_RS05925, FD63_RS08365, FD63_RS11575] |
| 21 | GO:0036211 | protein modification process | [Group05] | 4 | 13 | [FD63_RS03805, FD63_RS05925, FD63_RS08365, FD63_RS11575] |
| 22 | GO:0043412 | macromolecule modification | [Group05] | 4 | 12 | [FD63_RS03805, FD63_RS05925, FD63_RS08365, FD63_RS11575] |
| 23 | GO:0018193 | peptidyl-amino acid modification | [Group05] | 4 | 12 | [FD63_RS03805, FD63_RS05925, FD63_RS08365, FD63_RS11575] |
| 24 | GO:0018208 | peptidyl-proline modification | [Group05] | 4 | 12 | [FD63_RS03805, FD63_RS05925, FD63_RS08365, FD63_RS11575] |
| 25 | GO:0000413 | protein peptidyl-prolyl isomerization | [Group05] | 4 | 11 | [FD63_RS03805, FD63_RS05925, FD63_RS08365, FD63_RS11575] |
| 26 | GO:0006139 | nucleobase-containing compound metabolic process | [Group06, Group07, Group12] | 23 | 67 | [FD63_RS01935, FD63_RS02450, FD63_RS02640, FD63_RS04035, FD63_RS04775, FD63_RS05020, FD63_RS06120, FD63_RS06850, FD63_RS07895, FD63_RS08075, FD63_RS08095, FD63_RS09220, FD63_RS10745, FD63_RS12830, FD63_RS14425, FD63_RS14945, FD63_RS15535, FD63_RS15980, FD63_RS15995, FD63_RS16230, FD63_RS18060, FD63_RS18175, FD63_RS18585] |
| 27 | GO:0090304 | nucleic acid metabolic process | [Group06, Group07, Group12] | 20 | 61 | [FD63_RS02450, FD63_RS02640, FD63_RS04035, FD63_RS04775, FD63_RS05020, FD63_RS06120, FD63_RS06850, FD63_RS07895, FD63_RS08075, FD63_RS08095, FD63_RS09220, FD63_RS10745, FD63_RS12830, FD63_RS14425, FD63_RS14945, FD63_RS15535, FD63_RS16230, FD63_RS18060, FD63_RS18175, FD63_RS18585] |
| 28 | GO:0006259 | DNA metabolic process | [Group06, Group08, Group12] | 8 | 18 | [FD63_RS05020, FD63_RS06120, FD63_RS06850, FD63_RS08095, FD63_RS12830, FD63_RS14425, FD63_RS18060, FD63_RS18585] |
| 29 | GO:0033554 | cellular response to stress | [Group06, Group08] | 5 | 15 | [FD63_RS00945, FD63_RS00950, FD63_RS05020, FD63_RS06850, FD63_RS12830] |
| 30 | GO:0006974 | cellular response to DNA damage stimulus | [Group06, Group08] | 3 | 8 | [FD63_RS05020, FD63_RS06850, FD63_RS12830] |
| 31 | GO:0006281 | DNA repair | [Group06, Group08] | 3 | 8 | [FD63_RS05020, FD63_RS06850, FD63_RS12830] |
| 32 | GO:0006260 | DNA replication | [Group06] | 3 | 6 | [FD63_RS05020, FD63_RS06120, FD63_RS18060] |
| 33 | GO:0044260 | cellular macromolecule metabolic process | [Group07, Group12] | 27 | 69 | [FD63_RS02450, FD63_RS02640, FD63_RS03805, FD63_RS04035, FD63_RS04480, FD63_RS04775, FD63_RS04805, FD63_RS05020, FD63_RS05925, FD63_RS06120, FD63_RS06850, FD63_RS07895, FD63_RS08075, FD63_RS08095, FD63_RS08365, FD63_RS09220, FD63_RS10745, FD63_RS11575, FD63_RS12830, FD63_RS14425, FD63_RS14945, FD63_RS15535, FD63_RS16230, FD63_RS18060, FD63_RS18175, FD63_RS18585, FD63_RS18685] |
| 34 | GO:0034645 | cellular macromolecule biosynthetic process | [Group07, Group12] | 17 | 60 | [FD63_RS02450, FD63_RS02640, FD63_RS04035, FD63_RS04480, FD63_RS04775, FD63_RS04805, FD63_RS05020, FD63_RS06120, FD63_RS07895, FD63_RS08075, FD63_RS09220, FD63_RS10745, FD63_RS14945, FD63_RS15535, FD63_RS16230, FD63_RS18060, FD63_RS18175] |
| 35 | GO:0009059 | macromolecule biosynthetic process | [Group07, Group12] | 17 | 59 | [FD63_RS02450, FD63_RS02640, FD63_RS04035, FD63_RS04480, FD63_RS04775, FD63_RS04805, FD63_RS05020, FD63_RS06120, FD63_RS07895, FD63_RS08075, FD63_RS09220, FD63_RS10745, FD63_RS14945, FD63_RS15535, FD63_RS16230, FD63_RS18060, FD63_RS18175] |
| 36 | GO:0098754 | detoxification | [Group08] | 3 | 11 | [FD63_RS00945, FD63_RS00950, FD63_RS15085] |
| 37 | GO:0000302 | response to reactive oxygen species | [Group08] | 3 | 11 | [FD63_RS00945, FD63_RS00950, FD63_RS03435] |
| 38 | GO:1990748 | cellular detoxification | [Group08] | 3 | 10 | [FD63_RS00945, FD63_RS00950, FD63_RS15085] |
| 39 | GO:0006979 | response to oxidative stress | [Group08] | 3 | 10 | [FD63_RS00945, FD63_RS00950, FD63_RS03435] |
| 40 | GO:1901700 | response to oxygen-containing compound | [Group08] | 3 | 10 | [FD63_RS00945, FD63_RS00950, FD63_RS03435] |
| 41 | GO:0009636 | response to toxic substance | [Group08] | 3 | 10 | [FD63_RS00945, FD63_RS00950, FD63_RS15085] |
| 42 | GO:0006812 | cation transport | [Group09] | 6 | 23 | [FD63_RS01935, FD63_RS01945, FD63_RS05235, FD63_RS05240, FD63_RS11755, FD63_RS17225] |
| 43 | GO:0006811 | ion transport | [Group09] | 7 | 23 | [FD63_RS01935, FD63_RS01945, FD63_RS04035, FD63_RS05235, FD63_RS05240, FD63_RS11755, FD63_RS17225] |
| 44 | GO:0098655 | cation transmembrane transport | [Group09] | 4 | 21 | [FD63_RS01935, FD63_RS01945, FD63_RS05235, FD63_RS05240] |
| 45 | GO:0098662 | inorganic cation transmembrane transport | [Group09] | 4 | 21 | [FD63_RS01935, FD63_RS01945, FD63_RS05235, FD63_RS05240] |
| 46 | GO:0034220 | ion transmembrane transport | [Group09] | 4 | 21 | [FD63_RS01935, FD63_RS01945, FD63_RS05235, FD63_RS05240] |
| 47 | GO:0015992 | proton transport | [Group09] | 4 | 21 | [FD63_RS01935, FD63_RS01945, FD63_RS05235, FD63_RS05240] |
| 48 | GO:0045333 | cellular respiration | [Group09] | 4 | 20 | [FD63_RS01935, FD63_RS01945, FD63_RS05235, FD63_RS05240] |
| 49 | GO:0015980 | energy derivation by oxidation of organic compounds | [Group09] | 4 | 20 | [FD63_RS01935, FD63_RS01945, FD63_RS05235, FD63_RS05240] |
| 50 | GO:0006091 | generation of precursor metabolites and energy | [Group09] | 4 | 20 | [FD63_RS01935, FD63_RS01945, FD63_RS05235, FD63_RS05240] |
| 51 | GO:1902600 | hydrogen ion transmembrane transport | [Group09] | 4 | 20 | [FD63_RS01935, FD63_RS01945, FD63_RS05235, FD63_RS05240] |
| 52 | GO:0098660 | inorganic ion transmembrane transport | [Group09] | 4 | 20 | [FD63_RS01935, FD63_RS01945, FD63_RS05235, FD63_RS05240] |
| 53 | GO:0015672 | monovalent inorganic cation transport | [Group09] | 4 | 20 | [FD63_RS01935, FD63_RS01945, FD63_RS05235, FD63_RS05240] |
| 54 | GO:0009060 | aerobic respiration | [Group09] | 4 | 19 | [FD63_RS01935, FD63_RS01945, FD63_RS05235, FD63_RS05240] |
| 55 | GO:0006818 | hydrogen transport | [Group09] | 4 | 19 | [FD63_RS01935, FD63_RS01945, FD63_RS05235, FD63_RS05240] |
| 56 | GO:0022900 | electron transport chain | [Group09] | 3 | 18 | [FD63_RS01935, FD63_RS01945, FD63_RS05240] |
| 57 | GO:0019725 | cellular homeostasis | [Group10] | 10 | 27 | [FD63_RS02225, FD63_RS03435, FD63_RS04485, FD63_RS05025, FD63_RS06100, FD63_RS11755, FD63_RS15155, FD63_RS15160, FD63_RS17225, FD63_RS18050] |
| 58 | GO:0042592 | homeostatic process | [Group10] | 10 | 26 | [FD63_RS02225, FD63_RS03435, FD63_RS04485, FD63_RS05025, FD63_RS06100, FD63_RS11755, FD63_RS15155, FD63_RS15160, FD63_RS17225, FD63_RS18050] |
| 59 | GO:0050801 | ion homeostasis | [Group10] | 4 | 21 | [FD63_RS04485, FD63_RS05025, FD63_RS11755, FD63_RS17225] |
| 60 | GO:0055065 | metal ion homeostasis | [Group10] | 4 | 21 | [FD63_RS04485, FD63_RS05025, FD63_RS11755, FD63_RS17225] |
| 61 | GO:0055080 | cation homeostasis | [Group10] | 4 | 20 | [FD63_RS04485, FD63_RS05025, FD63_RS11755, FD63_RS17225] |
| 62 | GO:0030003 | cellular cation homeostasis | [Group10] | 4 | 20 | [FD63_RS04485, FD63_RS05025, FD63_RS11755, FD63_RS17225] |
| 63 | GO:0055082 | cellular chemical homeostasis | [Group10] | 4 | 20 | [FD63_RS04485, FD63_RS05025, FD63_RS11755, FD63_RS17225] |
| 64 | GO:0006873 | cellular ion homeostasis | [Group10] | 4 | 20 | [FD63_RS04485, FD63_RS05025, FD63_RS11755, FD63_RS17225] |
| 65 | GO:0006875 | cellular metal ion homeostasis | [Group10] | 4 | 20 | [FD63_RS04485, FD63_RS05025, FD63_RS11755, FD63_RS17225] |
| 66 | GO:0046916 | cellular transition metal ion homeostasis | [Group10] | 4 | 20 | [FD63_RS04485, FD63_RS05025, FD63_RS11755, FD63_RS17225] |
| 67 | GO:0048878 | chemical homeostasis | [Group10] | 4 | 20 | [FD63_RS04485, FD63_RS05025, FD63_RS11755, FD63_RS17225] |
| 68 | GO:0098771 | inorganic ion homeostasis | [Group10] | 4 | 19 | [FD63_RS04485, FD63_RS05025, FD63_RS11755, FD63_RS17225] |
| 69 | GO:0055076 | transition metal ion homeostasis | [Group10] | 4 | 19 | [FD63_RS04485, FD63_RS05025, FD63_RS11755, FD63_RS17225] |
| 70 | GO:0006879 | cellular iron ion homeostasis | [Group10] | 4 | 18 | [FD63_RS04485, FD63_RS05025, FD63_RS11755, FD63_RS17225] |
| 71 | GO:0045454 | cell redox homeostasis | [Group10] | 6 | 10 | [FD63_RS02225, FD63_RS03435, FD63_RS06100, FD63_RS15155, FD63_RS15160, FD63_RS18050] |
| 72 | GO:0019752 | carboxylic acid metabolic process | [Group11] | 8 | 23 | [FD63_RS05600, FD63_RS05760, FD63_RS06075, FD63_RS09110, FD63_RS12505, FD63_RS14240, FD63_RS17605, fabG] |
| 73 | GO:0016053 | organic acid biosynthetic process | [Group11] | 4 | 21 | [FD63_RS06075, FD63_RS14240, FD63_RS17605, fabG] |
| 74 | GO:0006082 | organic acid metabolic process | [Group11] | 8 | 21 | [FD63_RS05600, FD63_RS05760, FD63_RS06075, FD63_RS09110, FD63_RS12505, FD63_RS14240, FD63_RS17605, fabG] |
| 75 | GO:0043436 | oxoacid metabolic process | [Group11] | 8 | 21 | [FD63_RS05600, FD63_RS05760, FD63_RS06075, FD63_RS09110, FD63_RS12505, FD63_RS14240, FD63_RS17605, fabG] |
| 76 | GO:0046394 | carboxylic acid biosynthetic process | [Group11] | 4 | 18 | [FD63_RS06075, FD63_RS14240, FD63_RS17605, fabG] |
| 77 | GO:0044255 | cellular lipid metabolic process | [Group11] | 6 | 18 | [FD63_RS05600, FD63_RS07060, FD63_RS09110, FD63_RS13185, FD63_RS17605, fabG] |
| 78 | GO:0006631 | fatty acid metabolic process | [Group11] | 4 | 18 | [FD63_RS05600, FD63_RS09110, FD63_RS17605, fabG] |
| 79 | GO:0006629 | lipid metabolic process | [Group11] | 7 | 18 | [FD63_RS01075, FD63_RS05600, FD63_RS07060, FD63_RS09110, FD63_RS13185, FD63_RS17605, fabG] |
| 80 | GO:0032787 | monocarboxylic acid metabolic process | [Group11] | 4 | 18 | [FD63_RS05600, FD63_RS09110, FD63_RS17605, fabG] |
| 81 | GO:0044283 | small molecule biosynthetic process | [Group11] | 6 | 17 | [FD63_RS06075, FD63_RS08235, FD63_RS10450, FD63_RS14240, FD63_RS17605, fabG] |
| 82 | GO:0006520 | cellular amino acid metabolic process | [Group11] | 4 | 15 | [FD63_RS05760, FD63_RS06075, FD63_RS12505, FD63_RS14240] |
| 83 | GO:1901605 | alpha-amino acid metabolic process | [Group11] | 3 | 13 | [FD63_RS06075, FD63_RS12505, FD63_RS14240] |
| 84 | GO:0008610 | lipid biosynthetic process | [Group11] | 3 | 12 | [FD63_RS01075, FD63_RS17605, fabG] |
| 85 | GO:1901566 | organonitrogen compound biosynthetic process | [Group11] | 7 | 12 | [FD63_RS01365, FD63_RS04480, FD63_RS04805, FD63_RS06075, FD63_RS08235, FD63_RS10450, FD63_RS14240] |
| 86 | GO:0044272 | sulfur compound biosynthetic process | [Group11] | 3 | 12 | [FD63_RS06075, FD63_RS10450, FD63_RS14240] |
| 87 | GO:0006790 | sulfur compound metabolic process | [Group11] | 3 | 11 | [FD63_RS06075, FD63_RS10450, FD63_RS14240] |
| 88 | GO:1901576 | organic substance biosynthetic process | [Group12] | 28 | 69 | [FD63_RS01075, FD63_RS01365, FD63_RS02450, FD63_RS02640, FD63_RS04035, FD63_RS04480, FD63_RS04775, FD63_RS04805, FD63_RS05020, FD63_RS06075, FD63_RS06120, FD63_RS07860, FD63_RS07895, FD63_RS08075, FD63_RS08235, FD63_RS09220, FD63_RS10450, FD63_RS10745, FD63_RS14240, FD63_RS14945, FD63_RS14960, FD63_RS15535, FD63_RS15980, FD63_RS16230, FD63_RS17605, FD63_RS18060, FD63_RS18175, fabG] |
| 89 | GO:0044249 | cellular biosynthetic process | [Group12] | 25 | 67 | [FD63_RS01365, FD63_RS02450, FD63_RS02640, FD63_RS04035, FD63_RS04480, FD63_RS04775, FD63_RS04805, FD63_RS05020, FD63_RS06075, FD63_RS06120, FD63_RS07895, FD63_RS08075, FD63_RS08235, FD63_RS09220, FD63_RS10450, FD63_RS10745, FD63_RS14240, FD63_RS14945, FD63_RS15535, FD63_RS15980, FD63_RS16230, FD63_RS17605, FD63_RS18060, FD63_RS18175, fabG] |
| 90 | GO:1901360 | organic cyclic compound metabolic process | [Group12] | 28 | 65 | [FD63_RS01075, FD63_RS01365, FD63_RS01935, FD63_RS02450, FD63_RS02640, FD63_RS03475, FD63_RS04035, FD63_RS04775, FD63_RS05020, FD63_RS06120, FD63_RS06850, FD63_RS07895, FD63_RS08075, FD63_RS08095, FD63_RS08235, FD63_RS09220, FD63_RS10450, FD63_RS10745, FD63_RS12830, FD63_RS14425, FD63_RS14945, FD63_RS15535, FD63_RS15980, FD63_RS15995, FD63_RS16230, FD63_RS18060, FD63_RS18175, FD63_RS18585] |
| 91 | GO:0006725 | cellular aromatic compound metabolic process | [Group12] | 27 | 64 | [FD63_RS01365, FD63_RS01935, FD63_RS02450, FD63_RS02640, FD63_RS04035, FD63_RS04775, FD63_RS05020, FD63_RS06120, FD63_RS06850, FD63_RS07895, FD63_RS08075, FD63_RS08095, FD63_RS08235, FD63_RS09220, FD63_RS10450, FD63_RS10745, FD63_RS12830, FD63_RS14425, FD63_RS14945, FD63_RS15535, FD63_RS15980, FD63_RS15995, FD63_RS16230, FD63_RS16580, FD63_RS18060, FD63_RS18175, FD63_RS18585] |
| 92 | GO:0034641 | cellular nitrogen compound metabolic process | [Group12] | 27 | 64 | [FD63_RS01365, FD63_RS01935, FD63_RS02450, FD63_RS02640, FD63_RS03475, FD63_RS04035, FD63_RS04775, FD63_RS05020, FD63_RS06120, FD63_RS06850, FD63_RS07895, FD63_RS08075, FD63_RS08095, FD63_RS08235, FD63_RS09220, FD63_RS10450, FD63_RS10745, FD63_RS12830, FD63_RS14425, FD63_RS14945, FD63_RS15535, FD63_RS15980, FD63_RS15995, FD63_RS16230, FD63_RS18060, FD63_RS18175, FD63_RS18585] |
| 93 | GO:0046483 | heterocycle metabolic process | [Group12] | 27 | 64 | [FD63_RS01365, FD63_RS01935, FD63_RS02450, FD63_RS02640, FD63_RS03475, FD63_RS04035, FD63_RS04775, FD63_RS05020, FD63_RS06120, FD63_RS06850, FD63_RS07895, FD63_RS08075, FD63_RS08095, FD63_RS08235, FD63_RS09220, FD63_RS10450, FD63_RS10745, FD63_RS12830, FD63_RS14425, FD63_RS14945, FD63_RS15535, FD63_RS15980, FD63_RS15995, FD63_RS16230, FD63_RS18060, FD63_RS18175, FD63_RS18585] |
| 94 | GO:0034654 | nucleobase-containing compound biosynthetic process | [Group12] | 15 | 60 | [FD63_RS02450, FD63_RS02640, FD63_RS04035, FD63_RS04775, FD63_RS06120, FD63_RS07895, FD63_RS08075, FD63_RS09220, FD63_RS10745, FD63_RS14945, FD63_RS15535, FD63_RS15980, FD63_RS16230, FD63_RS18060, FD63_RS18175] |
| 95 | GO:1901362 | organic cyclic compound biosynthetic process | [Group12] | 19 | 58 | [FD63_RS01075, FD63_RS01365, FD63_RS02450, FD63_RS02640, FD63_RS04035, FD63_RS04775, FD63_RS06120, FD63_RS07895, FD63_RS08075, FD63_RS08235, FD63_RS09220, FD63_RS10450, FD63_RS10745, FD63_RS14945, FD63_RS15535, FD63_RS15980, FD63_RS16230, FD63_RS18060, FD63_RS18175] |
| 96 | GO:0050794 | regulation of cellular process | [Group12] | 25 | 58 | [FD63_RS02225, FD63_RS02450, FD63_RS02640, FD63_RS03435, FD63_RS04035, FD63_RS04505, FD63_RS04775, FD63_RS04805, FD63_RS04845, FD63_RS06100, FD63_RS07895, FD63_RS08075, FD63_RS09220, FD63_RS10150, FD63_RS10745, FD63_RS11430, FD63_RS11525, FD63_RS14945, FD63_RS15155, FD63_RS15160, FD63_RS15535, FD63_RS16230, FD63_RS17215, FD63_RS18050, FD63_RS18175] |
| 97 | GO:0019438 | aromatic compound biosynthetic process | [Group12] | 18 | 57 | [FD63_RS01365, FD63_RS02450, FD63_RS02640, FD63_RS04035, FD63_RS04775, FD63_RS06120, FD63_RS07895, FD63_RS08075, FD63_RS08235, FD63_RS09220, FD63_RS10450, FD63_RS10745, FD63_RS14945, FD63_RS15535, FD63_RS15980, FD63_RS16230, FD63_RS18060, FD63_RS18175] |
| 98 | GO:0044271 | cellular nitrogen compound biosynthetic process | [Group12] | 18 | 57 | [FD63_RS01365, FD63_RS02450, FD63_RS02640, FD63_RS04035, FD63_RS04775, FD63_RS06120, FD63_RS07895, FD63_RS08075, FD63_RS08235, FD63_RS09220, FD63_RS10450, FD63_RS10745, FD63_RS14945, FD63_RS15535, FD63_RS15980, FD63_RS16230, FD63_RS18060, FD63_RS18175] |
| 99 | GO:0018130 | heterocycle biosynthetic process | [Group12] | 18 | 57 | [FD63_RS01365, FD63_RS02450, FD63_RS02640, FD63_RS04035, FD63_RS04775, FD63_RS06120, FD63_RS07895, FD63_RS08075, FD63_RS08235, FD63_RS09220, FD63_RS10450, FD63_RS10745, FD63_RS14945, FD63_RS15535, FD63_RS15980, FD63_RS16230, FD63_RS18060, FD63_RS18175] |
| 100 | GO:0031326 | regulation of cellular biosynthetic process | [Group12] | 12 | 56 | [FD63_RS02450, FD63_RS02640, FD63_RS04035, FD63_RS04775, FD63_RS07895, FD63_RS08075, FD63_RS09220, FD63_RS10745, FD63_RS14945, FD63_RS15535, FD63_RS16230, FD63_RS18175] |
| 101 | GO:0010556 | regulation of macromolecule biosynthetic process | [Group12] | 12 | 56 | [FD63_RS02450, FD63_RS02640, FD63_RS04035, FD63_RS04775, FD63_RS07895, FD63_RS08075, FD63_RS09220, FD63_RS10745, FD63_RS14945, FD63_RS15535, FD63_RS16230, FD63_RS18175] |
| 102 | GO:0060255 | regulation of macromolecule metabolic process | [Group12] | 12 | 56 | [FD63_RS02450, FD63_RS02640, FD63_RS04035, FD63_RS04775, FD63_RS07895, FD63_RS08075, FD63_RS09220, FD63_RS10745, FD63_RS14945, FD63_RS15535, FD63_RS16230, FD63_RS18175] |
| 103 | GO:0019222 | regulation of metabolic process | [Group12] | 12 | 56 | [FD63_RS02450, FD63_RS02640, FD63_RS04035, FD63_RS04775, FD63_RS07895, FD63_RS08075, FD63_RS09220, FD63_RS10745, FD63_RS14945, FD63_RS15535, FD63_RS16230, FD63_RS18175] |
| 104 | GO:0019219 | regulation of nucleobase-containing compound metabolic process | [Group12] | 12 | 56 | [FD63_RS02450, FD63_RS02640, FD63_RS04035, FD63_RS04775, FD63_RS07895, FD63_RS08075, FD63_RS09220, FD63_RS10745, FD63_RS14945, FD63_RS15535, FD63_RS16230, FD63_RS18175] |
| 105 | GO:2001141 | regulation of RNA biosynthetic process | [Group12] | 12 | 56 | [FD63_RS02450, FD63_RS02640, FD63_RS04035, FD63_RS04775, FD63_RS07895, FD63_RS08075, FD63_RS09220, FD63_RS10745, FD63_RS14945, FD63_RS15535, FD63_RS16230, FD63_RS18175] |
| 106 | GO:0032774 | RNA biosynthetic process | [Group12] | 12 | 56 | [FD63_RS02450, FD63_RS02640, FD63_RS04035, FD63_RS04775, FD63_RS07895, FD63_RS08075, FD63_RS09220, FD63_RS10745, FD63_RS14945, FD63_RS15535, FD63_RS16230, FD63_RS18175] |
| 107 | GO:2000112 | regulation of cellular macromolecule biosynthetic process | [Group12] | 12 | 55 | [FD63_RS02450, FD63_RS02640, FD63_RS04035, FD63_RS04775, FD63_RS07895, FD63_RS08075, FD63_RS09220, FD63_RS10745, FD63_RS14945, FD63_RS15535, FD63_RS16230, FD63_RS18175] |
| 108 | GO:0031323 | regulation of cellular metabolic process | [Group12] | 12 | 55 | [FD63_RS02450, FD63_RS02640, FD63_RS04035, FD63_RS04775, FD63_RS07895, FD63_RS08075, FD63_RS09220, FD63_RS10745, FD63_RS14945, FD63_RS15535, FD63_RS16230, FD63_RS18175] |
| 109 | GO:0051252 | regulation of RNA metabolic process | [Group12] | 12 | 55 | [FD63_RS02450, FD63_RS02640, FD63_RS04035, FD63_RS04775, FD63_RS07895, FD63_RS08075, FD63_RS09220, FD63_RS10745, FD63_RS14945, FD63_RS15535, FD63_RS16230, FD63_RS18175] |
| 110 | GO:0006355 | regulation of transcription, DNA-templated | [Group12] | 12 | 55 | [FD63_RS02450, FD63_RS02640, FD63_RS04035, FD63_RS04775, FD63_RS07895, FD63_RS08075, FD63_RS09220, FD63_RS10745, FD63_RS14945, FD63_RS15535, FD63_RS16230, FD63_RS18175] |
| 111 | GO:0016070 | RNA metabolic process | [Group12] | 12 | 55 | [FD63_RS02450, FD63_RS02640, FD63_RS04035, FD63_RS04775, FD63_RS07895, FD63_RS08075, FD63_RS09220, FD63_RS10745, FD63_RS14945, FD63_RS15535, FD63_RS16230, FD63_RS18175] |
| 112 | GO:0006351 | transcription, DNA-templated | [Group12] | 12 | 55 | [FD63_RS02450, FD63_RS02640, FD63_RS04035, FD63_RS04775, FD63_RS07895, FD63_RS08075, FD63_RS09220, FD63_RS10745, FD63_RS14945, FD63_RS15535, FD63_RS16230, FD63_RS18175] |
| 113 | GO:0010467 | gene expression | [Group12] | 12 | 54 | [FD63_RS02450, FD63_RS02640, FD63_RS04035, FD63_RS04775, FD63_RS07895, FD63_RS08075, FD63_RS09220, FD63_RS10745, FD63_RS14945, FD63_RS15535, FD63_RS16230, FD63_RS18175] |
| 114 | GO:0097659 | nucleic acid-templated transcription | [Group12] | 12 | 54 | [FD63_RS02450, FD63_RS02640, FD63_RS04035, FD63_RS04775, FD63_RS07895, FD63_RS08075, FD63_RS09220, FD63_RS10745, FD63_RS14945, FD63_RS15535, FD63_RS16230, FD63_RS18175] |
| 115 | GO:0009889 | regulation of biosynthetic process | [Group12] | 12 | 54 | [FD63_RS02450, FD63_RS02640, FD63_RS04035, FD63_RS04775, FD63_RS07895, FD63_RS08075, FD63_RS09220, FD63_RS10745, FD63_RS14945, FD63_RS15535, FD63_RS16230, FD63_RS18175] |
| 116 | GO:0010468 | regulation of gene expression | [Group12] | 12 | 54 | [FD63_RS02450, FD63_RS02640, FD63_RS04035, FD63_RS04775, FD63_RS07895, FD63_RS08075, FD63_RS09220, FD63_RS10745, FD63_RS14945, FD63_RS15535, FD63_RS16230, FD63_RS18175] |
| 117 | GO:1903506 | regulation of nucleic acid-templated transcription | [Group12] | 12 | 54 | [FD63_RS02450, FD63_RS02640, FD63_RS04035, FD63_RS04775, FD63_RS07895, FD63_RS08075, FD63_RS09220, FD63_RS10745, FD63_RS14945, FD63_RS15535, FD63_RS16230, FD63_RS18175] |
| 118 | GO:0051171 | regulation of nitrogen compound metabolic process | [Group12] | 12 | 53 | [FD63_RS02450, FD63_RS02640, FD63_RS04035, FD63_RS04775, FD63_RS07895, FD63_RS08075, FD63_RS09220, FD63_RS10745, FD63_RS14945, FD63_RS15535, FD63_RS16230, FD63_RS18175] |
| 119 | GO:0080090 | regulation of primary metabolic process | [Group12] | 12 | 53 | [FD63_RS02450, FD63_RS02640, FD63_RS04035, FD63_RS04775, FD63_RS07895, FD63_RS08075, FD63_RS09220, FD63_RS10745, FD63_RS14945, FD63_RS15535, FD63_RS16230, FD63_RS18175] |
| 120 | GO:0035556 | intracellular signal transduction | [Group12] | 13 | 39 | [FD63_RS02640, FD63_RS04035, FD63_RS04505, FD63_RS04775, FD63_RS04845, FD63_RS10150, FD63_RS10745, FD63_RS11430, FD63_RS11525, FD63_RS14945, FD63_RS15535, FD63_RS17215, FD63_RS18175] |
| 121 | GO:0007165 | signal transduction | [Group12] | 13 | 39 | [FD63_RS02640, FD63_RS04035, FD63_RS04505, FD63_RS04775, FD63_RS04845, FD63_RS10150, FD63_RS10745, FD63_RS11430, FD63_RS11525, FD63_RS14945, FD63_RS15535, FD63_RS17215, FD63_RS18175] |
| 122 | GO:0000160 | phosphorelay signal transduction system | [Group12] | 13 | 38 | [FD63_RS02640, FD63_RS04035, FD63_RS04505, FD63_RS04775, FD63_RS04845, FD63_RS10150, FD63_RS10745, FD63_RS11430, FD63_RS11525, FD63_RS14945, FD63_RS15535, FD63_RS17215, FD63_RS18175] |

**Supplementary material, table S5: GO Molecular function terms of putative Zn-binding proteins**

| S. No. | GO Molecular function ID | GO Molecular function Term | GO Molecular function Groups (Based on kappa score) | Neighboring Genes | Degree/ Links of the GO term | Associated Genes Found in particular GO term |
| --- | --- | --- | --- | --- | --- | --- |
| 1 | GO:0000287 | magnesium ion binding | [Group00] | 3 | 4 | [FD63_RS04055, FD63_RS13185, FD63_RS15970] |
| 2 | GO:0005507 | copper ion binding | [Group01] | 3 | 4 | [FD63_RS01935, FD63_RS14675, FD63_RS16240] |
| 3 | GO:0004386 | helicase activity | [Group02] | 3 | 4 | [FD63_RS05020, FD63_RS05700, FD63_RS16150] |
| 4 | GO:0016746 | transferase activity, transferring acyl groups | [Group03] | 3 | 3 | [FD63_RS09670, FD63_RS18350, fabG] |
| 5 | GO:0016788 | hydrolase activity, acting on ester bonds | [Group04] | 4 | 4 | [FD63_RS07060, FD63_RS08095, FD63_RS14425, FD63_RS15085] |
| 6 | GO:0016810 | hydrolase activity, acting on carbon-nitrogen (but not peptide) bonds | [Group05] | 5 | 5 | [FD63_RS03475, FD63_RS07095, FD63_RS07135, FD63_RS15020, FD63_RS15190] |
| 7 | GO:0016835 | carbon-oxygen lyase activity | [Group06] | 5 | 7 | [FD63_RS01365, FD63_RS07635, FD63_RS11105, FD63_RS11600, FD63_RS15995] |
| 8 | GO:0016836 | hydro-lyase activity | [Group06] | 5 | 7 | [FD63_RS01365, FD63_RS07635, FD63_RS11105, FD63_RS11600, FD63_RS15995] |
| 9 | GO:0016859 | cis-trans isomerase activity | [Group07] | 4 | 6 | [FD63_RS03805, FD63_RS05925, FD63_RS08365, FD63_RS11575] |
| 10 | GO:0003755 | peptidyl-prolyl cis-trans isomerase activity | [Group07] | 4 | 6 | [FD63_RS03805, FD63_RS05925, FD63_RS08365, FD63_RS11575] |
| 11 | GO:0003676 | nucleic acid binding | [Group08] | 19 | 21 | [FD63_RS02190, FD63_RS02450, FD63_RS02640, FD63_RS04035, FD63_RS04775, FD63_RS05020, FD63_RS05700, FD63_RS06120, FD63_RS07895, FD63_RS08075, FD63_RS09220, FD63_RS10745, FD63_RS14425, FD63_RS14945, FD63_RS15535, FD63_RS16150, FD63_RS16230, FD63_RS18175, FD63_RS18585] |
| 12 | GO:0003677 | DNA binding | [Group08] | 14 | 16 | [FD63_RS02190, FD63_RS02450, FD63_RS02640, FD63_RS04035, FD63_RS04775, FD63_RS06120, FD63_RS07895, FD63_RS08075, FD63_RS09220, FD63_RS10745, FD63_RS14945, FD63_RS15535, FD63_RS16230, FD63_RS18175] |
| 13 | GO:0016614 | oxidoreductase activity, acting on CH-OH group of donors | [Group09] | 10 | 12 | [FD63_RS00320, FD63_RS01075, FD63_RS04055, FD63_RS05600, FD63_RS07860, FD63_RS09110, FD63_RS14960, FD63_RS15095, FD63_RS15980, fabG] |
| 14 | GO:0016616 | oxidoreductase activity, acting on the CH-OH group of donors, NAD or NADP as acceptor | [Group09] | 10 | 12 | [FD63_RS00320, FD63_RS01075, FD63_RS04055, FD63_RS05600, FD63_RS07860, FD63_RS09110, FD63_RS14960, FD63_RS15095, FD63_RS15980, fabG] |
| 15 | GO:0016798 | hydrolase activity, acting on glycosyl bonds | [Group10] | 5 | 7 | [FD63_RS02025, FD63_RS06850, FD63_RS12830, FD63_RS13230, FD63_RS18685] |
| 16 | GO:0004553 | hydrolase activity, hydrolyzing O-glycosyl compounds | [Group10] | 3 | 5 | [FD63_RS02025, FD63_RS13230, FD63_RS18685] |
| 17 | GO:0046914 | transition metal ion binding | [Group11, Group14] | 27 | 35 | [FD63_RS00125, FD63_RS01860, FD63_RS01935, FD63_RS02920, FD63_RS03880, FD63_RS04485, FD63_RS04540, FD63_RS04645, FD63_RS05025, FD63_RS05235, FD63_RS06075, FD63_RS09220, FD63_RS11105, FD63_RS11600, FD63_RS11755, FD63_RS12120, FD63_RS13105, FD63_RS13110, FD63_RS14595, FD63_RS14645, FD63_RS14675, FD63_RS15095, FD63_RS15150, FD63_RS16240, FD63_RS16580, FD63_RS17225, hflB] |
| 18 | GO:0005506 | iron ion binding | [Group11, Group14] | 11 | 18 | [FD63_RS01935, FD63_RS04485, FD63_RS05025, FD63_RS05235, FD63_RS11755, FD63_RS13105, FD63_RS13110, FD63_RS14645, FD63_RS15150, FD63_RS16580, FD63_RS17225] |
| 19 | GO:0046872 | metal ion binding | [Group11] | 43 | 48 | [FD63_RS00125, FD63_RS00945, FD63_RS00950, FD63_RS01025, FD63_RS01125, FD63_RS01365, FD63_RS01860, FD63_RS01935, FD63_RS02920, FD63_RS03880, FD63_RS04055, FD63_RS04485, FD63_RS04540, FD63_RS04620, FD63_RS04645, FD63_RS05025, FD63_RS05235, FD63_RS06075, FD63_RS07060, FD63_RS07135, FD63_RS09220, FD63_RS09690, FD63_RS11105, FD63_RS11295, FD63_RS11600, FD63_RS11755, FD63_RS12120, FD63_RS13105, FD63_RS13110, FD63_RS13185, FD63_RS14595, FD63_RS14645, FD63_RS14675, FD63_RS15095, FD63_RS15150, FD63_RS15970, FD63_RS16210, FD63_RS16240, FD63_RS16580, FD63_RS16765, FD63_RS17225, FD63_RS18060, hflB] |
| 20 | GO:0043169 | cation binding | [Group11] | 43 | 46 | [FD63_RS00125, FD63_RS00945, FD63_RS00950, FD63_RS01025, FD63_RS01125, FD63_RS01365, FD63_RS01860, FD63_RS01935, FD63_RS02920, FD63_RS03880, FD63_RS04055, FD63_RS04485, FD63_RS04540, FD63_RS04620, FD63_RS04645, FD63_RS05025, FD63_RS05235, FD63_RS06075, FD63_RS07060, FD63_RS07135, FD63_RS09220, FD63_RS09690, FD63_RS11105, FD63_RS11295, FD63_RS11600, FD63_RS11755, FD63_RS12120, FD63_RS13105, FD63_RS13110, FD63_RS13185, FD63_RS14595, FD63_RS14645, FD63_RS14675, FD63_RS15095, FD63_RS15150, FD63_RS15970, FD63_RS16210, FD63_RS16240, FD63_RS16580, FD63_RS16765, FD63_RS17225, FD63_RS18060, hflB] |
| 21 | GO:0008270 | zinc ion binding | [Group11] | 13 | 15 | [FD63_RS00125, FD63_RS01860, FD63_RS02920, FD63_RS03880, FD63_RS04540, FD63_RS04645, FD63_RS06075, FD63_RS11105, FD63_RS11600, FD63_RS12120, FD63_RS15095, FD63_RS16580, hflB] |
| 22 | GO:0030170 | pyridoxal phosphate binding | [Group12] | 5 | 11 | [FD63_RS02845, FD63_RS08630, FD63_RS09400, FD63_RS09460, FD63_RS15000] |
| 23 | GO:0070279 | vitamin B6 binding | [Group12] | 5 | 11 | [FD63_RS02845, FD63_RS08630, FD63_RS09400, FD63_RS09460, FD63_RS15000] |
| 24 | GO:0019842 | vitamin binding | [Group12] | 5 | 10 | [FD63_RS02845, FD63_RS08630, FD63_RS09400, FD63_RS09460, FD63_RS15000] |
| 25 | GO:0008483 | transaminase activity | [Group12] | 3 | 8 | [FD63_RS02845, FD63_RS08630, FD63_RS15000] |
| 26 | GO:0016769 | transferase activity, transferring nitrogenous groups | [Group12] | 3 | 8 | [FD63_RS02845, FD63_RS08630, FD63_RS15000] |
| 27 | GO:0050662 | coenzyme binding | [Group13] | 7 | 11 | [FD63_RS00320, FD63_RS02535, FD63_RS03435, FD63_RS04055, FD63_RS14240, FD63_RS14410, fabG] |
| 28 | GO:0016667 | oxidoreductase activity, acting on a sulfur group of donors | [Group13] | 5 | 9 | [FD63_RS02225, FD63_RS03435, FD63_RS06100, FD63_RS14240, FD63_RS15160] |
| 29 | GO:0015036 | disulfide oxidoreductase activity | [Group13] | 4 | 8 | [FD63_RS02225, FD63_RS03435, FD63_RS06100, FD63_RS15160] |
| 30 | GO:0050660 | flavin adenine dinucleotide binding | [Group13] | 3 | 8 | [FD63_RS02535, FD63_RS03435, FD63_RS14240] |
| 31 | GO:0051287 | NAD binding | [Group13] | 5 | 8 | [FD63_RS00320, FD63_RS03435, FD63_RS04055, FD63_RS14410, fabG] |
| 32 | GO:0015035 | protein disulfide oxidoreductase activity | [Group13] | 4 | 7 | [FD63_RS02225, FD63_RS03435, FD63_RS06100, FD63_RS15160] |
| 33 | GO:0009055 | electron transfer activity | [Group14, Group16] | 11 | 24 | [FD63_RS01935, FD63_RS01945, FD63_RS03435, FD63_RS04620, FD63_RS05235, FD63_RS05240, FD63_RS09690, FD63_RS11295, FD63_RS14645, FD63_RS14675, FD63_RS15150] |
| 34 | GO:0020037 | heme binding | [Group14, Group16] | 8 | 12 | [FD63_RS01935, FD63_RS04620, FD63_RS05235, FD63_RS09690, FD63_RS11295, FD63_RS13105, FD63_RS13110, FD63_RS15150] |
| 35 | GO:0046906 | tetrapyrrole binding | [Group14, Group16] | 8 | 12 | [FD63_RS01935, FD63_RS04620, FD63_RS05235, FD63_RS09690, FD63_RS11295, FD63_RS13105, FD63_RS13110, FD63_RS15150] |
| 36 | GO:0008199 | ferric iron binding | [Group14] | 4 | 7 | [FD63_RS04485, FD63_RS05025, FD63_RS11755, FD63_RS17225] |
| 37 | GO:0016722 | oxidoreductase activity, oxidizing metal ions | [Group14] | 3 | 5 | [FD63_RS05025, FD63_RS11755, FD63_RS17225] |
| 38 | GO:0070011 | peptidase activity, acting on L-amino acid peptides | [Group15] | 15 | 27 | [FD63_RS00430, FD63_RS01125, FD63_RS01330, FD63_RS01865, FD63_RS02920, FD63_RS04540, FD63_RS06445, FD63_RS06450, FD63_RS06915, FD63_RS12400, FD63_RS14595, FD63_RS16210, FD63_RS17735, FD63_RS18300, hflB] |
| 39 | GO:0008233 | peptidase activity | [Group15] | 16 | 24 | [FD63_RS00430, FD63_RS01125, FD63_RS01330, FD63_RS01865, FD63_RS02920, FD63_RS04540, FD63_RS04805, FD63_RS06445, FD63_RS06450, FD63_RS06915, FD63_RS12400, FD63_RS14595, FD63_RS16210, FD63_RS17735, FD63_RS18300, hflB] |
| 40 | GO:0008237 | metallopeptidase activity | [Group15] | 7 | 14 | [FD63_RS01125, FD63_RS02920, FD63_RS04540, FD63_RS06445, FD63_RS06450, FD63_RS16210, hflB] |
| 41 | GO:0008238 | exopeptidase activity | [Group15] | 6 | 12 | [FD63_RS00430, FD63_RS01125, FD63_RS01865, FD63_RS02920, FD63_RS04540, FD63_RS14595] |
| 42 | GO:0004175 | endopeptidase activity | [Group15] | 5 | 11 | [FD63_RS01125, FD63_RS06445, FD63_RS06450, FD63_RS16210, hflB] |
| 43 | GO:0004222 | metalloendopeptidase activity | [Group15] | 5 | 11 | [FD63_RS01125, FD63_RS06445, FD63_RS06450, FD63_RS16210, hflB] |
| 44 | GO:0008236 | serine-type peptidase activity | [Group15] | 5 | 10 | [FD63_RS01330, FD63_RS06915, FD63_RS12400, FD63_RS17735, FD63_RS18300] |
| 45 | GO:0017171 | serine hydrolase activity | [Group15] | 5 | 9 | [FD63_RS01330, FD63_RS06915, FD63_RS12400, FD63_RS17735, FD63_RS18300] |
| 46 | GO:0004177 | aminopeptidase activity | [Group15] | 4 | 6 | [FD63_RS01865, FD63_RS02920, FD63_RS04540, FD63_RS14595] |
| 47 | GO:0004129 | cytochrome-c oxidase activity | [Group16] | 4 | 18 | [FD63_RS01935, FD63_RS01945, FD63_RS05235, FD63_RS05240] |
| 48 | GO:0008324 | cation transmembrane transporter activity | [Group16] | 4 | 16 | [FD63_RS01935, FD63_RS01945, FD63_RS05235, FD63_RS05240] |
| 49 | GO:0015078 | hydrogen ion transmembrane transporter activity | [Group16] | 4 | 16 | [FD63_RS01935, FD63_RS01945, FD63_RS05235, FD63_RS05240] |
| 50 | GO:0022890 | inorganic cation transmembrane transporter activity | [Group16] | 4 | 16 | [FD63_RS01935, FD63_RS01945, FD63_RS05235, FD63_RS05240] |
| 51 | GO:0015075 | ion transmembrane transporter activity | [Group16] | 4 | 16 | [FD63_RS01935, FD63_RS01945, FD63_RS05235, FD63_RS05240] |
| 52 | GO:0015077 | monovalent inorganic cation transmembrane transporter activity | [Group16] | 4 | 16 | [FD63_RS01935, FD63_RS01945, FD63_RS05235, FD63_RS05240] |
| 53 | GO:0016676 | oxidoreductase activity, acting on a heme group of donors, oxygen as acceptor | [Group16] | 4 | 16 | [FD63_RS01935, FD63_RS01945, FD63_RS05235, FD63_RS05240] |
| 54 | GO:0015002 | heme-copper terminal oxidase activity | [Group16] | 4 | 15 | [FD63_RS01935, FD63_RS01945, FD63_RS05235, FD63_RS05240] |
| 55 | GO:0016675 | oxidoreductase activity, acting on a heme group of donors | [Group16] | 4 | 15 | [FD63_RS01935, FD63_RS01945, FD63_RS05235, FD63_RS05240] |
| 56 | GO:0022891 | substrate-specific transmembrane transporter activity | [Group16] | 4 | 15 | [FD63_RS01935, FD63_RS01945, FD63_RS05235, FD63_RS05240] |
| 57 | GO:0043168 | anion binding | [Group17] | 29 | 46 | [FD63_RS00510, FD63_RS02190, FD63_RS02535, FD63_RS02640, FD63_RS02845, FD63_RS03435, FD63_RS03800, FD63_RS04480, FD63_RS04620, FD63_RS04755, FD63_RS04805, FD63_RS05020, FD63_RS05700, FD63_RS05740, FD63_RS08235, FD63_RS08250, FD63_RS08630, FD63_RS09400, FD63_RS09460, FD63_RS10450, FD63_RS10770, FD63_RS12840, FD63_RS13965, FD63_RS14240, FD63_RS15000, FD63_RS16135, FD63_RS16150, FD63_RS18915, hflB] |
| 58 | GO:0000166 | nucleotide binding | [Group17] | 26 | 45 | [FD63_RS00320, FD63_RS00510, FD63_RS02190, FD63_RS02535, FD63_RS02640, FD63_RS03435, FD63_RS03800, FD63_RS04055, FD63_RS04620, FD63_RS04755, FD63_RS05020, FD63_RS05700, FD63_RS05740, FD63_RS08235, FD63_RS08250, FD63_RS10450, FD63_RS10770, FD63_RS12840, FD63_RS13965, FD63_RS14240, FD63_RS14410, FD63_RS16135, FD63_RS16150, FD63_RS18915, fabG, hflB] |
| 59 | GO:1901265 | nucleoside phosphate binding | [Group17] | 26 | 42 | [FD63_RS00320, FD63_RS00510, FD63_RS02190, FD63_RS02535, FD63_RS02640, FD63_RS03435, FD63_RS03800, FD63_RS04055, FD63_RS04620, FD63_RS04755, FD63_RS05020, FD63_RS05700, FD63_RS05740, FD63_RS08235, FD63_RS08250, FD63_RS10450, FD63_RS10770, FD63_RS12840, FD63_RS13965, FD63_RS14240, FD63_RS14410, FD63_RS16135, FD63_RS16150, FD63_RS18915, fabG, hflB] |
| 60 | GO:0017076 | purine nucleotide binding | [Group17] | 19 | 36 | [FD63_RS00510, FD63_RS02190, FD63_RS02640, FD63_RS03800, FD63_RS04620, FD63_RS04755, FD63_RS05020, FD63_RS05700, FD63_RS05740, FD63_RS08235, FD63_RS08250, FD63_RS10450, FD63_RS10770, FD63_RS12840, FD63_RS13965, FD63_RS16135, FD63_RS16150, FD63_RS18915, hflB] |
| 61 | GO:0035639 | purine ribonucleoside triphosphate binding | [Group17] | 19 | 36 | [FD63_RS00510, FD63_RS02190, FD63_RS02640, FD63_RS03800, FD63_RS04620, FD63_RS04755, FD63_RS05020, FD63_RS05700, FD63_RS05740, FD63_RS08235, FD63_RS08250, FD63_RS10450, FD63_RS10770, FD63_RS12840, FD63_RS13965, FD63_RS16135, FD63_RS16150, FD63_RS18915, hflB] |
| 62 | GO:0032555 | purine ribonucleotide binding | [Group17] | 19 | 36 | [FD63_RS00510, FD63_RS02190, FD63_RS02640, FD63_RS03800, FD63_RS04620, FD63_RS04755, FD63_RS05020, FD63_RS05700, FD63_RS05740, FD63_RS08235, FD63_RS08250, FD63_RS10450, FD63_RS10770, FD63_RS12840, FD63_RS13965, FD63_RS16135, FD63_RS16150, FD63_RS18915, hflB] |
| 63 | GO:0032553 | ribonucleotide binding | [Group17] | 20 | 36 | [FD63_RS00510, FD63_RS02190, FD63_RS02640, FD63_RS03800, FD63_RS04620, FD63_RS04755, FD63_RS05020, FD63_RS05700, FD63_RS05740, FD63_RS08235, FD63_RS08250, FD63_RS10450, FD63_RS10770, FD63_RS12840, FD63_RS13965, FD63_RS14240, FD63_RS16135, FD63_RS16150, FD63_RS18915, hflB] |
| 64 | GO:0032559 | adenyl ribonucleotide binding | [Group17] | 18 | 35 | [FD63_RS00510, FD63_RS02190, FD63_RS02640, FD63_RS04620, FD63_RS04755, FD63_RS05020, FD63_RS05700, FD63_RS05740, FD63_RS08235, FD63_RS08250, FD63_RS10450, FD63_RS10770, FD63_RS12840, FD63_RS13965, FD63_RS16135, FD63_RS16150, FD63_RS18915, hflB] |
| 65 | GO:0030554 | adenyl nucleotide binding | [Group17] | 18 | 34 | [FD63_RS00510, FD63_RS02190, FD63_RS02640, FD63_RS04620, FD63_RS04755, FD63_RS05020, FD63_RS05700, FD63_RS05740, FD63_RS08235, FD63_RS08250, FD63_RS10450, FD63_RS10770, FD63_RS12840, FD63_RS13965, FD63_RS16135, FD63_RS16150, FD63_RS18915, hflB] |
| 66 | GO:0005524 | ATP binding | [Group17] | 18 | 34 | [FD63_RS00510, FD63_RS02190, FD63_RS02640, FD63_RS04620, FD63_RS04755, FD63_RS05020, FD63_RS05700, FD63_RS05740, FD63_RS08235, FD63_RS08250, FD63_RS10450, FD63_RS10770, FD63_RS12840, FD63_RS13965, FD63_RS16135, FD63_RS16150, FD63_RS18915, hflB] |
| 67 | GO:0017111 | nucleoside-triphosphatase activity | [Group17] | 12 | 29 | [FD63_RS00510, FD63_RS03800, FD63_RS04620, FD63_RS04755, FD63_RS05020, FD63_RS05700, FD63_RS08250, FD63_RS10770, FD63_RS13965, FD63_RS16150, FD63_RS18915, hflB] |
| 68 | GO:0016818 | hydrolase activity, acting on acid anhydrides, in phosphorus-containing anhydrides | [Group17] | 12 | 28 | [FD63_RS00510, FD63_RS03800, FD63_RS04620, FD63_RS04755, FD63_RS05020, FD63_RS05700, FD63_RS08250, FD63_RS10770, FD63_RS13965, FD63_RS16150, FD63_RS18915, hflB] |
| 69 | GO:0016462 | pyrophosphatase activity | [Group17] | 12 | 28 | [FD63_RS00510, FD63_RS03800, FD63_RS04620, FD63_RS04755, FD63_RS05020, FD63_RS05700, FD63_RS08250, FD63_RS10770, FD63_RS13965, FD63_RS16150, FD63_RS18915, hflB] |
| 70 | GO:0016817 | hydrolase activity, acting on acid anhydrides | [Group17] | 12 | 27 | [FD63_RS00510, FD63_RS03800, FD63_RS04620, FD63_RS04755, FD63_RS05020, FD63_RS05700, FD63_RS08250, FD63_RS10770, FD63_RS13965, FD63_RS16150, FD63_RS18915, hflB] |
| 71 | GO:0016887 | ATPase activity | [Group17] | 9 | 24 | [FD63_RS00510, FD63_RS04620, FD63_RS04755, FD63_RS05020, FD63_RS08250, FD63_RS10770, FD63_RS13965, FD63_RS18915, hflB] |

**Supplementary material, table S6: Putative Zn-binding proteins of *Xtu* homologous to PHI-base**

| **S. No.** | **Sequence (Query) Id of predicted Zn-binding proteins** | **PHI (Subject) sequence Id provided by PHI database** | **Percentage og identical matches** | **Alignment length** | **No. of mismatches** | **No. of Gap openings** | **Start of alignment in query** | **End of alignment in query** | **Start of alignment in subject** | **End of alignment in subject** | **Expect value** | **Bit score** |
| --- | --- | --- | --- | --- | --- | --- | --- | --- | --- | --- | --- | --- |
| 1 | WP_003465234.1 | PHI:2440 | 43.95 | 223 | 120 | 2 | 1 | 219 | 1 | 222 | 1E-52 | 170 |
| 2 | WP_003465242.1 | PHI:5271 | 36.33 | 245 | 151 | 2 | 4 | 246 | 8 | 249 | 5E-45 | 151 |
| 3 | WP_003465369.1 | PHI:191 | 25.45 | 715 | 368 | 26 | 93 | 689 | 101 | 768 | 1E-33 | 135 |
| 4 | WP_003465445.1 | PHI:5271 | 33.74 | 243 | 153 | 7 | 3 | 240 | 6 | 245 | 6.00E-25 | 97.8 |
| 5 | WP_003465522.1 | PHI:4477 | 57.09 | 254 | 109 | 0 | 14 | 267 | 6 | 259 | 5E-103 | 302 |
| 6 | WP_003465615.1 | PHI:3842 | 25.66 | 226 | 148 | 9 | 1 | 220 | 143 | 354 | 0.0001 | 40 |
| 7 | WP_003465620.1 | PHI:5066 | 35.15 | 202 | 117 | 2 | 2 | 198 | 1 | 193 | 2.00E-29 | 108 |
| 8 | WP_003465625.1 | PHI:2796 | 32.04 | 284 | 181 | 7 | 144 | 427 | 37 | 308 | 4E-33 | 134 |
| 9 | WP_003466157.1 | PHI:2445 | 35.11 | 225 | 140 | 4 | 12 | 234 | 21 | 241 | 1E-26 | 102 |
| 10 | WP_003466231.1 | PHI:2920 | 30.96 | 281 | 146 | 7 | 58 | 334 | 26 | 262 | 1E-29 | 120 |
| 11 | WP_003466285.1 | PHI:3412 | 28.57 | 210 | 136 | 6 | 24 | 223 | 36 | 241 | 1E-20 | 85.9 |
| 12 | WP_003466345.1 | PHI:3412 | 30.47 | 233 | 140 | 6 | 11 | 228 | 30 | 255 | 6E-22 | 89.4 |
| 13 | WP_003466735.1 | PHI:3843 | 28.8 | 184 | 101 | 8 | 120 | 295 | 248 | 409 | 4E-08 | 51.2 |
| 14 | WP_003466789.1 | PHI:1133 | 26.39 | 269 | 154 | 12 | 5 | 235 | 7 | 269 | 0.00002 | 42.7 |
| 15 | WP_003466959.1 | PHI:5271 | 35.48 | 248 | 153 | 4 | 4 | 250 | 5 | 246 | 2E-35 | 127 |
| 16 | WP_003467341.1 | PHI:59 | 25 | 192 | 135 | 3 | 2 | 193 | 26 | 208 | 3E-14 | 67.8 |
| 17 | WP_003467386.1 | PHI:1579 | 41.38 | 87 | 43 | 3 | 624 | 702 | 226 | 312 | 2E-09 | 57.4 |
| 18 | WP_003467543.1 | PHI:5062 | 31.45 | 124 | 84 | 1 | 4 | 127 | 11 | 133 | 3E-14 | 63.9 |
| 19 | WP_003467936.1 | PHI:1607 | 63.46 | 52 | 18 | 1 | 6 | 56 | 7 | 58 | 2E-18 | 71.2 |
| 20 | WP_003467943.1 | PHI:3071 | 28.99 | 169 | 110 | 4 | 1 | 160 | 56 | 223 | 5E-10 | 55.5 |
| 21 | WP_003468012.1 | PHI:2058 | 28.21 | 319 | 193 | 8 | 114 | 403 | 151 | 462 | 3E-27 | 114 |
| 22 | WP_003468030.1 | PHI:2042 | 33.03 | 542 | 340 | 8 | 66 | 588 | 783 | 1320 | 2E-75 | 259 |
| 23 | WP_003468480.1 | PHI:4477 | 34.69 | 98 | 64 | 0 | 103 | 200 | 120 | 217 | 1E-10 | 57 |
| 24 | WP_003468516.1 | PHI:2440 | 39.73 | 224 | 125 | 5 | 4 | 223 | 2 | 219 | 1E-42 | 144 |
| 25 | WP_003468529.1 | PHI:415 | 29.64 | 361 | 223 | 15 | 2 | 335 | 3 | 359 | 9E-32 | 120 |
| 26 | WP_003468538.1 | PHI:860 | 36.47 | 85 | 46 | 2 | 113 | 195 | 150 | 228 | 2E-06 | 46.2 |
| 27 | WP_003468654.1 | PHI:2834 | 25.42 | 118 | 87 | 1 | 140 | 257 | 6 | 122 | 0.00002 | 44.3 |
| 28 | WP_003468804.1 | PHI:5271 | 67.34 | 248 | 79 | 1 | 1 | 246 | 1 | 248 | 4E-109 | 316 |
| 29 | WP_003468806.1 | PHI:433 | 30.56 | 72 | 45 | 2 | 1 | 68 | 1781 | 1851 | 0.00004 | 37.7 |
| 30 | WP_003469157.1 | PHI:1599 | 56.8 | 125 | 51 | 2 | 2 | 123 | 53 | 177 | 1E-41 | 136 |
| 31 | WP_003469170.1 | PHI:5272 | 48.54 | 239 | 121 | 2 | 8 | 244 | 3 | 241 | 4E-64 | 200 |
| 32 | WP_003469200.1 | PHI:2440 | 30.6 | 232 | 140 | 7 | 10 | 231 | 1 | 221 | 3E-17 | 75.1 |
| 33 | WP_003469280.1 | PHI:2440 | 37.95 | 224 | 128 | 4 | 1 | 218 | 1 | 219 | 2E-42 | 143 |
| 34 | WP_003469645.1 | PHI:2789 | 59.78 | 184 | 74 | 0 | 25 | 208 | 27 | 210 | 2E-77 | 232 |
| 35 | WP_003469868.1 | PHI:2204 | 30.71 | 267 | 158 | 11 | 51 | 310 | 70 | 316 | 2E-21 | 89.7 |
| 36 | WP_003469871.1 | PHI:2209 | 32.52 | 326 | 171 | 9 | 72 | 391 | 54 | 336 | 9E-42 | 149 |
| 37 | WP_003470036.1 | PHI:881 | 36.73 | 343 | 207 | 5 | 1 | 340 | 3 | 338 | 2.00E-57 | 189 |
| 38 | WP_003470527.1 | PHI:330 | 38.03 | 142 | 76 | 4 | 68 | 201 | 13 | 150 | 2E-19 | 79.7 |
| 39 | WP_003470615.1 | PHI:277 | 50.72 | 138 | 56 | 8 | 12 | 138 | 73 | 209 | 8E-31 | 110 |
| 40 | WP_003470669.1 | PHI:1569 | 44.44 | 306 | 129 | 8 | 3 | 270 | 55 | 357 | 8E-67 | 213 |
| 41 | WP_003470782.1 | PHI:2440 | 46.67 | 225 | 117 | 1 | 1 | 222 | 1 | 225 | 3E-64 | 199 |
| 42 | WP_003470942.1 | PHI:2440 | 37.7 | 122 | 70 | 3 | 88 | 207 | 2 | 119 | 7E-16 | 74.7 |
| 43 | WP_003471228.1 | PHI:3108 | 33.91 | 115 | 70 | 3 | 144 | 256 | 4 | 114 | 1E-08 | 52 |
| 44 | WP_003471337.1 | PHI:1566 | 40.8 | 250 | 147 | 1 | 157 | 405 | 127 | 376 | 5E-59 | 202 |
| 45 | WP_003471408.1 | PHI:2305 | 31.03 | 58 | 40 | 0 | 14 | 71 | 38 | 95 | 0.00006 | 37.4 |
| 46 | WP_003471666.1 | PHI:3843 | 30.83 | 120 | 75 | 5 | 126 | 240 | 254 | 370 | 5E-07 | 47.8 |
| 47 | WP_003471682.1 | PHI:4250 | 45.66 | 357 | 185 | 4 | 12 | 368 | 9 | 356 | 9E-86 | 265 |
| 48 | WP_003471844.1 | PHI:2789 | 31.18 | 170 | 115 | 2 | 123 | 291 | 39 | 207 | 2E-16 | 72.8 |
| 49 | WP_003472018.1 | PHI:5271 | 30.59 | 255 | 152 | 7 | 8 | 253 | 10 | 248 | 1E-20 | 85.5 |
| 50 | WP_003472081.1 | PHI:2032 | 32.46 | 114 | 65 | 3 | 58 | 159 | 99 | 212 | 3E-10 | 57.4 |
| 51 | WP_003472086.1 | PHI:1607 | 61.54 | 52 | 19 | 1 | 2 | 52 | 5 | 56 | 2E-20 | 76.6 |
| 52 | WP_003472098.1 | PHI:5271 | 43.67 | 245 | 138 | 0 | 1 | 245 | 5 | 249 | 4E-61 | 193 |
| 53 | WP_003472119.1 | PHI:872 | 28.26 | 184 | 115 | 4 | 1 | 173 | 1 | 178 | 8E-07 | 48.9 |
| 54 | WP_003472142.1 | PHI:1605 | 35.39 | 503 | 237 | 15 | 19 | 459 | 51 | 527 | 2E-83 | 267 |
| 55 | WP_003472589.1 | PHI:1572 | 31.13 | 257 | 165 | 9 | 9 | 257 | 270 | 522 | 2E-20 | 92.8 |
| 56 | WP_003472647.1 | PHI:5062 | 32.76 | 116 | 77 | 1 | 4 | 119 | 11 | 125 | 3E-16 | 69.3 |
| 57 | WP_003472648.1 | PHI:5062 | 36.89 | 122 | 75 | 2 | 10 | 131 | 6 | 125 | 2E-20 | 81.6 |
| 58 | WP_003473108.1 | PHI:1772 | 30.53 | 131 | 80 | 3 | 23 | 153 | 526 | 645 | 0.00005 | 39.7 |
| 59 | WP_003473181.1 | PHI:3091 | 29.2 | 113 | 73 | 3 | 3 | 114 | 10 | 116 | 4E-07 | 43.9 |
| 60 | WP_003477578.1 | PHI:3165 | 22.67 | 225 | 158 | 7 | 50 | 260 | 64 | 286 | 5E-07 | 47 |
| 61 | WP_003481607.1 | PHI:5062 | 99.16 | 237 | 2 | 0 | 1 | 237 | 1 | 237 | 6E-170 | 469 |
| 62 | WP_003488188.1 | PHI:1607 | 61.22 | 49 | 19 | 0 | 4 | 52 | 10 | 58 | 6E-19 | 72.4 |
| 63 | WP_004425452.1 | PHI:3412 | 33.62 | 235 | 138 | 6 | 20 | 242 | 35 | 263 | 4E-32 | 119 |
| 64 | WP_004425521.1 | PHI:5271 | 34.8 | 250 | 149 | 4 | 6 | 248 | 4 | 246 | 4E-39 | 136 |
| 65 | WP_004425530.1 | PHI:541 | 26.5 | 434 | 250 | 20 | 123 | 513 | 130 | 537 | 1E-22 | 98.6 |
| 66 | WP_004425670.1 | PHI:2774 | 43.72 | 398 | 199 | 8 | 22 | 414 | 26 | 403 | 1E-85 | 289 |
| 67 | WP_004426362.1 | PHI:3234 | 26.88 | 346 | 244 | 3 | 7 | 352 | 60 | 396 | 2E-33 | 129 |
| 68 | WP_004426468.1 | PHI:2058 | 29.09 | 385 | 227 | 11 | 24 | 371 | 95 | 470 | 2E-29 | 120 |
| 69 | WP_004426584.1 | PHI:2305 | 35.09 | 114 | 59 | 4 | 210 | 310 | 2 | 113 | 8E-11 | 56.2 |
| 70 | WP_038237017.1 | PHI:5189 | 33.82 | 68 | 45 | 0 | 302 | 369 | 1039 | 1106 | 2E-07 | 49.7 |
| 71 | WP_038237239.1 | PHI:881 | 24.09 | 274 | 153 | 10 | 1 | 245 | 1 | 248 | 8E-07 | 46.6 |
| 72 | WP_038238095.1 | PHI:55 | 31.43 | 350 | 211 | 11 | 5 | 334 | 1798 | 2138 | 1E-35 | 137 |
| 73 | WP_038238334.1 | PHI:2042 | 29.44 | 248 | 156 | 8 | 17 | 249 | 371 | 614 | 1E-15 | 73.9 |
| 74 | WP_038238706.1 | PHI:330 | 33.77 | 151 | 89 | 4 | 47 | 189 | 3 | 150 | 2.00E-13 | 62 |
| 75 | WP_038238771.1 | PHI:881 | 28.07 | 228 | 148 | 6 | 1 | 221 | 3 | 221 | 2.00E-18 | 81.6 |
| 76 | WP_038239202.1 | PHI:2305 | 46.3 | 108 | 53 | 2 | 42 | 145 | 11 | 117 | 3E-27 | 97.8 |
| 77 | WP_047324417.1 | PHI:231 | 31.3 | 115 | 73 | 3 | 27 | 140 | 265 | 374 | 2E-07 | 48.9 |
| 78 | WP_047324452.1 | PHI:5271 | 34.08 | 267 | 149 | 6 | 1 | 258 | 1 | 249 | 2E-28 | 107 |
| 79 | WP_047324470.1 | PHI:881 | 28.78 | 344 | 226 | 7 | 3 | 345 | 13 | 338 | 7E-38 | 137 |
| 80 | WP_047324520.1 | PHI:3677 | 40.14 | 294 | 157 | 5 | 150 | 439 | 33 | 311 | 1E-52 | 179 |
| 81 | WP_047324567.1 | PHI:216 | 26.7 | 176 | 117 | 4 | 119 | 289 | 156 | 324 | 2E-12 | 64.3 |
| 82 | WP_047324585.1 | PHI:55 | 25.55 | 321 | 190 | 12 | 36 | 338 | 1851 | 2140 | 2E-13 | 68.2 |
| 83 | WP_047324608.1 | PHI:2796 | 34.23 | 298 | 172 | 8 | 33 | 308 | 3 | 298 | 2E-30 | 125 |
| 84 | WP_047324617.1 | PHI:3412 | 40.23 | 87 | 47 | 4 | 718 | 803 | 156 | 238 | 0.00002 | 43.9 |
| 85 | WP_047324619.1 | PHI:4616 | 28.93 | 394 | 239 | 16 | 3 | 385 | 13 | 376 | 1E-24 | 100 |
| 86 | WP_047324635.1 | PHI:3165 | 22.67 | 225 | 158 | 7 | 50 | 260 | 64 | 286 | 5E-07 | 47 |
| 87 | WP_047324671.1 | PHI:5271 | 29.67 | 246 | 170 | 2 | 19 | 264 | 4 | 246 | 6E-22 | 89.4 |
| 88 | WP_047324684.1 | PHI:4477 | 30 | 170 | 106 | 4 | 61 | 224 | 65 | 227 | 1E-11 | 62.4 |
| 89 | WP_047324755.1 | PHI:2770 | 36.05 | 233 | 141 | 3 | 7 | 237 | 4 | 230 | 2E-31 | 116 |
| 90 | WP_047324777.1 | PHI:2408 | 28.91 | 128 | 86 | 4 | 34 | 156 | 239 | 366 | 4E-08 | 50.1 |
| 91 | WP_047324797.1 | PHI:2257 | 36.36 | 286 | 148 | 5 | 3 | 256 | 51 | 334 | 3E-41 | 145 |
| 92 | WP_047324805.1 | PHI:121 | 38.91 | 239 | 98 | 6 | 167 | 375 | 3 | 223 | 2E-45 | 155 |
| 93 | WP_047324810.1 | PHI:2760 | 71.18 | 628 | 180 | 1 | 1 | 628 | 1 | 627 | 0 | 961 |
| 94 | WP_047324842.1 | PHI:5236 | 39.71 | 68 | 37 | 1 | 4 | 67 | 403 | 470 | 2.00E-07 | 49.3 |
| 95 | WP_047324950.1 | PHI:5272 | 30.8 | 250 | 146 | 7 | 16 | 249 | 3 | 241 | 2E-24 | 96.3 |
| 96 | WP_047324951.1 | PHI:685 | 26.52 | 264 | 165 | 11 | 10 | 250 | 26 | 283 | 8E-18 | 77.4 |
| 97 | WP_047324952.1 | PHI:1772 | 30.43 | 138 | 70 | 5 | 93 | 208 | 508 | 641 | 5E-09 | 52.4 |
| 98 | WP_047324961.1 | PHI:3108 | 33.88 | 121 | 78 | 2 | 5 | 125 | 1 | 119 | 2E-15 | 67.4 |
| 99 | WP_047324979.1 | PHI:1594 | 43.07 | 462 | 239 | 9 | 65 | 502 | 121 | 582 | 2E-118 | 362 |
| 100 | WP_047324986.1 | PHI:2760 | 22.08 | 154 | 110 | 3 | 548 | 693 | 51 | 202 | 0.00005 | 43.5 |
| 101 | WP_047325000.1 | PHI:2042 | 32.38 | 210 | 127 | 6 | 34 | 235 | 1094 | 1296 | 1E-18 | 82.8 |
| 102 | WP_047325048.1 | PHI:2440 | 35.16 | 91 | 57 | 1 | 27 | 117 | 25 | 113 | 2E-12 | 63.2 |
| 103 | WP_047325089.1 | PHI:881 | 32.91 | 234 | 149 | 6 | 2 | 234 | 8 | 234 | 8.00E-24 | 97.1 |
| 104 | WP_047325129.1 | PHI:1155 | 24.48 | 241 | 127 | 9 | 199 | 386 | 228 | 466 | 4E-08 | 52 |
| 105 | WP_047325244.1 | PHI:415 | 48.89 | 360 | 170 | 7 | 4 | 351 | 5 | 362 | 2E-102 | 307 |
| 106 | WP_047325261.1 | PHI:2166 | 30.28 | 109 | 65 | 3 | 73 | 172 | 152 | 258 | 7E-09 | 52.8 |
| 107 | WP_047325265.1 | PHI:4616 | 62.5 | 368 | 136 | 1 | 4 | 369 | 11 | 378 | 2E-173 | 489 |
| 108 | WP_047325420.1 | PHI:5271 | 31.89 | 254 | 157 | 5 | 1 | 246 | 1 | 246 | 8E-30 | 111 |
| 109 | WP_047325488.1 | PHI:191 | 33.74 | 246 | 120 | 9 | 691 | 894 | 551 | 795 | 6E-27 | 114 |
| 110 | WP_047325529.1 | PHI:5271 | 35.08 | 248 | 153 | 4 | 7 | 252 | 5 | 246 | 1E-34 | 124 |
| 111 | WP_047325583.1 | PHI:5271 | 31.34 | 201 | 125 | 3 | 4 | 204 | 6 | 193 | 2E-23 | 93.6 |
| 112 | WP_047325628.1 | PHI:4477 | 34.52 | 84 | 55 | 0 | 101 | 184 | 120 | 203 | 2E-10 | 56.6 |
| 113 | WP_047325709.1 | PHI:1772 | 40.3 | 201 | 99 | 2 | 2 | 181 | 441 | 641 | 2E-40 | 143 |
| 114 | WP_080964784.1 | PHI:862 | 24.91 | 558 | 367 | 18 | 239 | 760 | 231 | 772 | 3E-27 | 115 |
| 115 | WP_080964854.1 | PHI:191 | 32.59 | 224 | 106 | 8 | 663 | 842 | 552 | 774 | 2E-21 | 96.7 |

**Supplementary material, table S7: Putative secretory Zn-inding proteins of *Xtu* probably involved in host pathogen interactions**

| **S. No.** | **Sequence ID of putative Zn-binding proteins** | **Signal Peptide present** | **Position of Tat Motif** | **Clevage site of Signal/Tat peptide** | **SecP score ofputative Zn-binding proteins above 0.5** | **No. of Transmembrane Helix** |
| --- | --- | --- | --- | --- | --- | --- |
| 1 | WP_003469868.1 | Y | - | 23 | - | - |
| 2 | WP_003469871.1 | Y | - | 26 | - | - |
| 3 | WP_003470527.1 | Y | - | 18 | - | - |
| 4 | WP_004426584.1 | Y | - | 26 | - | - |
| 5 | WP_038238706.1 | Y | - | 23 | - | - |
| 6 | WP_038239202.1 | Y | - | 17 | - | - |
| 7 | WP_047325488.1 | Y | - | 32 | - | - |
| 8 | WP_080964854.1 | Y | - | 26 | - | - |
| 9 | WP_003465625.1 | - | - | - | 0.891474 | One |
| 10 | WP_003466231.1 | - | Starting at position 14 | - | - | - |
| 11 | WP_004425530.1 | - | Starting at position 24 | - | - | - |
